# Motorized Macrocycle: A Photo‐responsive Host with Switchable and Stereoselective Guest Recognition

**DOI:** 10.1002/anie.202104285

**Published:** 2021-06-15

**Authors:** Yue Liu, Qi Zhang, Stefano Crespi, Shaoyu Chen, Xiu‐Kang Zhang, Tian‐Yi Xu, Chang‐Shun Ma, Shang‐Wu Zhou, Zhao‐Tao Shi, He Tian, Ben L. Feringa, Da‐Hui Qu

**Affiliations:** ^1^ Key Laboratory for Advanced Materials and Joint International Research Laboratory of Precision Chemistry and Molecular Engineering Feringa Nobel Prize Scientist Joint Research Center Frontiers Science Center for Materiobiology and Dynamic Chemistry Institute of Fine Chemicals School of Chemistry and Molecular Engineering East China University of Science and Technology Shanghai 200237 China; ^2^ Centre for Systems Chemistry Stratingh Institute for Chemistry and Zernike Institute for Advanced Materials University of Groningen Nijenborgh 4 9747 AG Groningen The Netherlands

**Keywords:** capture and release, host–guest interactions, motorized macrocycles, photo-responsiveness, stereoselectivity

## Abstract

Designing photo‐responsive host–guest systems can provide versatile supramolecular tools for constructing smart systems and materials. We designed photo‐responsive macrocyclic hosts, modulated by light‐driven molecular rotary motors enabling switchable chiral guest recognition. The intramolecular cyclization of the two arms of a first‐generation molecular motor with flexible oligoethylene glycol chains of different lengths resulted in crown‐ether‐like macrocycles with intrinsic motor function. The octaethylene glycol linkage enables the successful unidirectional rotation of molecular motors, simultaneously allowing the 1:1 host–guest interaction with ammonium salt guests. The binding affinity and stereoselectivity of the motorized macrocycle can be reversibly modulated, owing to the multi‐state light‐driven switching of geometry and helicity of the molecular motors. This approach provides an attractive strategy to construct stimuli‐responsive host–guest systems and dynamic materials.

## Graphical abstract

Motorized macrocycles have been successfully synthesized by combining molecular motors with crown ether macrocycles, enabling unique photo‐switchable host–guest systems controlled by the multi‐stable unidirectional rotation of molecular motors. The switchable chiral cavity can be also used for the enantioselective recognition of guests.

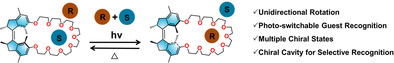

## Introduction

Since the pioneering developments in supramolecular chemistry by Cram, Lehn, and Pedersen,^[1]^ the design and synthesis of macrocycle‐based host–guest systems have taken a prominent position at the frontiers of chemistry.^[2]^ Numerous macrocycles have been synthesized, exhibiting reversible, selective, and high‐affinity binding interactions with specific guests.^[3]^ These features further expanded the broad range of applications of macrocyclic host–guest systems, including the construction of artificial molecular machines, supramolecular polymers, sensors, delivery systems, separation technology, fluorescent materials, and dynamic materials.^[4]^ Developing host–guest systems that can respond to external stimuli is particular attractive. Taking advantage of non‐covalent bonds and intrinsic switching properties provides powerful tools for designing dynamic supramolecular systems and materials which properties and functions can be modulated on command.[^4^, ^5^]

Light is widely recognized among different stimulus modes as the ideal way to control responsive materials and systems^[6]^ because it benefits from being remote, instantaneous, clean, and wavelength tunable, allowing to achieve high spatial‐temporal control. A general strategy of designing light‐responsive host–guest systems involves the introduction of photo‐isomerizing units into the structures of the macrocyclic hosts or guests, enabling the effective variation of noncovalent binding affinities by conformational transformation.[^3^, ^4^, ^7^] Many approaches have been reported using photoswitchable azobenzenes,^[8]^ stilbenes,^[9]^ spiropyrans,^[10]^ and diarylethenes,[^5a^, ^6f^, ^11^] showing their versatility in fabricating bistable photo‐responsive macrocyclic receptors. However, the design and functioning of light‐modulated host–guest systems with multi‐stable switching possibilities remains a major challenge. Meanwhile, enabling and controlling the selectivity with different guest molecules, especially with distinct chiral guests, is critical to developing synthetic biomimetic smart host–guest systems.

Light‐driven molecular motors^[12]^ based on overcrowded alkenes have been developed as a unique family of photo‐responsive units in the last decades. Due to their intrinsic feature of performing unidirectional rotation, molecular motors have been explored as robust and versatile functional units to fabricate many photo‐responsive materials, including liquid crystals,^[13]^ soft actuators,[^6b^, ^14^] gels,^[15]^ foams,^[16]^ membranes,^[17]^ solid‐state surfaces,^[18]^ porous frameworks,^[19]^ and nanocars.^[20]^ The intrinsic axial chirality and photoswitching ability of molecular motors, resulting in multiple stable chiral states with precise control over the sequence of chiral isomer formation, are the key features of asymmetric catalysts and phosphate receptors with adaptive chirality.^[21]^ These fascinating applications stimulated the introduction of molecular motors into macrocyclic host–guest systems, which might give access to an intriguing family of intrinsic motorized macrocycles that can dynamically modulate their guest binding ability and chiral recognition by unidirectional rotation.

**Figure 1:**
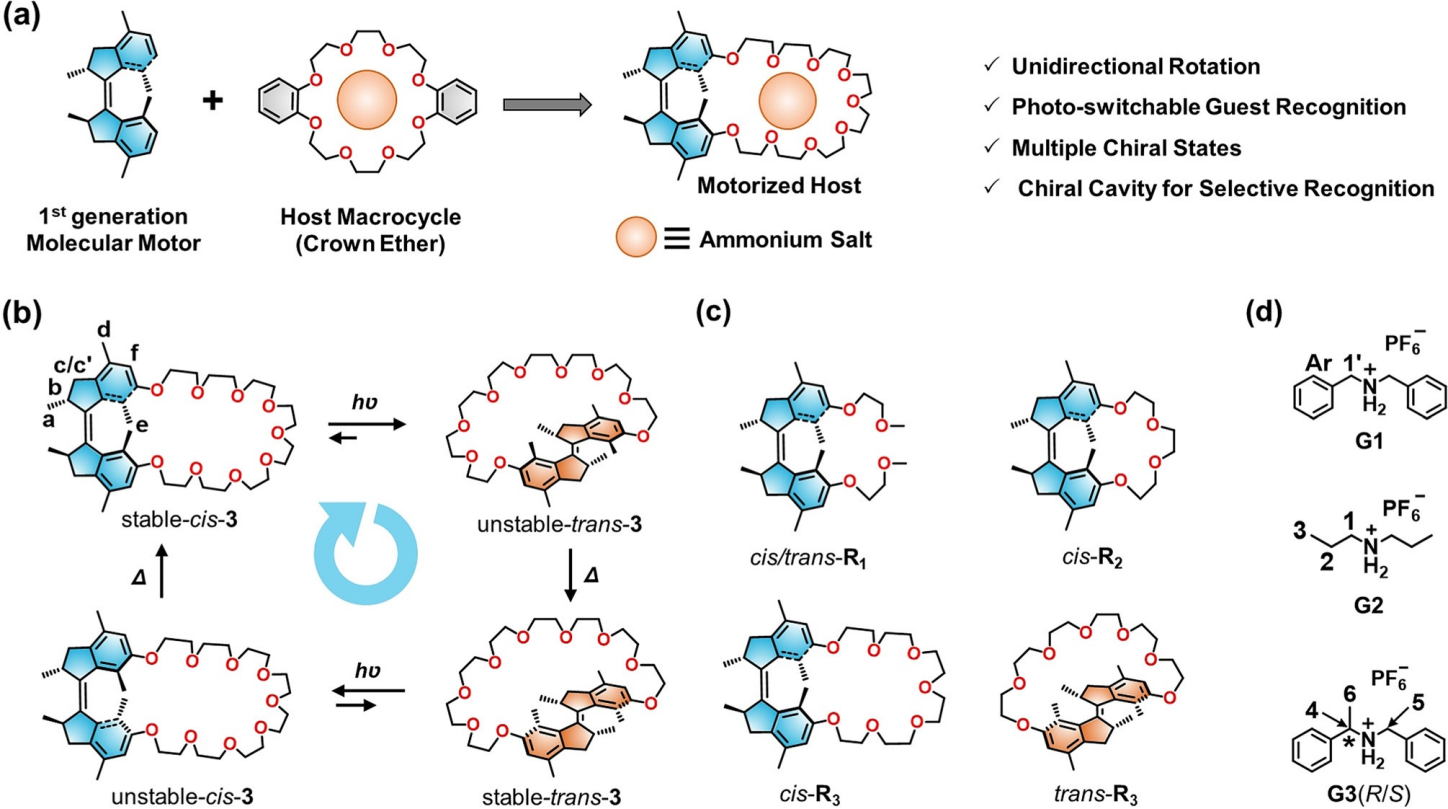
a) Representation of the structure of the novel motorized macrocycle. b) The rotation in an intramolecular confined space of motorized macrocycle **3**. c) Reference compounds. d) Guest molecules.

Here we report the combination of molecular motors with crown‐ether‐based host–guest chemistry in a system that functions as photo‐responsive host with switchable, multiple and reversible chiral guest recognition. (Figure 1) A series of motorized macrocycles was synthesized by intramolecular cyclization of first‐generation molecular motors with oligoethylene glycol chains. Studying different oligoethylene glycol chain lengths, it was established that the incorporation of the motor unit into the macrocycle offers a robust way to simultaneously enable the photochemically driven rotation of the embedded molecular motors without compromising their unidirectional rotary motion and formation of multiple states as well as effective host–guest binding. Notably, the proper size of the macrocycle allows the movement of one half of the core motor unit to pass unhindered through the macrocyclic ring during the 360 degree rotary cycle. An important feature is that the enantiopure motorized macrocycles exhibit distinct chiral states and excellent stereoselectivity with chiral guest molecules. Combined with the ability of the motorized macrocycles to invert chirality by an external trigger (i.e. light or heat), this unique host–guest system controlled by molecular motors can be a starting point to design more complex mechanically interlocked molecules and functional molecular machines.

## Results and Discussion

The motorized macrocycles, that is, the host molecules, were designed with a first‐generation molecular motor core (Figure 1 a). Functionalization with ethylene glycol chains of different lengths formed various crown‐ether rings by one‐pot intramolecular etherification‐cyclization reactions. The synthesis is summarized in the Supporting Information, Scheme S1. Specifically, the diphenol motor, that is, stable*‐cis*/*trans*‐**2**, was reacted with ethylene glycol ditosylates in the presence of cesium carbonate in acetonitrile to afford stable*‐cis*/*trans* motorized macrocycles (**3**, **R_1_**–**R_3_**, Figure 1 b,c). The yield of the stable*‐trans* motorized macrocycle was lower compared to the stable‐*cis* one, which might be attributed to the enhanced strain in the *trans* isomer. For example, the yields of stable*‐trans*‐**3** and stable*‐cis*‐**3** were 12 % and 54 %, respectively. Detailed procedures of the synthesis are provided in the Supporting Information. The molecular structures were fully characterized by ^1^H, ^13^C NMR spectroscopies, and high‐resolution mass spectrometry (HR‐MS; Supporting Information, Figures S50–S71).

**Figure 2:**
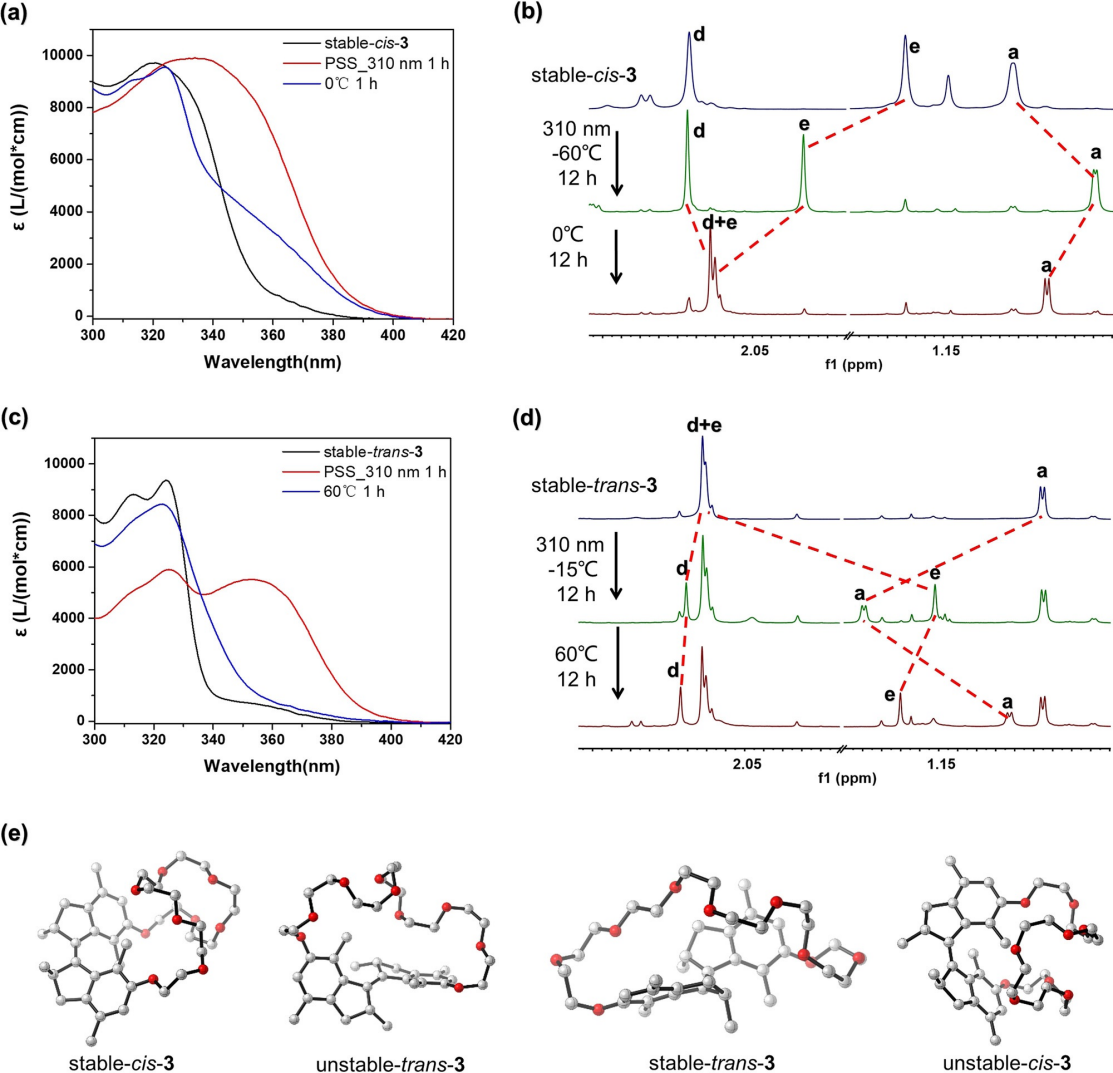
a) UV/Vis absorption spectra of stable*‐cis*‐**3** after the photoisomerization and THI processes (THF). b) Partial ^1^H NMR spectra of stable*‐cis*‐**3** (600 MHz, 203 K, CD_2_Cl_2_, 5 mM) during the photoisomerization and THI processes. c) UV/Vis absorption spectra of stable*‐trans*‐**3** during the photoisomerization and THI processes (THF). d) Partial ^1^H NMR spectra of stable*‐trans*‐**3** (600 MHz, 203 K, CD_2_Cl_2_, 5 mM) during the photoisomerization and THI processes. For the proton assignment, see Figure 1 b. e) The energy minimized optimized geometries of motorized macrocycle **3**.

The unidirectional rotation of the motorized macrocycles, involving a four‐step rotary cycle comprising two photochemical isomerizations and two thermal helix inversion (THI) steps (Figure 1 b), were studied by UV/Vis absorption and ^1^H NMR spectroscopies (Figure 2 a–d, for more details, see Figures S1–S3). A tetrahydrofuran (THF) solution of stable‐*cis*‐**3** showed a distinctive absorption peak at 320 nm (Figure 2 a), which red‐shifted to 340 nm upon irradiation (310 nm) at −60 °C for 1 h with a clear isosbestic point (Figure 2 a; Supporting Information, Figure S1a), revealing the selective photoisomerization from stable‐*cis*‐**3** to unstable‐*trans*‐**3**.^[22]^ The resulting solution was heated at 0 °C for 1 h, leading to decreased absorption at 330–400 nm (Figure 2 a; Supporting Information, Figure S1b), which indicated the effective transformation from unstable‐*trans*‐**3** to stable‐*trans*‐**3** via the THI step.^[23]^

The isomerization process from stable‐*cis*‐**3** to stable‐*trans*‐**3** was furthermore followed by ^1^H NMR by irradiating the CD_2_Cl_2_ solution of stable‐*cis*‐**3** (310 nm, −60 °C, 12 h), and then performing the subsequent THI process at 0 °C for 12 h (Figure 2 b; Supporting Information, Figure S2a–c). Distinctive proton shifts upon photoisomerization were observed in the ^1^H NMR spectra of the synthetically pure stable‐*cis*‐**3** upon 310 nm irradiation. The downfield shift of protons H_e_ (Δ*δ*=+0.662 ppm) and upfield shift of the methyl protons H_a_ (Δ*δ*=−0.218 ppm) indicated the successful transformation of the stable*‐cis*‐**3** to the unstable*‐trans*‐**3** with a photostationary state (PSS) ratio of 86:14 (unstable*‐trans*:stable*‐cis*). The resulting unstable*‐trans*‐**3** can be quantitatively converted to stable‐*trans*‐**3** via the THI step with a characteristic set of proton shifts, i.e., H_a_ shifts from *δ*=0.749 ppm to 0.879 ppm, and H_e_ shifts from *δ*=1.914 ppm to 2.150 ppm (Figure 2 b, Figure S2b,c). The thermal parameters of the transformation from unstable‐*trans*‐**3** to stable‐*trans*‐**3** were determined by Eyring analysis, which shows the Gibbs free energy of activation (Δ*G*=88.8 kJ mol^−1^), corresponding to a half‐life of 12.6 min at 20 °C (Table 1; Supporting Information, Figure S4a), typical for related first‐generation motors.^[24]^

**Table 1:** Kinetic and thermodynamic data.

	Δ*G* ^[a]^ [kJ mol^−1^]	*t*_1/2_ at 20 °C
unstable*‐trans‐* **3**	88.8	12.6 min
unstable*‐cis‐* **3**	100.3	1.0 d
unstable*‐trans‐* **R_3_**	89.4	16.0 min
unstable*‐cis‐* **R_3_**	106.0	11.2 d

[a] The Gibbs free energy of activation (Δ*G*), analysis of these data using the Eyring equation (Δ*G*=*R* *T*[ln(*k*_B_/*h*)−ln(*k*/*T*)], where *R*, *h* and *k*_B_ correspond to the gas, Planck and Boltzmann constants, respectively. The rate constants *k* of the first‐order decay at different temperatures were obtained using the equation *A*/*A* ^0^=e^−*kt*^ by UV/Vis absorption changes at 360 nm.

The subsequent two‐step transformation from stable‐*trans*‐**3** to stable‐*cis*‐**3** was also followed using UV/Vis and ^1^H NMR spectroscopy. After irradiating the THF solution of the purified stable‐*trans*‐**3** (310 nm, −15 °C), a new absorption band appears at around 360 nm (Figure 2 c; Supporting Information, Figure S1c). Meanwhile, proton H_a_ shifts downfield from *δ*=0.882 ppm to 1.352 ppm, accompanying with an upfield shift of proton H_e_ from *δ*=2.153 ppm to 1.160 ppm, in a CD_2_Cl_2_ solution of the stable‐*trans*‐**3**, demonstrating the photochemical formation of unstable‐*cis*‐**3** (PSS ratio 47:53 (unstable‐*cis*:stable‐*trans*); Figure 2 d; Supporting Information, Figure S3a,b).^[21a]^ Subsequently, heating of the resulting solutions resulted in the bleaching at 360 nm in the UV/Vis spectra (Figure 2 c; Supporting Information, Figure S1d) as well as an upfield shift of the methyl proton H_a_ (Δ*δ*=−0.383 ppm) and a downfield shift of H_e_ (Δ*δ*=+0.091 ppm) in the ^1^H NMR spectra. These observations are in accordance with the transformation from unstable‐*cis*‐**3** to stable‐*cis*‐**3** (Figure 2 d; Supporting Information, Figure S3b,c). Utilizing the Eyring analysis, the Gibbs free energy of activation from unstable‐*cis*‐**3** to stable‐*cis*‐**3** was determined as Δ*G*=100.3 kJ mol^−1^, corresponding to a half‐life of 1.0 d at 20 °C (Table 1; Supporting Information, Figure S4b). Combining all experimental data, it can be concluded that the unidirectional rotation capability in the macrocycles *trans‐*/*cis*‐**3** motor is fully maintained.

To better understand the structure of these macrocycles, we optimized the structures of the ground state minima of all isomers of macrocycle **3** (reflecting the four states in the rotary cycle) at the PW6B95D3/def2‐SVP level of theory, modelling the DCM contribution with the implicit SMD solvation method (Figure 2 e). The geometries, subject to the DFT analysis, were preliminarily screened with a conformational analysis (for details, see the Supporting Information). The dihedral angle (*θ*) of the benzene ring in the molecular motor unit was determined (Supporting Information, Table S1). As expected, the *cis* isomers bear smaller dihedral angles (7.8° for stable‐*cis*‐**3** vs. 159.4° for stable‐*trans*‐**3**), allowing the flexible crown ethers to form an accessible semicircular structure which might facilitate the noncovalent recognition with the guest. The distance between the pair of aryl‐methyl protons H_e_ in the *cis* isomer was relatively small (3.48 Å in the stable *cis* form vs. 5.91 Å in stable‐*trans*‐**3**), which was consistent with the upfield shift of these protons after photoisomerization of **3** from the stable‐*trans* isomer to unstable‐*cis* isomer.

Several reference molecules, including *cis‐*/*trans*‐**R_1_**, *cis*‐**R_2_** and *cis‐*/*trans*‐**R_3_**, were designed to understand how the presence of the macrocycle affects the rotation properties of the motors (for details, see the Supporting Information). These include an acyclic structure with pending ethylene glycols (**R_1_**) and cyclic analogues with different ring sizes (**R_2_**, **R_3_**). The reference compounds *trans‐*/*cis*‐**R_1_** and *trans‐*/*cis*‐**R_3_** exhibited consistent unidirectional rotation capability, as confirmed by similar spectral variations observed in ^1^H NMR and UV/Vis studies (Supporting Information, Schemes S2,S3, Figure S5–S9) as with the parent motor **3**. Compared to motorized macrocycle **3**, **R_3_** functionalized with a shorter glycol chain, that is, a hexaethylene glycol, showed a higher Gibbs free energy of activation, which corresponded to a longer half‐life of the unstable isomer and slower THI steps (Table 1; Supporting Information, Figure S10). When the molecular motor was functionalized with a triethylene glycol chain, that is, motorized macrocycle **R_2_**, the stable‐*cis* isomer was obtained, while the cyclization reaction of stable‐*trans*‐**2** with triethylene glycol chain was inhibited as indicated by the absence of corresponding products in ESI‐MS (Supporting Information, Figure S65). The absorption band of stable‐*cis*‐**R_2_** also exhibited a red‐shift when irradiated with 310 nm light, while the absorption band quickly recovered at room temperature (Supporting Information, Figure S11). The ^1^H NMR spectra showed no signal change (Supporting Information, Figure S12) owing to the fast recovery. A single‐crystal structure of *cis*‐**R_2_** (Supporting Information, Figure S13, for more details, see Table S2)^[31]^ showed that the dihedral angle (*θ*) between rotor and stator was only 42.7°, indicating that **R_2_** may be locked in the *cis* configurations due to the short crown‐ether ring. Combining all data, it can be concluded that the larger macrocyclic motors **3** and **R_3_** perform uncompromised unidirectional rotations.

**Figure 3:**
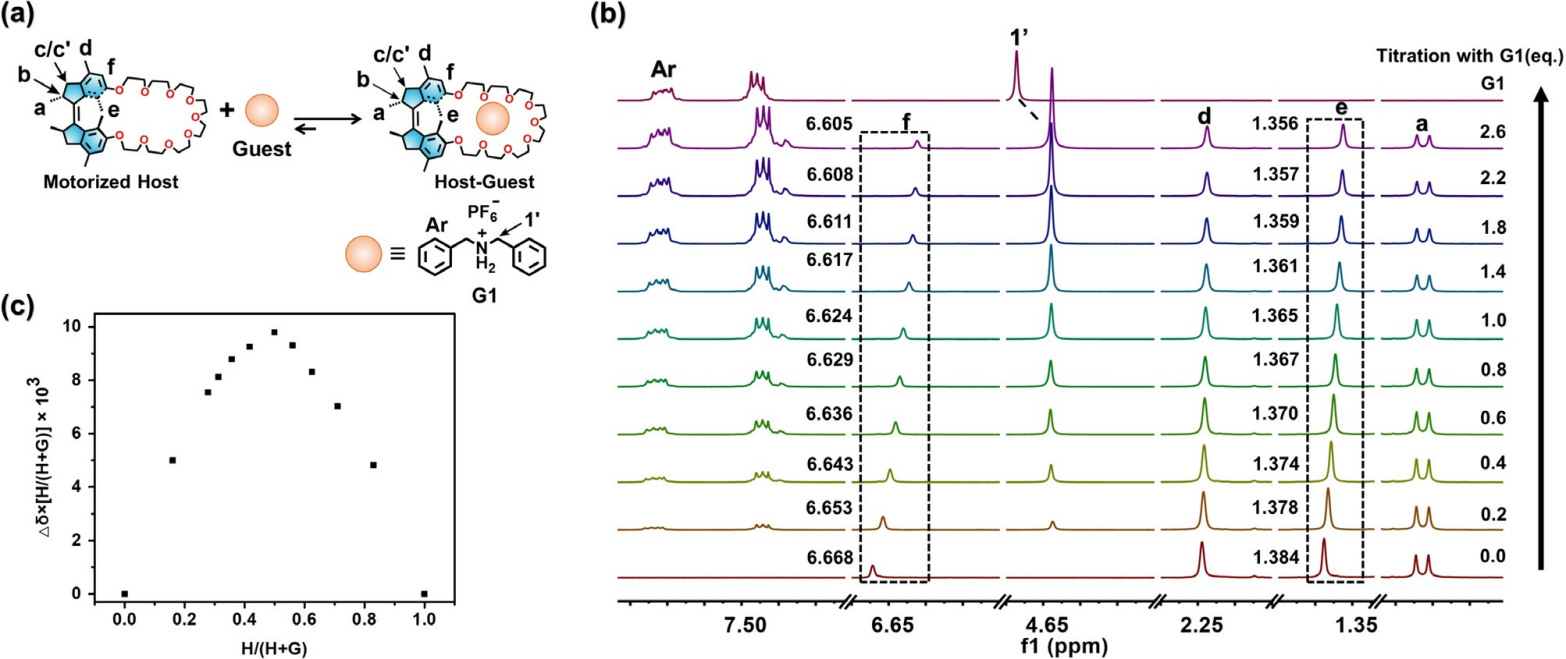
a) Representation of the guest recognition. b) Partial ^1^H NMR spectra of stable*‐cis*‐**3** (5 mM, 400 MHz, 293 K, [D_6_]acetone) upon the stepwise addition of **G1** and c) Job's plot based on the proton shift of H_f_ in [D_6_]acetone.

The host–guest chemistry of crown ethers is well‐established, and among the family of guest molecules for crown ethers, ammonium salts are remarkably versatile because of their high‐affinity mediated by hydrogen bonds and ion‐dipole interactions.^[25]^ Hence, two ammonium salts, **G1** and **G2** (Figure 1 d), were chosen as guest molecules to investigate the host–guest recognition with the motorized macrocycles. Upon addition of **G1**, characteristic proton shifts are observed in the ^1^H NMR spectra of stable‐*cis*‐**3** ([D_6_]acetone solution, Figure 3 b).^[26]^ Proton H_f_ of stable‐*cis*‐**3** shifted upfield with Δ*δ* of −0.119 ppm (Supporting Information, Figure S14). Protons H_Ar_ and H_1′_ exhibited strong coupling with protons H_crown ether_, as evident from the two‐dimensional nuclear Overhauser effects spectroscopy (NOESY; Supporting Information, Figure S15a). Moreover, a base peak at *m*/*z*=880.5356, corresponding to the [stable‐*cis*‐**3** ⊃ **G1**] ion, was observed in the ESI spectra (Supporting Information, Figure S15b). The results demonstrate the formation of the host–guest system [stable‐*cis*‐**3** ⊃ **G1**]. To quantify the binding ratio and binding constant (*K*_a_) of this host–guest system, a stable‐*cis*‐**3** solution was titrated with increasing equivalents of **G1** and followed by ^1^H NMR spectroscopy.^[27]^ The binding ratio between stable‐*cis*‐**3** and the guest **G1** was determined as 1:1 by Job's plot analysis^[28]^ (Figure 3 b,c; Supporting Information, Figure S15c). An approximate value of *K*_a_=219.0 M^−1^ was obtained by varying the ratio of stable‐*cis*‐**3** with respect to **G1** in [D_6_]acetone (Figure 3 b, for more details, see the Supporting Information, Figure S15c). On the other hand, when guest **G1** was added to the stable‐*trans*‐**3** solution under identical conditions, no proton shift or typical *m*/*z* peak for the complex was observed in the ^1^H NMR and mass spectra (Supporting Information, Figure S16), indicating the absence of sufficiently strong host–guest interactions between **G1** and *trans*‐**3**. Based on the optimized geometries (Figure 2 e; Supporting Information, Table S1), the distinctive difference in binding affinity between *trans*‐**3** and *cis*‐**3** with **G1** can be attributed to the conformation change of crown ether. The crown ether part of *trans*‐**3** is stretched into a more linear conformation, which is obviously disadvantageous for the cavity formation of the crown ether unit essential for guest binding.

Similar to **3**, [stable‐*cis*‐**R_3_** ⊃ **G1**] was obtained in [D_6_]acetone with a binding constant *K*_a_ of 162.4 M^−1^, and the HRMS showed a major peak of the host–guest system at *m*/*z*=792.4838 (Supporting Information, Figure S17). In contrast, the binding constant of stable‐*trans*‐**R_3_** was less than 1 M^−1^ (Supporting Information, Figure S18). The binding constants of these macrocycles (**3**, **R_2_** and **R_3_**) with the guest **G2** were also obtained (Table 2, for details, see the Supporting Information, Figure S19–S23). These results clearly demonstrated significant differences in the host–guest recognition of the motorized macrocycles between *cis* and *trans* isomers, indicating the possibility of in situ guest capture or release by changing the geometrical configurations of the motorized macrocycles.

**Table 2:** Binding constants of guests to the macrocycles (*K*_a_, M^−1^ in [D_6_]acetone).

		Host Macrocycles				
		*cis*‐**3**	*trans*‐**3**	*cis*‐**R_3_**	*trans*‐**R_3_**	*cis*‐**R_2_**
Guests	**G1**	219.0	<1	162.4	<1	<1
	**G2**	28.3	<1	48.8	<1	<1

**Figure 4:**
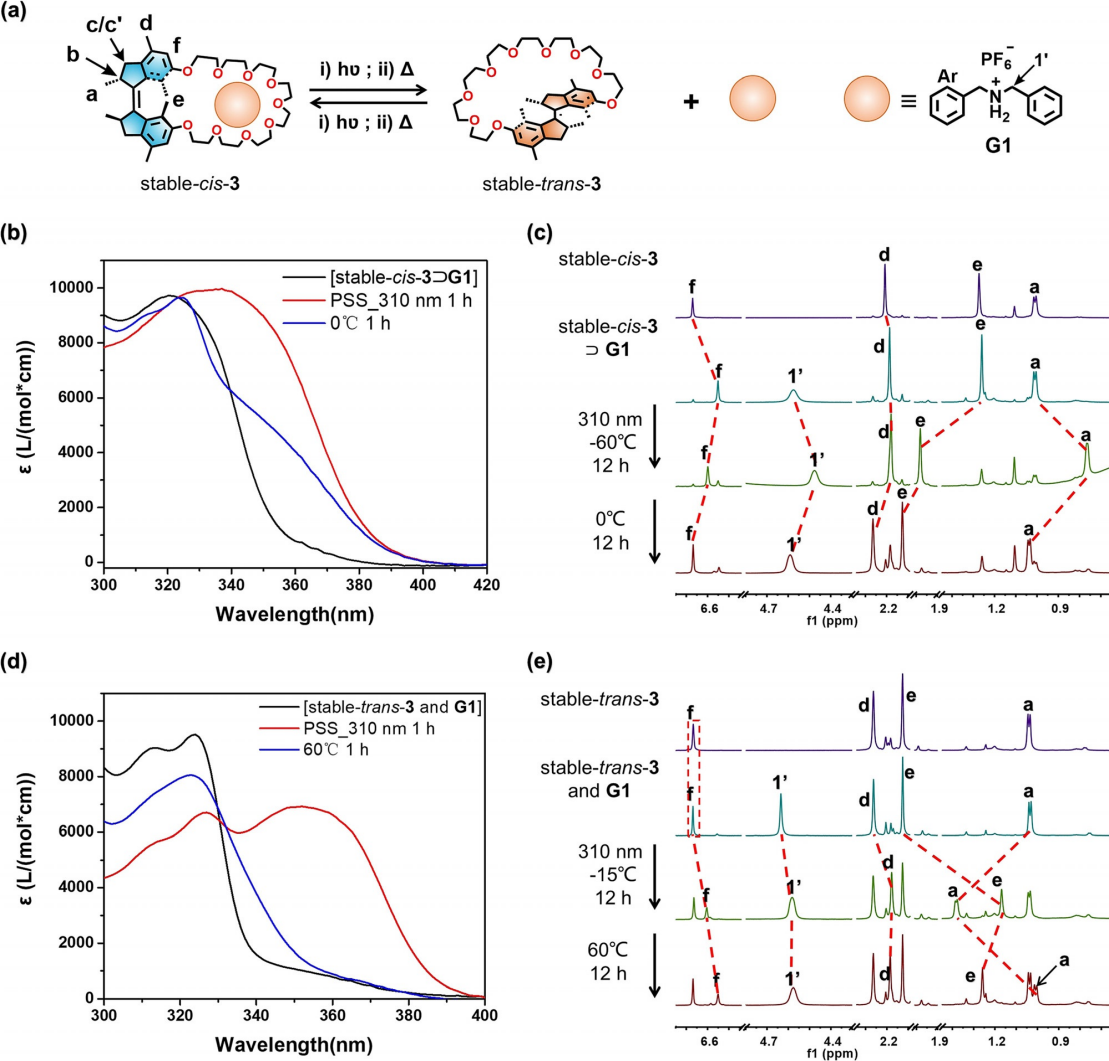
a) Representation of the guest capturing/releasing procedures of motorized macrocycle **3**. b) UV/Vis absorption spectra of the host–guest system before/after photo‐ and thermal isomerization (THF): from [stable*‐cis*‐**3** ⊃ **G1**] to [stable‐*trans*‐**3** and **G1**]. c) Partial ^1^H NMR spectra during the isomerization processes of the host–guest system (600 MHz, 203 K, [D_6_]acetone, 5 mM), starting from [stable*‐cis*‐**3** ⊃ **G1**]. d) UV/Vis absorption spectra of the host–guest system before/after photo‐ and thermal isomerization (THF): from [stable‐*trans*‐**3** and **G1**] to [stable*‐cis*‐**3** ⊃ **G1**] and e) partial ^1^H NMR spectra during the isomerization processes of the host–guest system (600 MHz, 203 K, [D_6_]acetone, 5 mM), starting from [stable‐*trans*‐**3** and **G1**].

The rotation of the motorized macrocycles accessing multiple states in the host–guest system is essential to obtain the in situ dynamic guest capture or release controlled by external light and heat stimuli. In previous studies, we showed that substituents in the molecular motor might affect its motion owing to the presence of intramolecular interactions.^[29]^ To study how the guest combination affects the rotation ability of the macrocyclic motors, structural changes of [stable‐*cis*‐**3** ⊃ **G1**] upon irradiation and heating were investigated by UV/Vis and ^1^H NMR spectroscopy (Figure 4; for more details, see the Supporting Information, Figures S24–S27).

The host–guest system of [stable‐*cis*‐**3** ⊃ **G1**] in [D_6_]acetone was irradiated with 310 nm light at −60 °C for 12 h, leading to the noticeable downfield shifts of protons H_f_ (Δ*δ*=+0.048 ppm) and H_e_ (Δ*δ*=+0.721 ppm) and upfield shifts of protons H_1′_ (Δ*δ*=−0.099 ppm) and H_a_ (Δ*δ*=−0.249 ppm) in ^1^H NMR spectra (Figure 4 c, Figure S25). Subsequently, after heating the system in the dark at 0 °C for 12 h, protons H_f_, H_1′_, H_e_ and H_a_ shifted downfield with Δ*δ* of +0.069 ppm, +0.115 ppm, +0.145 ppm, and +0.277 ppm, respectively (Figure 4 c; Supporting Information, Figure S25). The proton shifts of H_d_, H_e_ and H_a_ indicated the transformations from stable‐*cis*‐**3** to unstable‐*trans*‐**3** and subsequently stable‐*trans*‐**3** in the [stable‐*cis*‐**3** ⊃ **G1**] upon irradiation followed by heating (Figure 4 c; Supporting Information, Figure S25). Meanwhile, the proton shifts of H_f_ and H_1′_ suggested the release of guest **G1** (Figure 4 c; Supporting Information, Figure S25), retaining the spectrum of *trans*‐**3** (no guest binding). The spectral variations in the UV/Vis spectra (Figure 4 b) were consistent with those in Figure 2 a, further indicating the 180° rotation of motorized macrocycle **3**, that is, from stable‐*cis*‐**3** to stable‐*trans*‐**3**, after forming the host–guest complex with **G1**. The results demonstrate that stable‐*cis*‐**3** retained its ability to undergo rotational motion after the formation of [stable‐*cis*‐**3** ⊃ **G1**], allowing for the controllable release of the guest **G1**.

To explore photo‐controlled guest capture, the 1:1 mixture solution of stable*‐trans*‐**3** and **G1** was irradiated with 310 nm light at −15 °C, followed by heating at 60 °C. The structural changes during the photoirradiation and heating processes were also analyzed by ^1^H NMR and UV/Vis spectroscopies. The proton shifts of H_a_ (from *δ*=1.04 ppm to 1.386 ppm and 1.014 ppm) and H_e_ (from *δ*=2.126 ppm to 1.169 ppm and 1.258 ppm) indicate the transformations from stable*‐trans*‐**3** to unstable‐*cis*‐**3** and stable‐*cis*‐**3** (Figure 4 e; Supporting Information, Figure S26). Moreover, upfield shifts of proton H_f_ and H_1′_ revealed the capture of guest **G1** when stable*‐trans*‐**3** is converted to stable‐*cis*‐**3** upon photoisomerization and THI processes (Figure 4 e; Supporting Information, Figure S26). Through Eyring analysis (Supporting Information, Figure S27), the Gibbs free energy of activation and half‐life time of the host–guest system were obtained, as shown in Table 3. Compared to **3** in the absence of **G1**, slightly higher values of Gibbs free energy of activation and longer half‐lives of both unstable‐*trans*‐**3** in the presence of **G1** and [unstable‐*cis*‐**3** ⊃ **G1**] (Table 1 and 3) are seen. These effects may derive from additional hydrogen bonds and ion‐dipole interactions.

**Table 3:** Kinetic and thermodynamic data.

	Δ*G* [kJ mol^−1^]	*t*_1/2_ at 20 °C
[unstable‐*cis‐* **3** ⊃ **G1**]	101.3	1.5 d
unstable*‐trans‐* **3** mixing with **G1**	88.9	13.4 min

**Figure 5:**
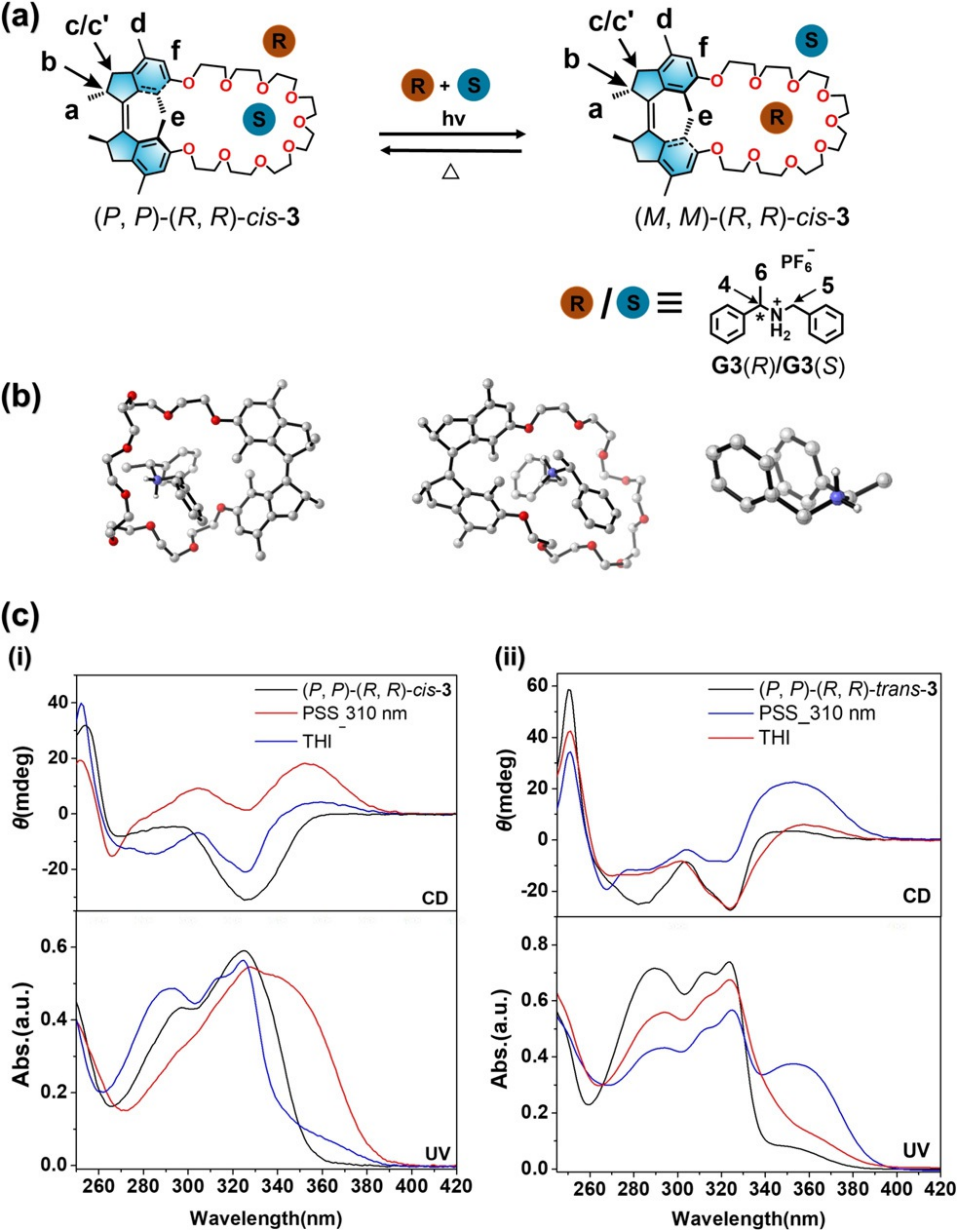
a) Representation of stereoselective guest recognition. b) B3LYP/6‐31G++(d, p) optimized geometries of [(*P*, *P*)‐(*R*,*R*)‐*cis*‐**3** ⊃ **G3**(*R*)] (left), [(*P*, *P*)‐(*R*,*R*)‐*cis*‐**3** ⊃ **G3**(*S*)] (middle) and **G3**(*R*) (right). c) (i) CD (upper) and UV/Vis absorption (lower) spectra of (*P*, *P*)‐(*R*,*R*)‐*cis*‐**3** (55 μM in CH_2_Cl_2_) before and after photoisomerization and THI: from (*P*, *P*)‐(*R*,*R*)‐*cis*‐**3** to (*P*, *P*)‐(*R*,*R*)‐*trans*‐**3**, (ii) CD and UV/Vis absorption spectra of (*P*, *P*)‐(*R*,*R*)‐*trans*‐**3** (75 μM in CH_2_Cl_2_) before and after photoisomerization and THI: from (*P*, *P*)‐(*R*,*R*)‐*trans*‐**3** to (*P*, *P*)‐(*R*,*R*)‐*cis*‐**3**.

The unique stereochemistry of the first‐generation molecular motors enables chiral transmission and dynamic inversion of chirality.^[21d]^ To investigate the stereoselective recognition ability of the motor macrocycles, enantiopure motors, (*P*, *P*)‐(*R*,*R*)‐*cis*‐**3**, (*M*, *M*)‐(*S*,*S*)‐*cis*‐**3**, (*P*, *P*)‐(*R*,*R*)‐*trans*‐**3** and (*M*, *M*)‐(*S*,*S*)‐*trans*‐**3** were separated by using chiral HPLC (Supporting Information, Figures S28–S30 and S32–S34) and the stereochemistry was confirmed by comparing the CD spectra with those of previous first generation motors reported by our group[^21c^, ^21d^] (Supporting Information, Figures S31 and S35). Non‐symmetric dibenzylamine based chiral ammonium salts, **G3**(*R*) and **G3**(*S*), were used as chiral guest molecules. (For details, see the Supporting Information). Base peaks at *m*/*z*=894.5521 and *m*/*z*=894.5519 in the ESI mass spectra (Supporting Information, Figures S36a and S38a), corresponding to the [(*P*, *P*)‐(*R*,*R*)‐*cis*‐**3** ⊃ **G3**(*R*)] and [(*P*, *P*)‐(*R*,*R*)‐*cis*‐**3** ⊃ **G3**(*S*)] ions, respectively, indicated the formation of host–guest complexes between (*P*, *P*)‐(*R*,*R*)‐*cis*‐**3** and the chiral guests. Moreover, the addition of **G3**(*R*) and **G3**(*S*), a CD_2_Cl_2_ solution (*P*, *P*)‐(*R*,*R*)‐*cis*‐**3** showed upfield shifts of protons H_f_, H_d_ and H_e_ (Supporting Information, Figures S37 and S39), which further confirms the formation of host–guest binding similar to the binding of **G1** (see Figure 3). The binding constants (*K*_*R*/*S*_) between (*P*, *P*)‐(*R*,*R*)‐*cis*‐**3** and chiral ammonium salts of **G3**(*R*/*S*) were determined by ^1^H NMR titrations in CD_2_Cl_2_. Job's plot[^27^, ^28^] analysis based on ^1^H NMR titrations, showing H/(H+G)=0.5, indicating that the ratio of (*P*, *P*)‐(*R*,*R*)‐*cis*‐**3** with both guests **G3**(*R*) and **G3**(*S*) was 1:1 (Supporting Information, Figures S36b and S38b). By fitting the titration data to a 1:1 binding model, binding constants *K_R_* of 2967.4 M^−1^ and *K_S_* of 5119.8 M^−1^ were obtained, revealing that the chiral motorized macrocycle (*P*, *P*)‐(*R*,*R*)‐*cis*‐**3** exhibited selectivity for preferred binding of the (*S*)‐enantiomer (*K_S_*/*K_R_* =1.7; Table 4). Additionally, the simulated Gibbs free energies for the host–guest binding between (*P*, *P*)‐(*R*,*R*)‐*cis*‐**3** and the chiral guests, further showed the binding preference of the host with **G3**(*S*) over **G3**(*R*) (Δ*G*=6.3 kJ mol^−1^ at the SMD(DCM)‐PW6B95D3/def2‐SVP level of theory (Figure 5 b; Supporting Information, Table S9).

**Table 4:** Binding constants of chiral guests to the macrocycle **3** (*K_R_*, *K_S_*, M^−1^ in CD_2_Cl_2_).

	(*P*, *P*)‐(*R*,*R*)‐*cis‐* **3**	(*M*, *M*)‐(*R*,*R*)‐*cis‐* **3**	(*P*, *P*)‐(*R*,*R*)‐*trans‐* **3**
**G3**(*R*)	2967.4	974.0	138.5
**G3**(*S*)	5119.8	386.5	78.2

On the other hand, (*P*, *P*)‐(*R*,*R*)‐*trans*‐**3** exhibited a poor binding affinity with the chiral guest (Table 4, for details, see the Supporting Information, Figures S40,S41). To determine the binding constant for **G3**(*R*/*S*) with (*M*, *M*)‐(*R*,*R*)‐*cis*‐**3**,^[30]^ competitive titrations to the PSS mixture (PSS_310_; (*M*, *M*)‐(*R*,*R*)‐*cis*:(*P*, *P*)‐(*R*,*R*)‐*trans*=40:60) were carried out under the same conditions. It was found that now the (*R*)‐enantiomer of **G3** binds stronger (*K_R_*/*K_S_* =2.5; Table 4, for details, see the Supporting Information, Figures S42,43). Owing to the dynamic chirality inversion of helicity of the motor macrocycle (*P*, *P*)‐(*R*,*R*)‐*cis*‐**3** to (*P*, *P*)‐(*R*,*R*)*‐trans*‐**3** (*M*, *M*)‐(*R*,*R*)‐*cis*‐**3** upon photochemical and thermal isomerization steps (Figure 5 c), this unique chiral host **3** can modulate its binding affinity and stereoselectivity (*R* or *S*) for different enantiomers of the chiral guest.

## Conclusion

In summary, we have successfully merged photochemically‐driven molecular motors with the host–guest chemistry of crown ether. The unidirectional rotation motion of molecular motors is maintained in the macrocyclic system depending on the ring size. The photoisomerization process enables the reversible modulation of the conformation of the crown ether moiety, leading to distinct photo‐switchable binding affinity with dialkylammonium guest molecules. Furthermore, the modulation of chiral recognition has been realized using the enantiopure motor macrocycle and the enantioselectivity of the chiral guest binding can be dynamically inverted by the unidirectional rotation of the motor macrocycle. We foresee that this unprecedented combination of molecular motors, macrocyclic host–guest chemistry, enabling multiple interconvertible helical states and distinct receptor structures, will be an excellent starting point for the design and construction of motorized macrocycles and more complex artificial molecular switches and machines.

## Conflict of interest

The authors declare no conflict of interest.

## Supporting information

As a service to our authors and readers, this journal provides supporting information supplied by the authors. Such materials are peer reviewed and may be re‐organized for online delivery, but are not copy‐edited or typeset. Technical support issues arising from supporting information (other than missing files) should be addressed to the authors.

Supplementary
