# Supplementary material for: Motorized Macrocycle: A Photo‐responsive Host with Switchable and Stereoselective Guest Recognition
Source: Angew Chem Int Ed Engl. 2021 Jun 15;60(29):16129–38. doi: 10.1002/anie.202104285 (PMC8361693; doi:10.1002/anie.202104285)
Supplement: Supplementary file 1 — Supplementary [file ANIE-60-16129-s001.pdf]

Angewandte Chemie
Eine Zeitschrift der Gesellschaft Deutscher Chemiker

Supporting Information

Motorized Macrocycle: A Photo-responsive Host with Switchable and Stereoselective Guest Recognition

Yue Liu[+], Qi Zhang[+], Stefano Crespi, Shaoyu Chen, Xiu-Kang Zhang, Tian-Yi Xu, Chang-Shun Ma, Shang-Wu Zhou, Zhao-Tao Shi, He Tian, Ben L. Feringa, and Da-Hui Qu**

anie_202104285_sm_miscellaneous_information.pdf

Table of Contents

1. General Methods 1
2. Synthesis Procedure 3
2.1 Synthesis of stable-*trans*-**2** and stable-*cis*-**2** 4
2.2 Synthesis of stable-*trans*-**3** 4
2.3 Synthesis of stable-*cis*-**3** 5
2.4 Synthesis of Reference Compound stable-*cis*-$\mathbf{R_1}$ 6
2.5 Synthesis of Reference Compound stable-*trans*-$\mathbf{R_1}$ 6
2.6 Synthesis of Reference Compound stable-*cis*-$\mathbf{R_2}$ 7
2.7 Synthesis of Reference Compound stable-*cis*-$\mathbf{R_3}$ 7
2.8 Synthesis of Reference Compound stable-*trans*-$\mathbf{R_3}$ 8
2.9 Synthesis of Chiral guests **G3**(*R*/*S*) 8
3. Photochemical Isomerization and THI Steps of Motorized Macrocycles. 10
3.1 UV-vis, NMR and Kinetic Studies of Macrocycle **3** 10
3.2 UV-vis and NMR Studies of the Reference Compounds 12
4. Single Crystal X-ray Analysis of *cis*-$\mathbf{R_2}$ 18
5. NMR Studies on Host-Guest Combinations 20
6. NMR and UV-vis Studies on Guest Release/Capture Procedures 30
7. Chirality Separation for Enantiomers *cis*-**3** and Enantiomers *trans*-**3** 32
7.1 Chiral HPLC of racemic compound *cis*-**3** 32
7.2 Chiral HPLC of (*M*, *M*)-(*S*, *S*)-*cis*-**3** 33
7.3 Chiral HPLC of (*P*, *P*)-(*R*, *R*)-*cis*-**3** 34
7.4 CD Spectra of (*M*, *M*)-(*S*, *S*)-*cis*-**3** and (*P*, *P*)-(*R*, *R*)-*cis*-**3** 34
7.5 Chiral HPLC of racemic compound *trans*-**3** 35
7.6 Chiral HPLC of (*M*, *M*)-(*S*, *S*)-*trans*-**3** 36
7.7 Chiral HPLC of (*P*, *P*)-(*R*, *R*)-*trans*-**3** 37
7.8 CD Spectra of (*M*, *M*)-(*S*, *S*)-*trans*-**3** and (*P*, *P*)-(*R*, *R*)-*trans*-**3** 37
8. Chiral Recognition, Binding Studies 38
9. Characterization 42
10. References 60

1. General Methods

Materials: Chemicals were received from Acros, Aldrich, Adams-beta, 3A chemicals, Energy Chemicals, Merck or TCI. All solvents were reagent grade, which were dried and distilled prior to use according to standard procedures. Flash column chromatography was performed using silica gel (Greagent, 200-300 mesh) to purified crude products. Compounds stable-*cis*/*trans*-**1**[1], **LY1**[2], **LY2**[3], **LY3**[4], **LY4**[5], **G1**[6] and **G2**[6] were synthesized and purified according to the references 1-6, respectively.

Instruments: The molecular structures were confirmed via ^{1}H NMR, ^{13}C NMR spectroscopy and high-resolution ESI mass spectrometry. ^{1}H NMR and ^{13}C NMR spectra were measured on a Brüker AV-400 and AV-600 MHz Spectrometers. The electronic spray ionization (ESI) mass spectra were obtained on a LCT Premier XE mass spectrometer and the electron impact (EI) mass spectra were measured on a Waters mass spectrometer. The UV-vis absorption spectra were obtained on a Varian Cary 100 spectrometer (1 cm, quartz cells). CD spectra were measured in a 1 cm cuvette on Jasco V-630 spectrophotometer. The UV light source was a Shanghai Gu Cun Photoelectric ZF-7D Model with a wavelength of 310 nm.

X-ray Single Crystal Analysis: Single crystals of the reference compound *cis*-$\mathbf{R_2}$ were grown by solvent evaporation[7]. *cis*-$\mathbf{R_2}$ (6 mg) was dissolved in 3 mL acetonitrile solution in a small container. After about two weeks, white color single crystals were obtained. Suitable single crystals of *cis*-$\mathbf{R_2}$ were selected and mounted on a Bruker D8 Venture diffractometer with a steady T = 170 K during data collection. The X-ray diffraction intensity data were collected at GaKα radiation (λ = 1.34139 Å). Structures were interfaced through the OLEX2[8] software. The CIF file for the crystallographic data has been deposited in the Cambridge Crystallographic Data Centre, and the CCDC number is 2063381 (*cis*-$\mathbf{R_2}$).

DFT Calculation: Computational analysis was employed to optimize the structures of the ground state minima of macrocycle-**3** (i.e. stable-*cis*-**3**, unstable-*trans*-**3**, stable-*trans*-**3**, unstable-*cis*-**3** in their respective (*R, R*) configurations), the guest **G3**, and the host-guest complexes ((*P*, *P*)-(*R*, *R*)-stable-*cis*-**3** ⊃ **G3**(*R*) and ((*P*, *P*)-(*R*, *R*)-stable-*cis*-**3** ⊃ **G3**(*S*)). Due to the dynamic nature of the molecules involved, all the structures were pre-screened using the CREST driver in the xTB software[9], using the GFN force field. In this way, the most stable conformers for each structure were picked via the default series of metadynamics and dynamics runs implemented in the driver. All the geometries were consequently optimized at the GFN2-xTB level with xTB, to afford a better qualitative ordering of the conformers. After sorting, the most stable ten conformers of each species were optimized at the PW6B95D3/def2-

SVP level, including the implicit contribution of dichloromethane via the SMD implicit solvent method. All DFT optimizations were conducted with the Gaussian 16, Rev B.01 software package[10]. All minima were confirmed to be such due to the absence of imaginary frequencies. In certain cases, some of the DFT calculations did not converge due to oscillations in the energy, a behavior found in ca. 50% of the conformers of the host guest-complexes (5/10 of (*P*, *P*)-(*R*, *R*)-stable-*cis*-**3** ⊃ **G3**(*R*) and 6/10 of ((*P*, *P*)-(*R*, *R*)-stable-*cis*-**3** ⊃ **G3**(*S*)). We could however optimize the most stable isomers found from the preliminary xTB sorting, furnishing a semi-quantitative analysis of the energies of the structures involved in the study. All the properties reported in Table S1 and S9 are Boltzmann averaged. The computational data showed a slight preference of 1.5 kcal·mol^{-1} for the ((*P*, *P*)-(*R*, *R*)-stable-*cis*-**3** ⊃ **G3**(*S*) complex over ((*P*, *P*)-(*R*, *R*)-stable-*cis*-**3** ⊃ **G3**(*R*). All xyz coordinates for all the conformers considered are provided as separate additional file.

Binding Constants: The binding constants were calculated using the method reported on website http://app.supramolecular.org/bindfit/.

Heating of the NMR samples (60 °C): Pressure resistant Schlenk Vessels were used, and the samples were sealed and heated before transferring into a NMR tube. The heating processes were conducted in a closed system, so no solvent lost was observed.

2. Synthesis Procedure

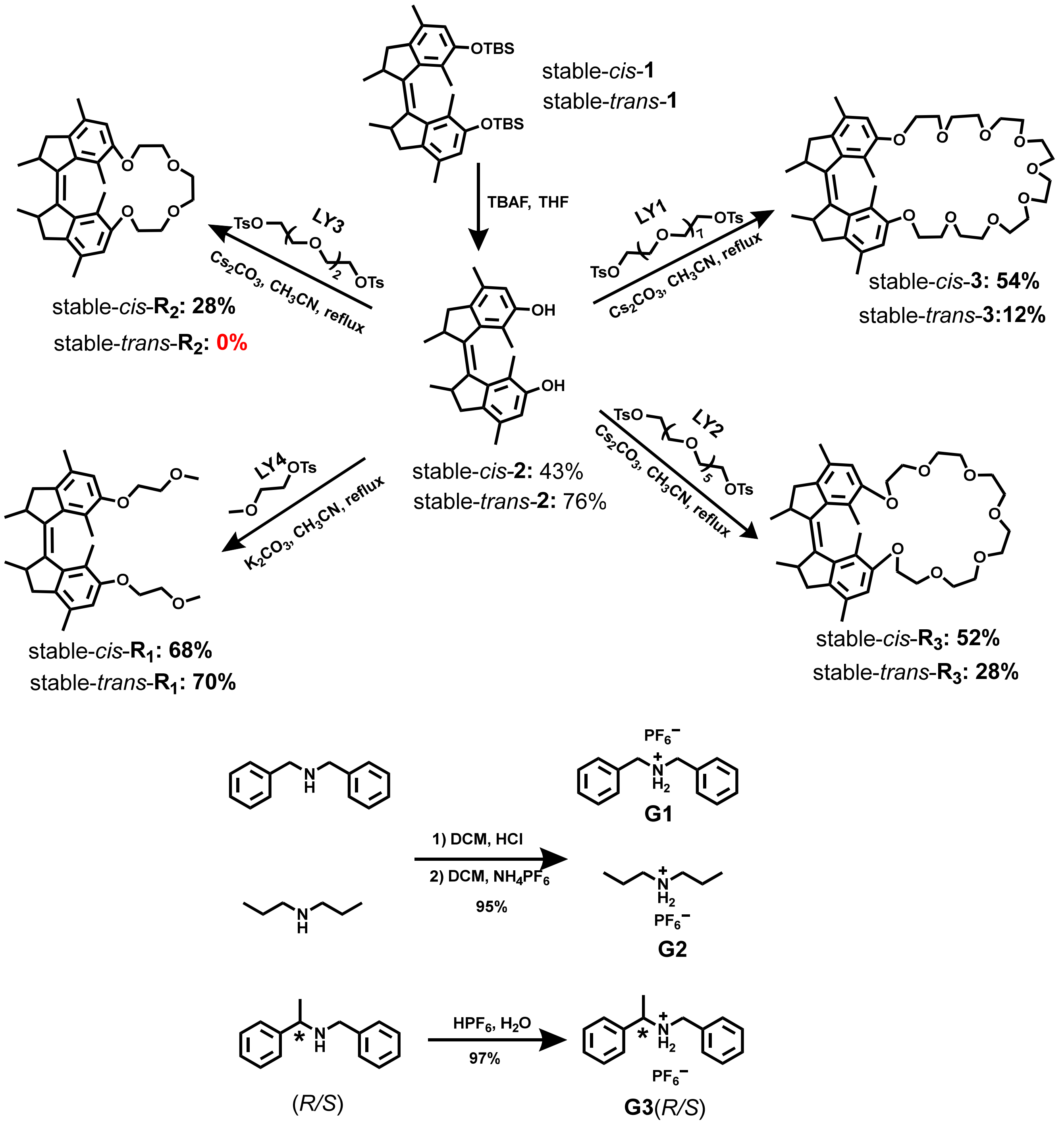

Scheme S1. The synthesis routes of the motorized macrocycles.

2.1 Synthesis of stable-*trans*-2 and stable-*cis*-2

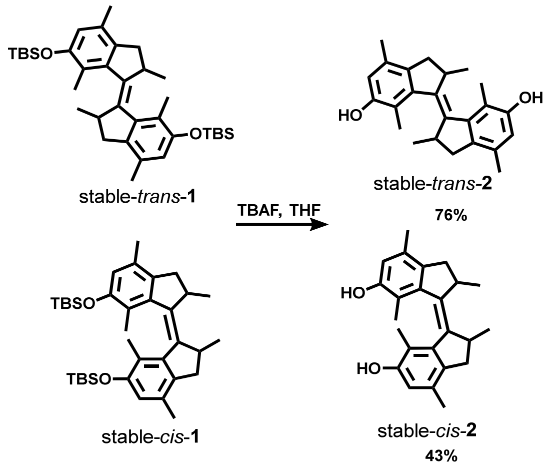

To a solution of a mixture stable-*trans*-**1** and stable-*cis*-**1** (774 mg, 1.34 mmol) (see ref. 1 for synthetic procedure) in 100 ml THF, was added 1.40 g TBAF. The mixture was stirred at room temperature for 30 min and subsequently poured into 200 mL water and extracted with ethyl acetate (EA, 3 ×50 mL). The organic layer was dried over Na_2SO_4 and concentrated under vacuum. The residue was purified by silica gel column chromatography using PE/EA (5/1) as the eluent affording 178 mg (76% based on stable-*trans*-**1**) of stable-*trans*-**2** as a creamy-white solid and 100 mg (43% based on stable-*cis*-**1**) of stable-*cis*-**2** as yellow solid.

stable-*trans*-**2:** ^{1}H NMR (400 MHz, 298 K, DMSO-d_6) δ (ppm): 8.96 (s, 2H), 6.50 (s, 2H), 2.74 (m 2H), 2.42 (dd, J = 16.0, 4.0 Hz, 2H), 2.16 (s, 6H), 2.10 (d, J = 12.0 Hz, 2H), 2.05 (s, 6H), 1.01 (d, J = 4.0 Hz, 6H). ^{13}C NMR (100 MHz, 298 K, DMSO-d_6) δ (ppm): 154.2, 141.6, 140.9, 131.6, 130.8, 117.4, 114.2, 41.5, 37.7, 19.3, 18.1, 16.1. HRMS (EI): *m*/*z* (100%) calcd for $C_{24}H_{28}O_2^{+\bullet}$ $[M]^{+\bullet}$, 348.2089; found: 348.2091.

stable-*cis*-**2**: ^{1}H NMR (400 MHz, 298 K, CD_2Cl_2) δ (ppm): 6.52 (s, 2H), 3.32 (m, 2H), 3.02 (dd, J = 16.0, 4.0 Hz, 4H), 2.38 (d, J = 16.0 Hz, 2H), 2.20 (s, 6H), 1.39 (s, 6H), 1.06 (d, J = 8.0 Hz, 6H). ^{13}C NMR (100 MHz, 298 K, CD_2Cl_2) δ (ppm): 152.9, 142.6, 141.3, 136.6, 131.8, 119.3, 115.0, 42.3, 38.4, 20.0, 18.6, 14.0. HRMS (EI): *m*/*z* (100%) calcd for $C_{24}H_{28}O_2^{+\bullet}$ $[M]^{+\bullet}$: 348.2089; found: 348.2090

2.2 Synthesis of stable-*trans*-3

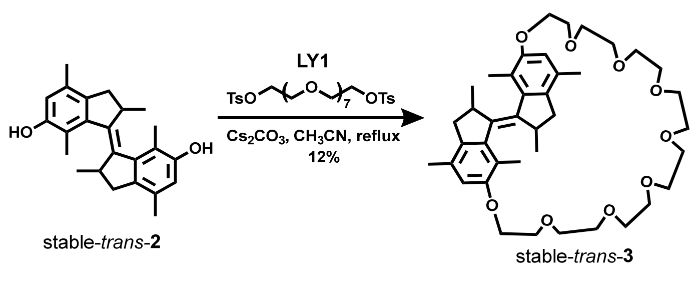

A mixuture of stable-*trans*-**3** (350 mg, 1.0 mmol), **LY1** (682.7 mg, 1.0 mmol), Cs_2CO_3 (2.0 g, 6.0 mmol) in 400 mL acetonitrile was heated at 80˚C under argon atmosphere for 14 h. After cooling to room temperature, the solvent was removed under vacuum. The residue was poured into 50 mL water followed by extraction with ethyl acetate (EA, 3 ×30 mL). The combined organic phase was dried and the solvent was removed under vacuum. The residue was purified by silica gel column chromatography using DCM/MeOH (400/1) as eluent affording 82 mg (12%) stable-*trans*-**3** as a white solid. ^{1}H NMR (400 MHz, 298 K, $(CD_3)_2CO$) δ (ppm): 6.66 (s, 2H), 4.22-4.16 (m, 4H), 3.83-3.78 (m, 4H), 3.71-3.64 (m, 4H), 3.59-3.48 (m, 20H), 2.88 (m, 2H), 2.57 (dd, J = 14.0, 5.6 Hz, 2H), 2.35 (s, 6H), 2.23 (d, J = 14.0 Hz, 2H), 2.17 (s, 6H), 1.11 (d, J = 6.4 Hz, 6H). ^{13}C NMR (100 MHz, 298 K, CD_2Cl_2) δ (ppm): 156.6, 142.7, 142.0, 134.9, 131.8, 121.9, 112.6, 71.3, 70.9, 70.9, 70.8, 70.8, 70.7, 70.1, 69.4, 43.2, 38.9, 19.3, 18.8, 16.7. HRMS (ESI): *m*/*z* calcd for $C_{40}H_{58}O_9Na^+$ $[M+Na]^+$: 705.3973; found: 705.3969.

2.3 Synthesis of stable-*cis*-3

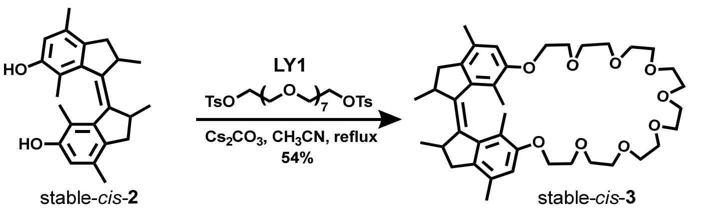

A mixture of stable-*cis*-**2** (227 mg, 0.65 mmol), **LY1** (443 mg, 0.65 mmol), Cs_2CO_3 (1.3 g, 4.0 mmol) in 300 mL acetonitrile was heated at 80˚C under argon atmosphere for 14 h. After cooling to room temperature, the solvent was removed under vacuum. The residue was poured into 100 mL water and extracted with ethyl acetate (EA, 2 × 30 mL). The combined organic phase was dried and the solvent removed under vacuum. The residue was purified by silica gel column chromatography using DCM/MeOH (30/1) as eluent affording 242 mg (54%) stable-*cis*-**3** as a clear oil. ^{1}H NMR (400 MHz, 298 K, $CDCl_3$) δ (ppm): 6.47 (s, 2H), 4.10 (m, 2H), 3.98-3.90 (m, 2H), 3.77 (t, J = 4.8 Hz, 4H), 3.69-3.64 (m, 4H), 3.58 (m, 20H), 3.25 (m, 2H), 2.96 (dd, J = 14.4, 6.0 Hz, 2H), 2.30 (d, J = 14.4 Hz, 2H), 2.17 (s, 6H), 1.30 (4, 6H), 0.99 (d, J = 6.8 Hz, 6H). ^{13}C NMR (100 MHz, 298 K, $CDCl_3$) δ (ppm): 155.6, 142.2, 140.9, 136.4, 130.5, 122.5, 112.0, 70.98, 71.0, 70.7, 70.0, 68.5, 68.4, 41.8, 38.0, 20.5, 18.9, 14.4. HRMS (ESI): *m*/*z* calcd for $C_{40}H_{58}O_9Na^+$ $[M+Na]^+$: 705.3973; found: 705.3978.

2.4 Synthesis of Reference Compound stable-*cis*-R$_1$

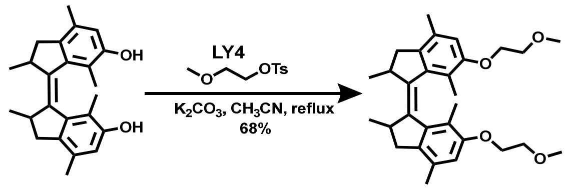

stable-*cis*-**2** stable-*cis*-**R$_1$**

A mixture of stable-*cis*-**2** (50 mg, 0.14 mmol), **LY4** (33 mg, 0.14 mmol), K_2CO_3 (48.3 mg, 0.35 mmol) in 60 mL acetonitrile was heated at 80°C under argon atmosphere for 24 h. After cooling to room temperature, the solvent was removed under vacuum. The residue was poured into 25 mL water and extracted with ethyl acetate (EA, 3 × 20 mL). The combined organic phase was dried with Na_2SO_4 and the solvent was removed under vacuum. The residue was purified by silica gel column chromatography using PE/EA (10/1) as eluent affording 45 mg (68%) stable-*cis*-**R$_1$** as a white solid. ^{1}H NMR (400 MHz, 298 K, $CDCl_3$) δ (ppm): 6.54 (s, 2H), 4.17-4.10 (m, 2H), 4.01 (m, 2H), 3.72 (t, J = 5.2 Hz, 4H), 3.43 (s, 6H), 3.32 (m, 2H), 3.03 (dd, J = 14.4, 6.4 Hz, 2H), 2.37 (d, J = 14.4 Hz, 2H), 2.24 (s, 6H), 1.40 (s, 6H), 1.06 (d, J = 6.8 Hz, 6H). ^{13}C NMR (100 MHz, 298 K, $CDCl_3$) δ (ppm): 154.6, 141.2, 139.9, 135.5, 129.4, 121.7, 111.3, 70.4, 67.5, 58.2, 40.9, 37.1, 19.4, 17.8, 13.2. HRMS (ESI): *m/z* calcd for $C_{30}H_{40}O_4Na^+$ $[M+Na]^+$: 487.2819; found: 487.2808.

2.5 Synthesis of Reference Compound stable-*trans*-R$_1$

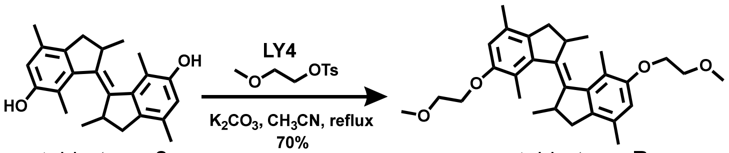

stable-*trans*-**2** stable-*trans*-**R$_1$**

A mixture of stable-*cis*-**2** (50 mg, 0.14 mmol), **LY4** (33 mg, 0.14 mmol), K_2CO_3 (48.3 mg, 0.35 mmol) in 60 mL acetonitrile was heated at 80°C under argon atmosphere for 24 h. After cooling to room temperature, the solvent was removed under vacuum. The residue was poured into 25 mL water and extracted with ethyl acetate (EA, 3 × 20 mL). The combined organic phase was dried with Na_2SO_4 and the solvent was removed under vacuum. The residue was purified by silica gel column chromatography using PE/EA (10/1) as eluent affording 47 mg (70%) stable-*trans*-**R$_1$** as a white solid. ^{1}H NMR (400 MHz, 298 K, $CDCl_3$) δ (ppm): 6.55 (s, 2H), 4.20-4.15 (m, 2H), 4.10 (m, 2H), 3.81 (t, J = 5.2 Hz, 4H), 3.50 (s, 6H), 2.88 (m, 2H), 2.59 (dd, J = 14.0, 5.6 Hz, 2H), 2.32 (s, 6H), 2.18 (s, 6H), 2.15 (d, J = 14.0 Hz, 2H), 1.08 (d, J = 6.4 Hz, 6H). ^{13}C NMR (100 MHz, 298 K, $CDCl_3$) δ (ppm): 155.1, 141.5, 140.6, 133.4, 130.2, 119.7, 110.3, 70.5, 67.1, 58.3, 41.1, 37.3, 18.3, 17.7, 15.2. HRMS (ESI): *m/z* calcd for $C_{30}H_{40}O_4Na^+$ $[M+Na]^+$: 487.2819; found: 487.2803.

2.6 Synthesis of Reference Compound stable-*cis*-R_2

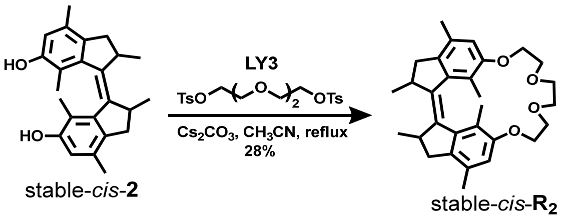

A mixture of stable-*cis*-**2** (80 mg, 0.23 mmol), **LY3** (105 mg, 0.23 mmol), Cs_2CO_3 (225 mg, 0.69 mmol) in 100 mL acetonitrile was heated at 80˚C under argon atmosphere for 24 h. After cooling to room temperature, the solvent was removed under vacuum. The residue was poured into 25 mL water and extracted with ethyl acetate (EA, 3 × 25 mL). The combined organic phase was dried with Na_2SO_4 and the solvent was removed under vacuum. The residue was purified by silica gel column chromatography using PE/EA (3/1) as eluent affording 30 mg (28%) stable-*cis*-$\mathbf{R_2}$ as a white solid. ^{1}H NMR (400 MHz, 298 K, $CDCl_3$) δ (ppm): 6.52 (s, 2H), 4.23-4.11 (m, 4H), 3.83-3.75 (m, 4H), 3.73-3.67 (m, 4H), 3.33 (m, 2H), 3.05 (dd, J = 14.8, 6.4 Hz, 2H), 2.39 (d, J = 14.8 Hz, 2H), 2.24 (s, 6H), 1.39 (s, 6H), 1.10 (d, J = 6.4 Hz, 6H). ^{13}C NMR (100 MHz, 298 K, $CDCl_3$) δ (ppm): 156.0, 142.0, 140.7, 136.2, 130.4, 123.0, 112.8, 112.8, 70.5, 69.6, 69.2, 41.3, 38.1, 20.5, 18.7, 18.7, 14.7. HRMS (ESI): *m*/*z* calcd for $C_{30}H_{38}O_4Na^+$ $[M+Na]^+$: 485.2668; found: 485.2667.

2.7 Synthesis of Reference Compound stable-*cis*-R_3

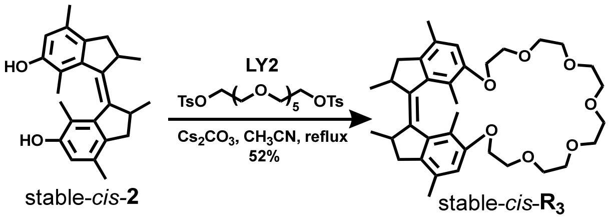

A mixture of stable-*cis*-**2** (50 mg, 0.14 mmol), **LY2** (85 mg, 0.14 mmol), Cs_2CO_3 (140 mg, 0.43 mmol) in 60 mL acetonitrile was heated at 80˚C under argon atmosphere for 24 h. After cooling to room temperature, the solvent was removed under vacuum. The residue was poured into 25 mL water and extracted with ethyl acetate (EA, 3 × 25 mL). The combined organic phase was dried with Na_2SO_4 and the solvent was removed under vacuum. The residue was purified by silica gel column chromatography using CH_2Cl_2/CH_3OH (50/1) as eluent affording 45 mg (54%) stable-*cis*-$\mathbf{R_3}$ as a clear oil. ^{1}H NMR(400 MHz, 298 K, $CDCl_3$) δ (ppm): 6.55 (s, 2H), 4.18 (m, 2H), 4.04-3.96 (m, 2H), 3.91-3.82 (m, 4H), 3.79-3.65 (m, 16H), 3.32 (m, 2H), 3.03 (dd, J = 14.8, 6.4 Hz, 2H), 2.37 (d, J = 14.4 Hz, 2H), 2.24 (s, 6H), 1.39 (s, 6H), 1.06 (d, J = 6.8 Hz, 6H). ^{13}C NMR (150 MHz, $CDCl_3$, 298 K) δ (ppm):154.6, 141.2, 139.9, 135.5,

129.5, 121.5, 111.2, 70.1, 70.0, 69.9, 69.7, 69.1, 68.0, 40.8, 37.0, 19.5, 17.8, 13.4. HRMS (ESI): *m*/*z* calcd for $C_{36}H_{50}O_7Na^+$ $[M+Na]^+$: 617.3439; found: 617.3436.

2.8 Synthesis of Reference Compound stable-*trans*-R$_3$

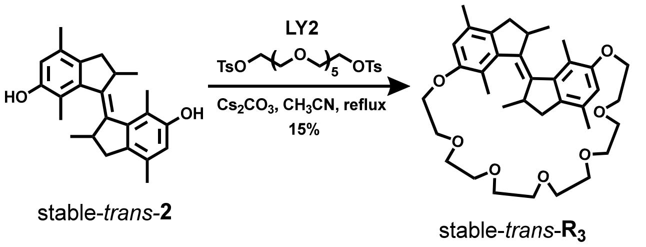

A mixture of stable-*trans*-**2** (191 mg, 0.55 mmol), **LY2** (324 mg, 0.55 mmol), Cs_2CO_3 (536 mg, 1.65 mmol) in 260 mL acetonitrile was heated at 80˚C under argon atmosphere for 36 h. After cooling to room temperature, the solvent was removed under vacuum. The residue was poured into 25 mL water and extracted with ethyl acetate (EA, 3 × 25 mL). The combined organic phase was dried with Na_2SO_4 and the solvent was removed under vacuum. The residue was purified by silica gel column chromatography using CH_2Cl_2/CH_3OH (40/1) as eluent affording 49 mg (15%) stable-*trans*-**R$_3$** as a clear oil. ^1H NMR (400 MHz, 298 K, $CDCl_3$) δ (ppm): 6.53 (s, 2H), 4.18-4.08 (m, 4H), 3.92-3.87 (m, 4H), 3.80-3.76 (m, 4H), 3.68 (m, 12H), 2.87 (m, 2H), 2.57 (dd, J = 14.4, 6.0 Hz, 2H), 2.29 (s, 6H), 2.18 (s, 1H), 2.15 (s, 6H), 2.11 (s, 1H), 1.06 (d, J = 6.4 Hz, 6H). ^{13}C NMR (100 MHz, 298 K, $CDCl_3$) δ (ppm): 156.1, 142.4, 141.6, 134.3, 131.2, 120.6, 111.2, 71.0, 70.8, 70.7, 70.1, 68.4, 42.2, 38.4, 19.2, 18.7, 16.3. HRMS (ESI): *m*/*z* calcd for $C_{36}H_{50}O_7Na^+$ $[M+Na]^+$: 617.3439; found: 617.3443.

2.9 Synthesis of Chiral guests G3(*R*/*S*)

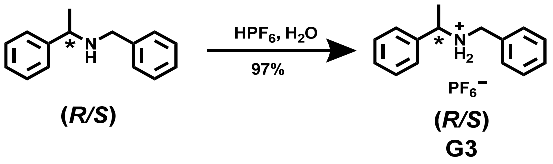

General Method: (*R*)-(+)-N-benzyl-1-phenylethylamine/(*S*)-(-)-N-benzyl-1-phenylethylamine（1 g, 4.7 mmol）was added to a 25 mL flask, and subsequently hexafluorophosphoric acid (0.83 g, 0.5 mL, 5.7 mmol) was added dropwise at 0-5°C. After 5 min, 5 mL of deionized water was added to the mixture whereupon a white solid gradually precipitated. The solid was filtered and washed with a small amount of deionized water and the obtained white solid was dried under vacuum overnight.

G3(*R*) white solid：1.94 g, 97%。^1H NMR (400 MHz, 298 K, $CDCl_3$) δ (ppm): 8.52 (s, 2H), 7.42 (s, 5H), 7.32 (s, 5H), 4.13 (s, 1H), 3.98 (d, J = 12.4 Hz, 1H), 3.81-3.72 (m, 1H), 1.63 (d, J = 6.6 Hz, 3H).

^{13}C NMR (100 MHz, 298 K, $CDCl_3$) δ (ppm): 135.5, 130.0, 129.9, 129.6, 129.5, 129.1, 127.6, 57.9, 49.4, 20.2. HRMS (ESI): *m/z* calcd for $C_{15}H_{18}N^-$ [M-PF_6^-]$^+$: 212.1434; found: 212.1437.

G3(*S*) white solid：1.94 g, 97%。^1H NMR (400 MHz, 298 K, $CDCl_3$) δ (ppm): 8.53 (s, 2H), 7.42 (s, 5H), 7.32 (s, 5H), 4.13 (s, 1H), 3.98 (d, *J* = 12.4 Hz, 1H), 3.76 (d, *J* = 12.0 Hz, 1H), 1.63 (d, *J* = 5.6 Hz, 3H). ^{13}C NMR (100 MHz, 298 K, $CDCl_3$) δ (ppm): 135.5, 130.0, 129.9, 129.6, 129.5, 129.1, 127.6, 57.9, 49.4, 20.2. HRMS (ESI): *m/z* calcd for $C_{15}H_{18}N^-$ [M-PF_6^-]$^+$: 212.1434; found: 212.1430.

3. Photochemical Isomerization and THI Steps of Motorized Macrocycles.

3.1 UV-vis, NMR and Kinetic Studies of Macrocycle 3

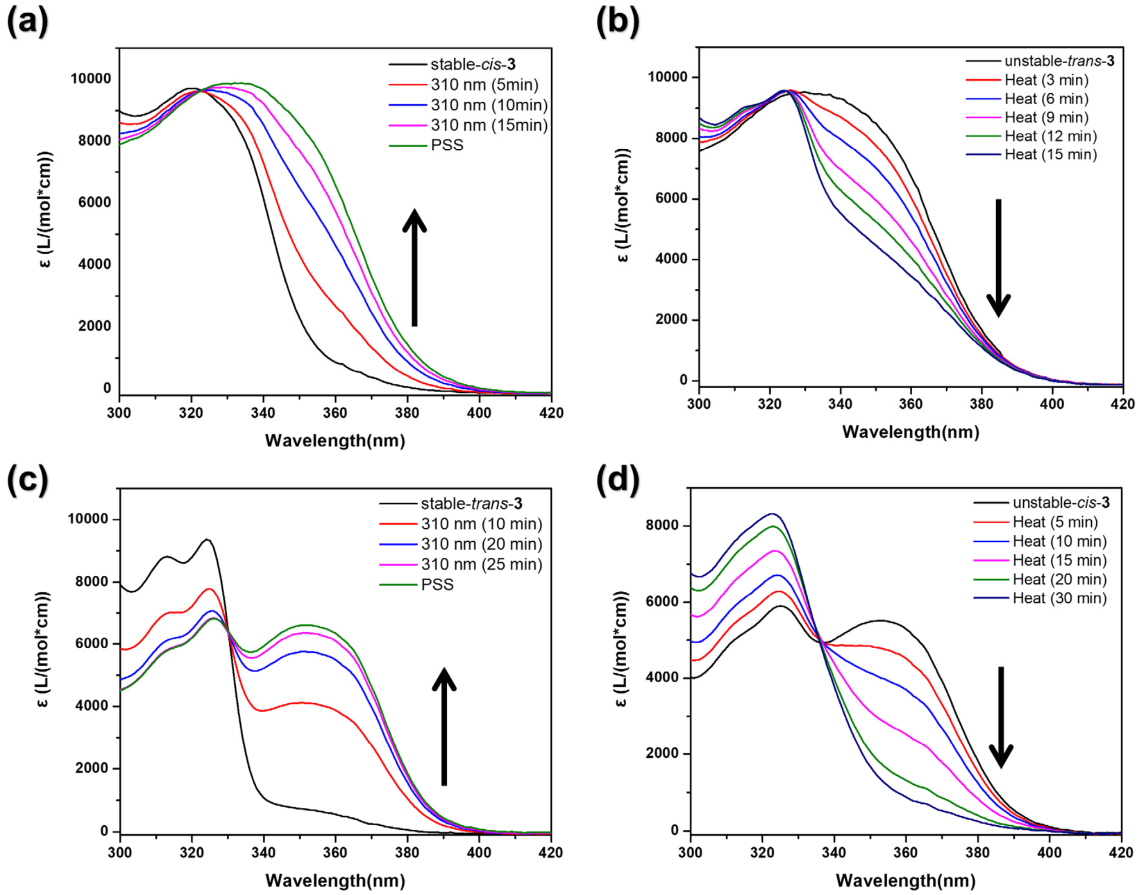

Figure S1. UV-vis absorption spectral changes in THF during the photoisomerization and THI processes, starting from: (a) stable-*cis*-**3** to unstable-*trans*-**3** (310 nm, THF, -60 ºC). (b) unstable-*trans*-**3** to stable-*trans*-**3** (THF, 0 ºC). (c) stable-*trans*-**3** to unstable-*cis*-**3**(310 nm, THF, -15 ºC). (d) unstable-*cis*-**3** to stable-*cis*-**3** (THF, 60 ºC).

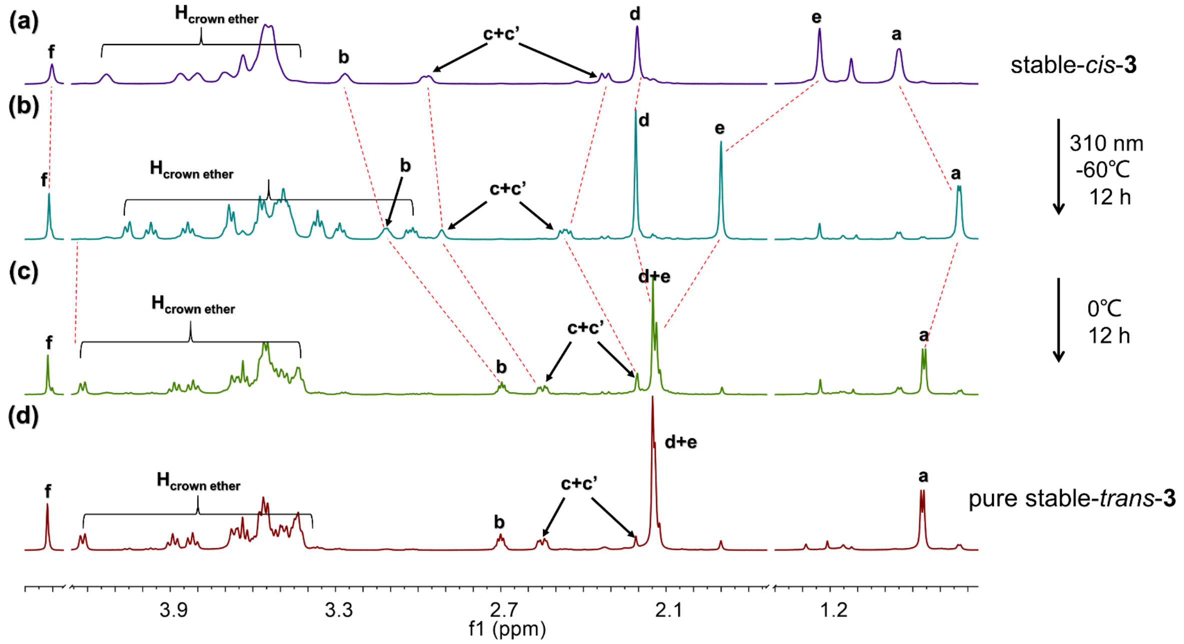

Figure S2. Partial ^{1}H NMR spectra (600 MHz, 203 K, CD_2Cl_2, 5 mM) of compounds (a) stable-*cis*-**3**. (b) unstable-*trans*-**3** upon irradiation of (a) (310 nm at -60 °C). (c) stable-*trans*-**3** upon heating the solution of (b) (0 °C). (d) The comparative spectrum of synthetic pure stable-*trans*-**3**.

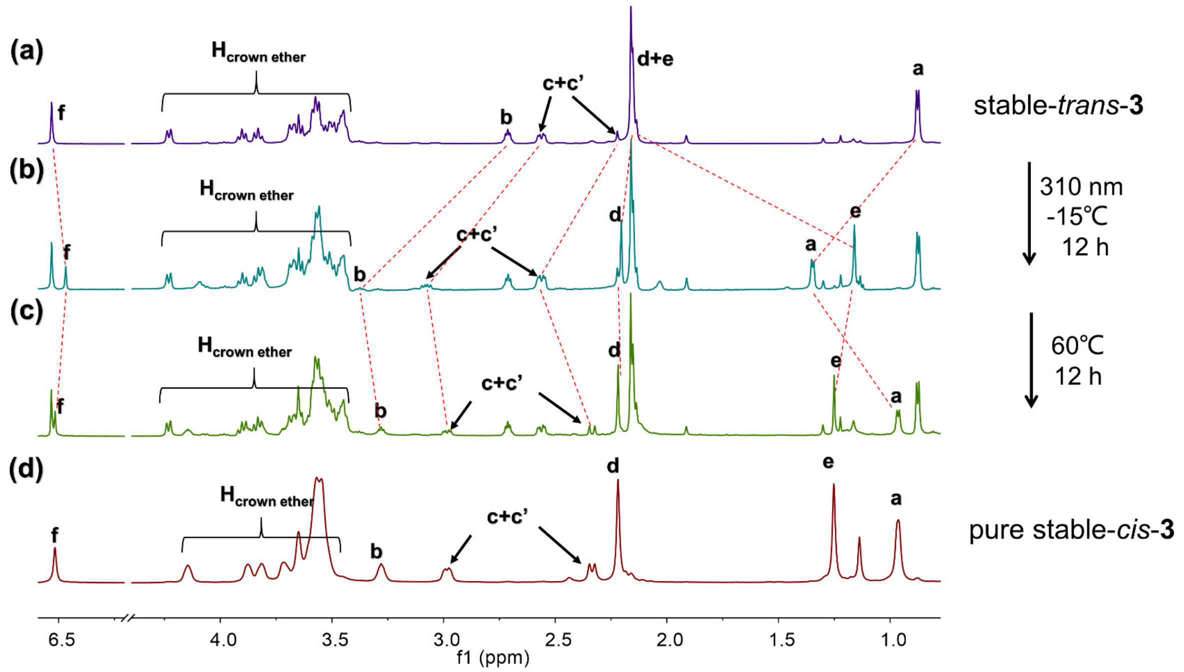

Figure S3. Partial ^{1}H NMR spectra (600 MHz, 203 K, CD_2Cl_2, 5 mM) of compounds (a) stable-*trans*-**3**. (b) unstable-*cis*-**3** upon irradiation of (a) (310 nm, -15 °C). (c) stable-*cis*-**3** upon heating the solution of (b) (60 °C). (d) The comparative spectrum of synthetic pure stable-*cis*-**3**.

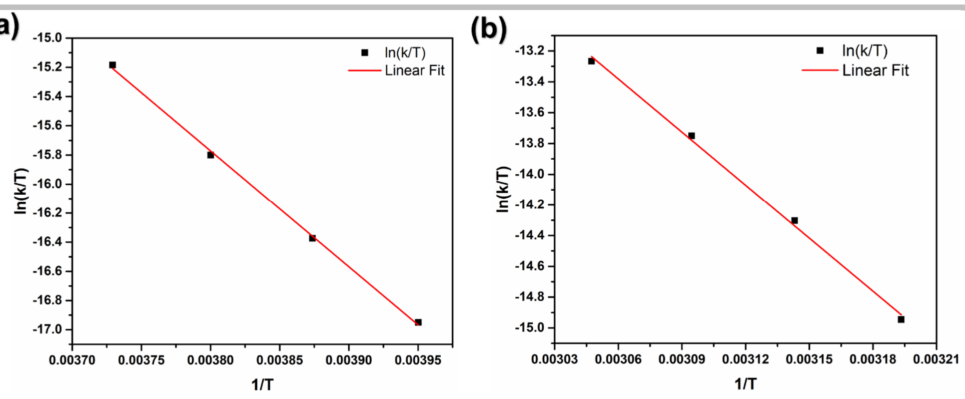

Figure S4. Kinetic studies of the thermal helix inversion steps of (a) unstable-*trans*-**3** to stable-*trans*-**3** and (b) unstable-*cis*-**3** to stable-*cis*-**3**. These isomerization steps were followed by UV absorption changes at 360 nm four different temperature conditions in THF(unstable-*trans* to stable-*trans*: -20 °C, -15 °C, -10 °C and -5 °C; unstable-*cis* to stable-*cis*: 40 °C, 45 °C, 50 °C and 55 °C).

Table S1. Boltzmann averaged energies, dihedral angles (θ) and distances between the methyl groups of the *fjord* region of macrocycle **3**.

	stable-*cis*-**3**	unstable-*trans*-**3**	stable-*trans*-**3**	unstable-*cis*-**3**
G (Ha)	-2236.4323	-2236.4234	-2236.4269	-2236.4258
θ (º)	7.75	161.29	159.35	-23.83
Me-Me (Å)	3.48	5.00	5.91	3.62

3.2 UV-vis and NMR Studies of the Reference Compounds

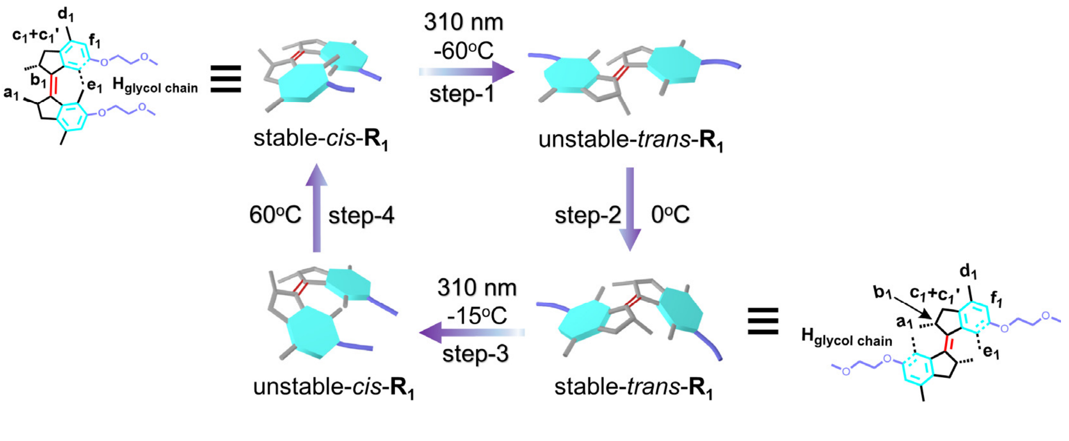

Scheme S2. Photochemical and thermal isomerization steps of the reference compound $\mathbf{R_1}$.

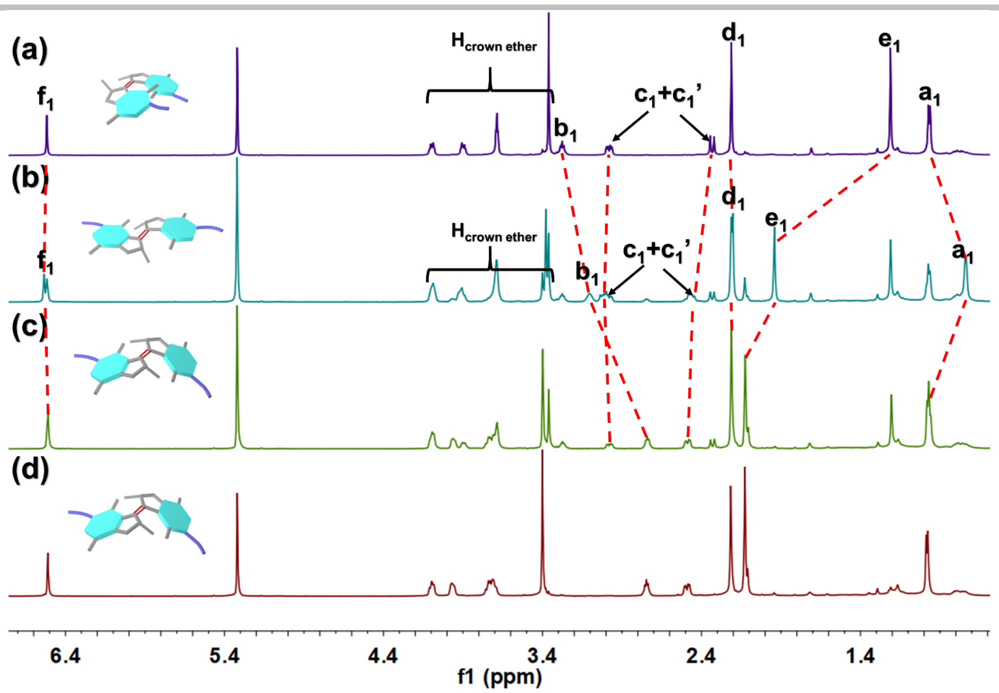

Figure S5. Partial ^{1}H NMR spectra (600 MHz, 203 K, CD_2Cl_2, 5 mM) of compounds (a) stable-*cis*-$\mathbf{R_1}$. (b) unstable-*trans*-$\mathbf{R_1}$ upon irradiation of (a) (310 nm at -60 ºC). (c) stable-*trans*-$\mathbf{R_1}$ upon heating the solution of (b) (0 ºC). (d) The comparative spectrum of synthetic pure stable-*trans*-$\mathbf{R_1}$.

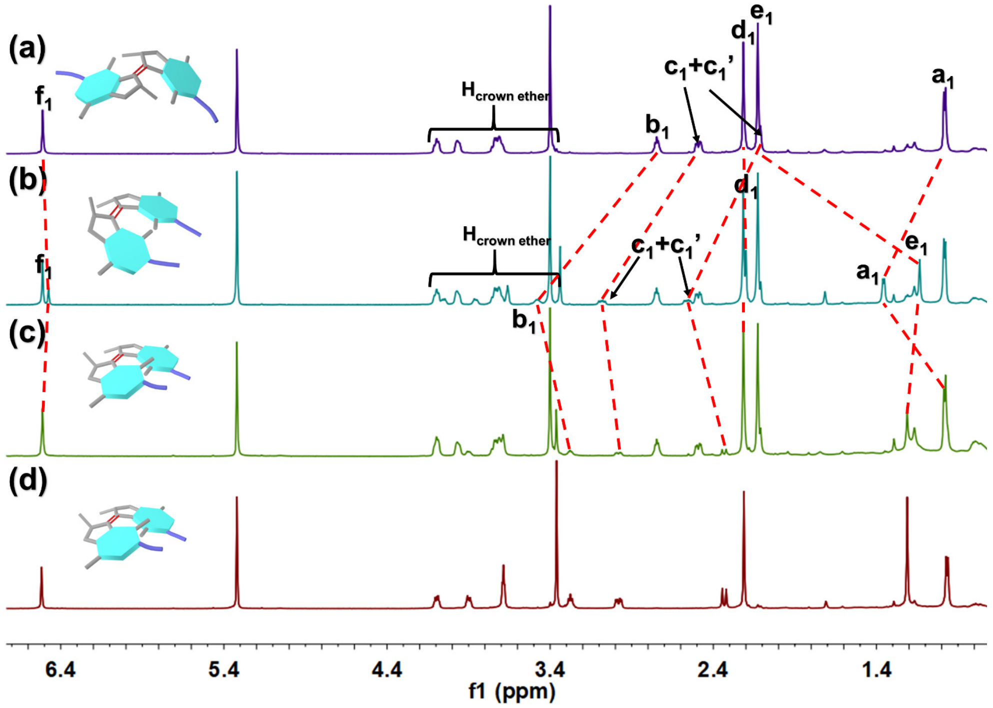

Figure S6. Partial ^{1}H NMR spectra (600 MHz, 203 K, CD_2Cl_2, 5 mM) of compounds (a) stable-*trans*-$\mathbf{R_1}$. (b) unstable-*cis*-**R1** upon irradiation of (a) (310 nm at -15 ºC). (c) stable-*cis*-$\mathbf{R_1}$ upon heating the solution of (b) (60 ºC). (d) The comparative spectrum of synthetic pure stable-*cis*-$\mathbf{R_1}$.

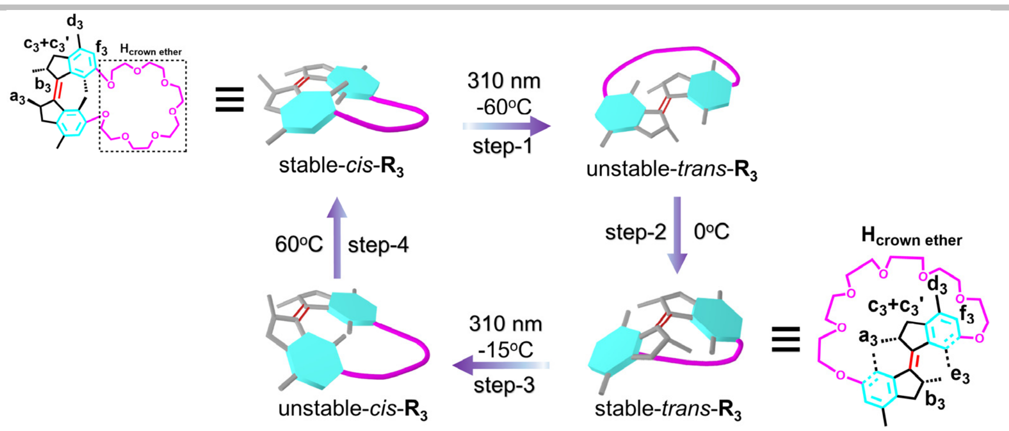

Scheme S3. Photochemical and thermal isomerization steps of the reference compound **R**$_3$.

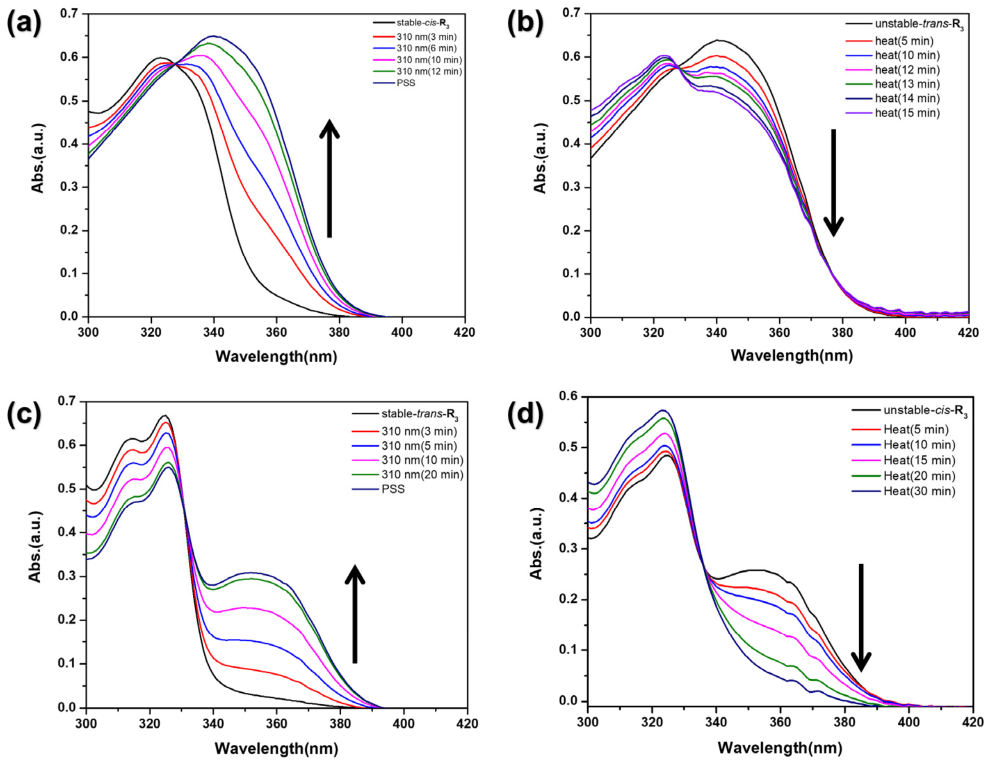

Figure S7. UV-vis absorption spectral changes in THF during the photoisomerization and THI processes, starting from: (a) stable-*cis*-**R**$_3$ to unstable-*trans*-**R**$_3$ (65 µM, 310 nm, THF, -60 °C). (b) unstable-*trans*-**R**$_3$ to stable-*trans*-**R**$_3$ (THF, 0 °C). (c) stable-*trans*-**R**$_3$ to unstable-*cis*-**R**$_3$ (65 µM, 310 nm, THF, -15 °C). (d) unstable-*cis*-**R**$_3$ to stable-*cis*-**R**$_3$ (THF, 60 °C).

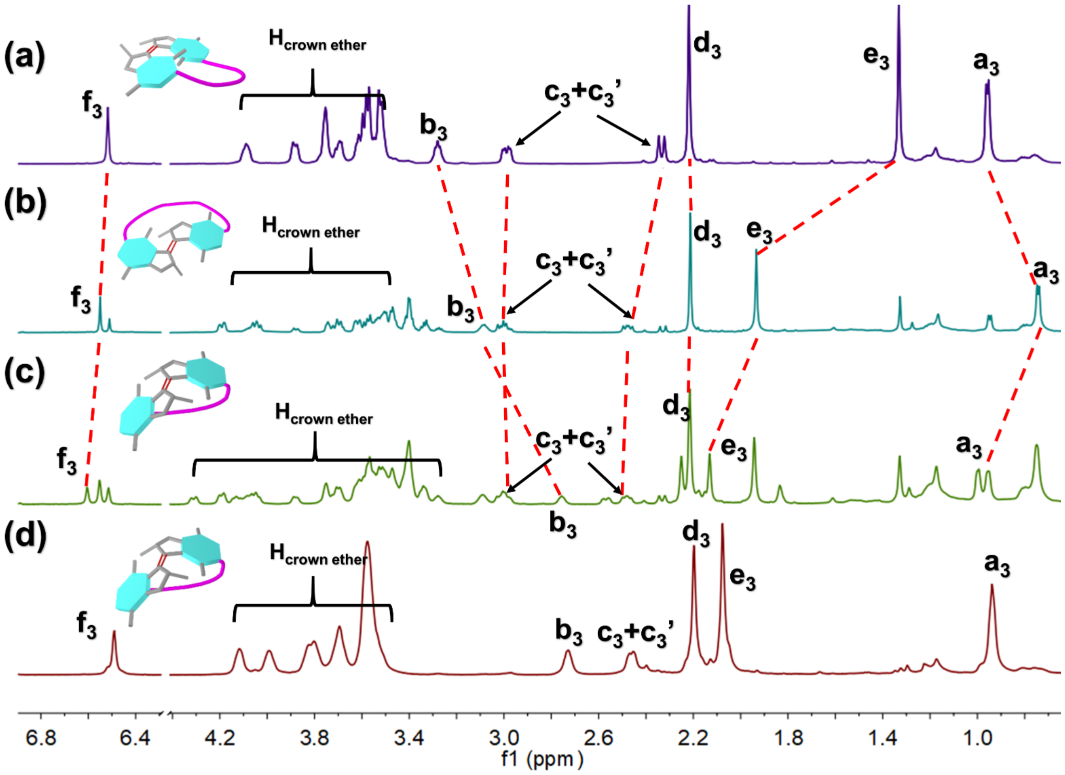

Figure S8. Partial ^{1}H NMR spectra (600 MHz, 203 K, CD_2Cl_2, 5 mM) of compounds (a) stable-*cis*-$\mathbf{R_3}$. (b) unstable-*trans*-$\mathbf{R_3}$ upon irradiation of (a) (310 nm at -60 °C). (c) stable-*trans*-$\mathbf{R_3}$ upon heating the solution of (b) (0 °C). (d) The comparative spectrum of synthetic pure stable-*trans*-$\mathbf{R_3}$. PSS yield is 73% (unstable-*trans*:stable-*cis*), lower than stable-*cis*-**3** (86%). The photochemical system with lower PSS yield resulted in the multiple overlapped proton signals of crown ether moiety, thus also leading to the slight shift of e_3 peak compared with the pure stable-*trans*-$\mathbf{R_3}$.

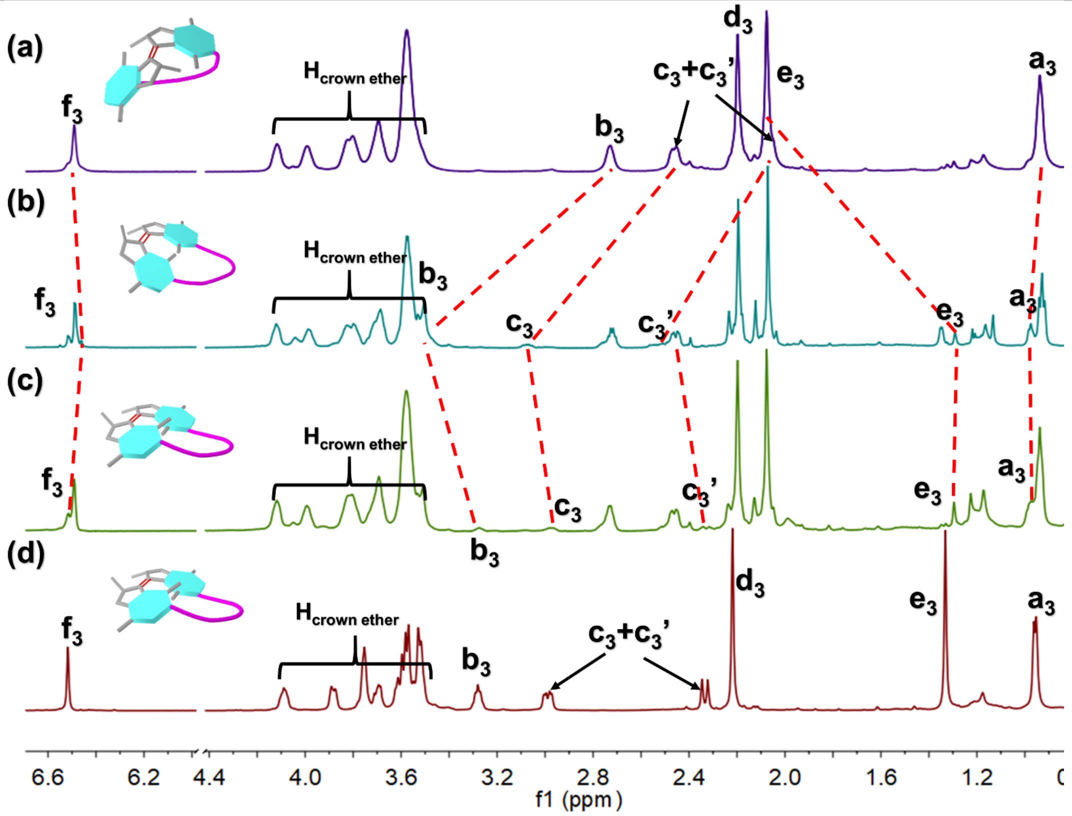

Figure S9. Partial ^{1}H NMR spectra (600 MHz, 203 K, CD_2Cl_2, 5 mM) of compounds (a) stable-*trans*-R_3. (b) unstable-*cis*-R_3 upon irradiation of (a) (310 nm at -15 °C). (c) stable-*cis*-R_3 upon heating the solution of (b) (60 °C). (d) The comparative spectrum of synthetic pure stable-*cis*-R_3. PSS yield is 27% (unstable-*cis*:stable-*trans*), lower than stable-*trans*-**3** (47%). The photochemical system with lower PSS yield resulted in the multiple overlapped proton signals of crown ether moiety, thus also leading to the slight shift of e_3 peak compared with the pure stable-*cis*-$\mathbf{R_3}$.

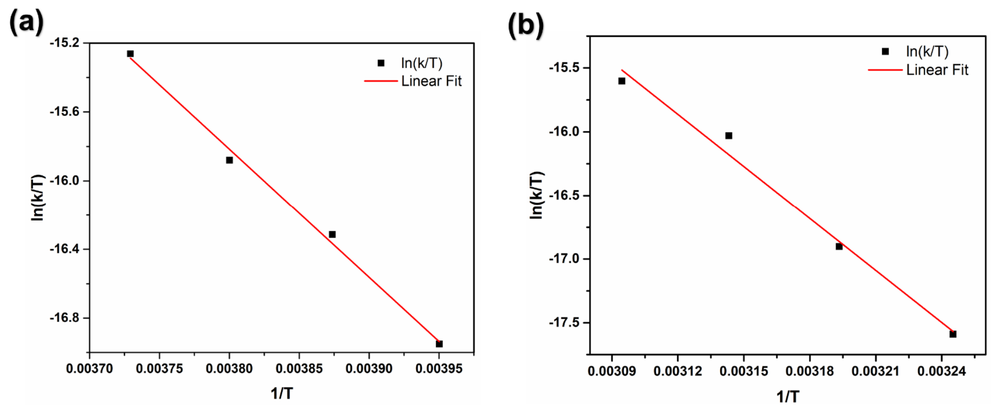

Figure S10. Kinetic studies of the thermal helix inversion steps of (a) unstable-*trans*-$\mathbf{R_3}$ to stable-*trans*-$\mathbf{R_3}$ and (b) unstable-*cis*-$\mathbf{R_3}$ to stable-*cis*-$\mathbf{R_3}$. These isomerization steps were followed by UV absorption changes at 360 nm four different temperature conditions in THF (unstable-*trans* to stable-*trans*: -20 °C, -15 °C, -10 °C and -5 °C; unstable-*cis* to stable-*cis*: 35 °C, 40 °C, 45 °C and 50 °C).

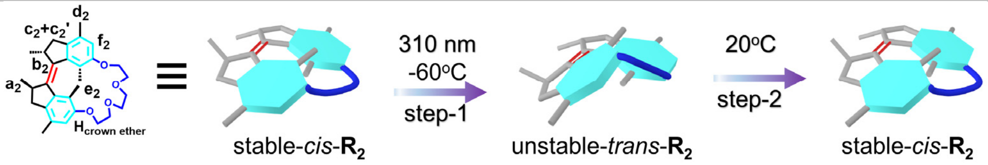

Scheme S4. Photochemical and thermal isomerization steps of the stable-*cis*-$\mathbf{R_2}$.

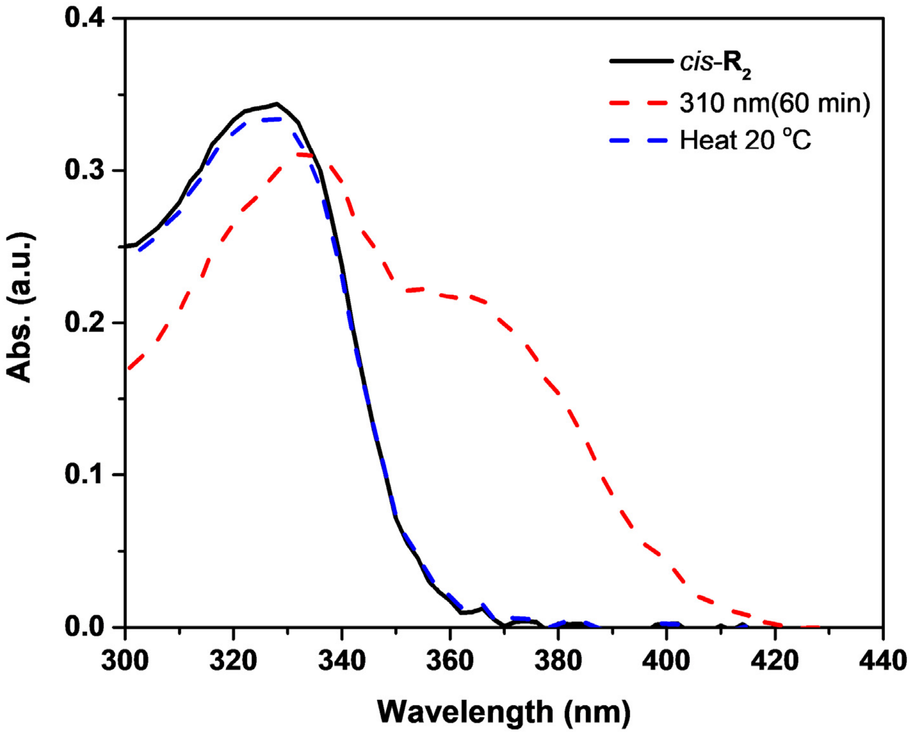

Figure S11. UV-vis absorption spectra of stable-*cis*-$\mathbf{R_2}$ (35 μM in THF) before, after irradiated (310 nm, -60 °C, 60 min), and after heat at 20 °C for 40 min.

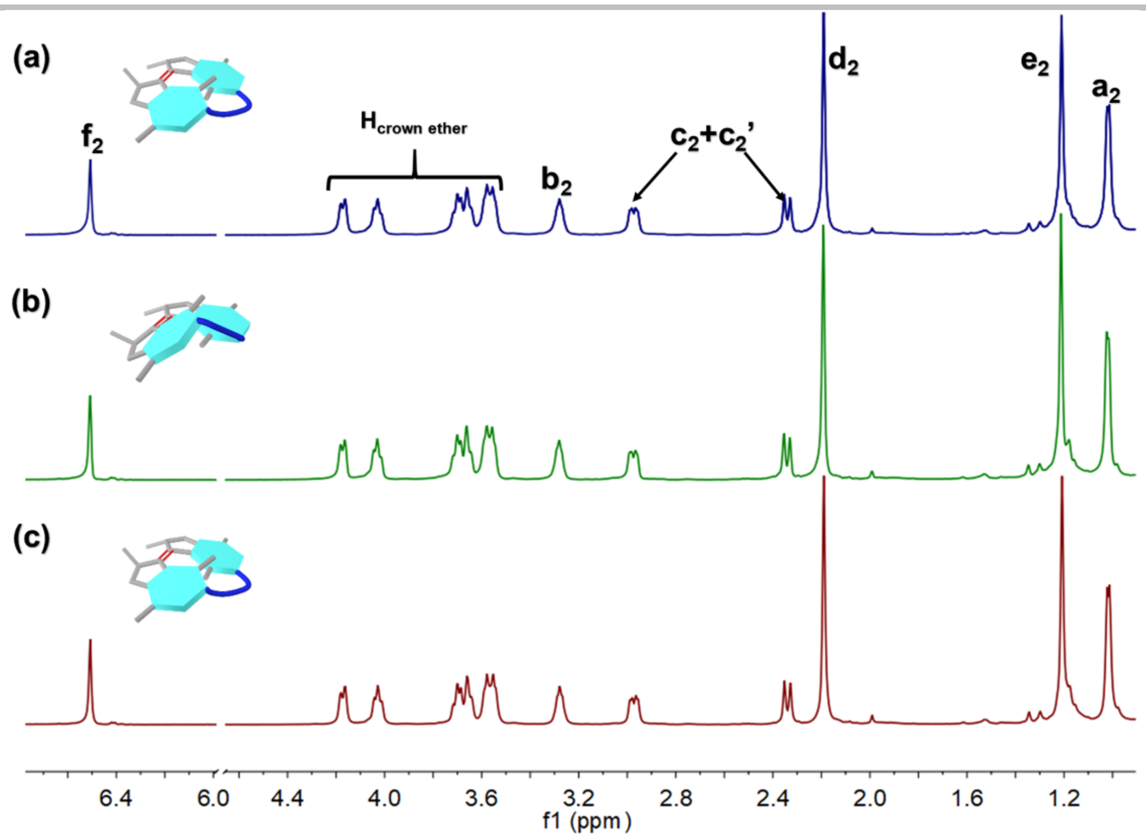

Figure S12. Partial ^{1}H NMR spectra (600 MHz, 203 K, CD_2Cl_2, 5 mM) of compounds (a) stable-*cis*-**R**$_2$. (b) Solution obtained after the irradiation of (a) (310 nm at -60 °C). (c) Upon heating the solution of (b) (20 °C).

4. Single Crystal X-ray Analysis of *cis*-R$_2$

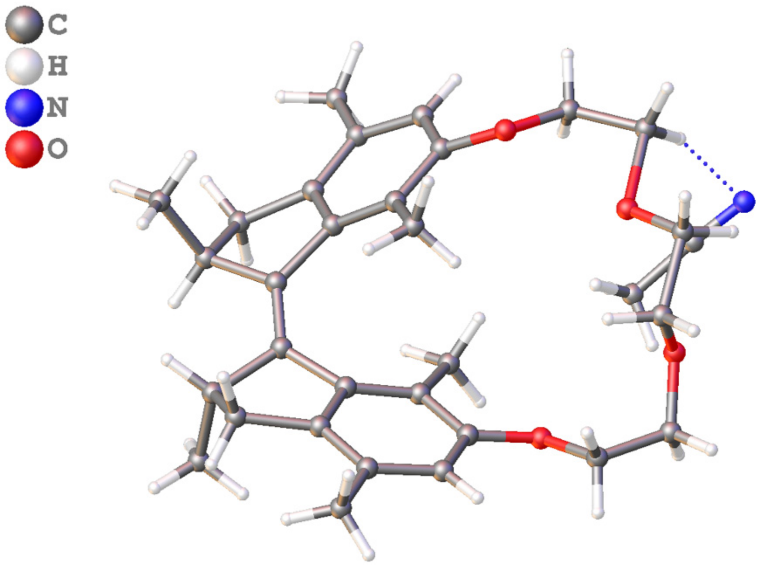

Figure S13. Single crystal structure of (*M*, *M*)-(*S*, *S*)-*cis*-**R**$_2$ crystallized from CH_3CN.

Table S2. Crystal data and structure refinement for *cis*-$\mathbf{R_2}$•CH_3CN.

Identification code	*cis*-$\mathbf{R_2}$•CH_3CN
Empirical formula	C32 H41 N O4
Formula weight	503.66
Temperature	169.98 K
Wavelength	1.34139 Å
Crystal system	Monoclinic
Space group	P 1 21/c 1
Unit cell dimensions	a = 6.1125(8) Å a= 90°.<br>b = 23.433(3) Å b= 98.104(6)°<br>c = 19.546(2) Å g = 90°.
Volume	2771.7(6) Å^3
Z	4
Density (calculated)	1.207 Mg/m^3
Absorption coefficient	0.398 mm^{-1}
F(000)	1088
Crystal size	0.12 x 0.08 x 0.08 mm^3
Theta range for data collection	3.837 to 54.878°.
Index ranges	-5<=h<=7, -28<=k<=27, -23<=l<=19
Reflections collected	26449
Independent reflections	5255 [R(int) = 0.0656]
Completeness to theta = 53.594°	99.7 %
Absorption correction	Semi-empirical from equivalents
Max. and min. transmission	0.7508 and 0.4537
Refinement method	Full-matrix least-squares on F^2
Data / restraints / parameters	5255 / 2 / 377
Goodness-of-fit on F^2	1.070
Final R indices [I>2sigma(I)]	R1 = 0.0802, wR2 = 0.2228
R indices (all data)	R1 = 0.0981, wR2 = 0.2385
Extinction coefficient	n/a
Largest diff. peak and hole	0.266 and -0.468 e.Å^{-3}

5. NMR Studies on Host-Guest Combinations

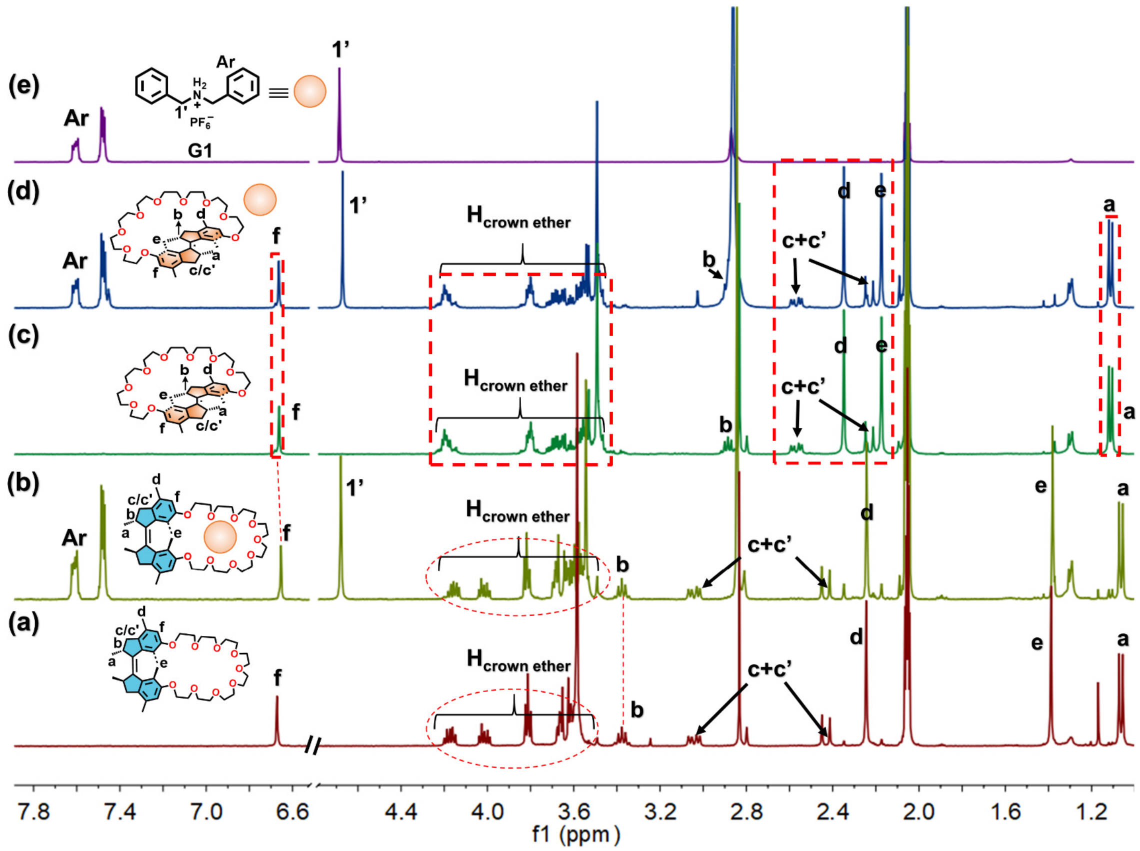

Figure S14. Partial ^{1}H NMR spectra (400 MHz, 293 K, acetone-d_6) of (a) macrocycle stable-*cis*-**3**. (b) [stable-*cis*-**3** ⊃ **G1**]. (c) stable-*trans*-**3**. (d) stable-*trans*-**3** mixing with guest (1:1) and (e) guest **G1**.

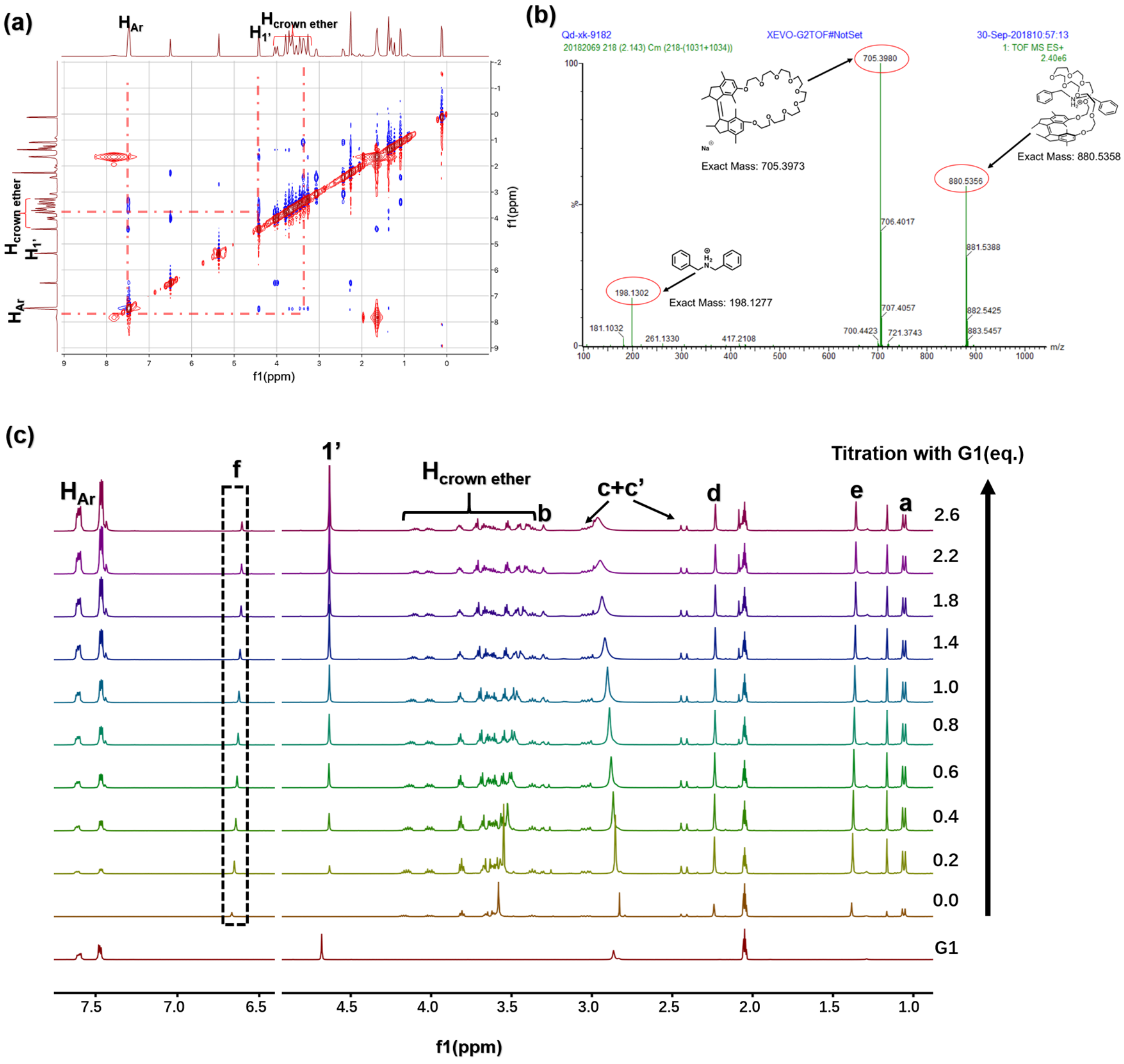

Figure S15. (a) Partial 2D NOESY spectra (400 MHz, 293 K, CD_2Cl_2) of [stable-*cis*-**3** ⊃ **G1**]. (b) ESI spectra of [stable-*cis*-**3** ⊃ **G1**]. (c) Partial ^{1}H NMR spectra of stable-*cis*-**3** (5 mM, 400 MHz, 293 K, acetone-d_6) upon the stepwise addition of **G1**.

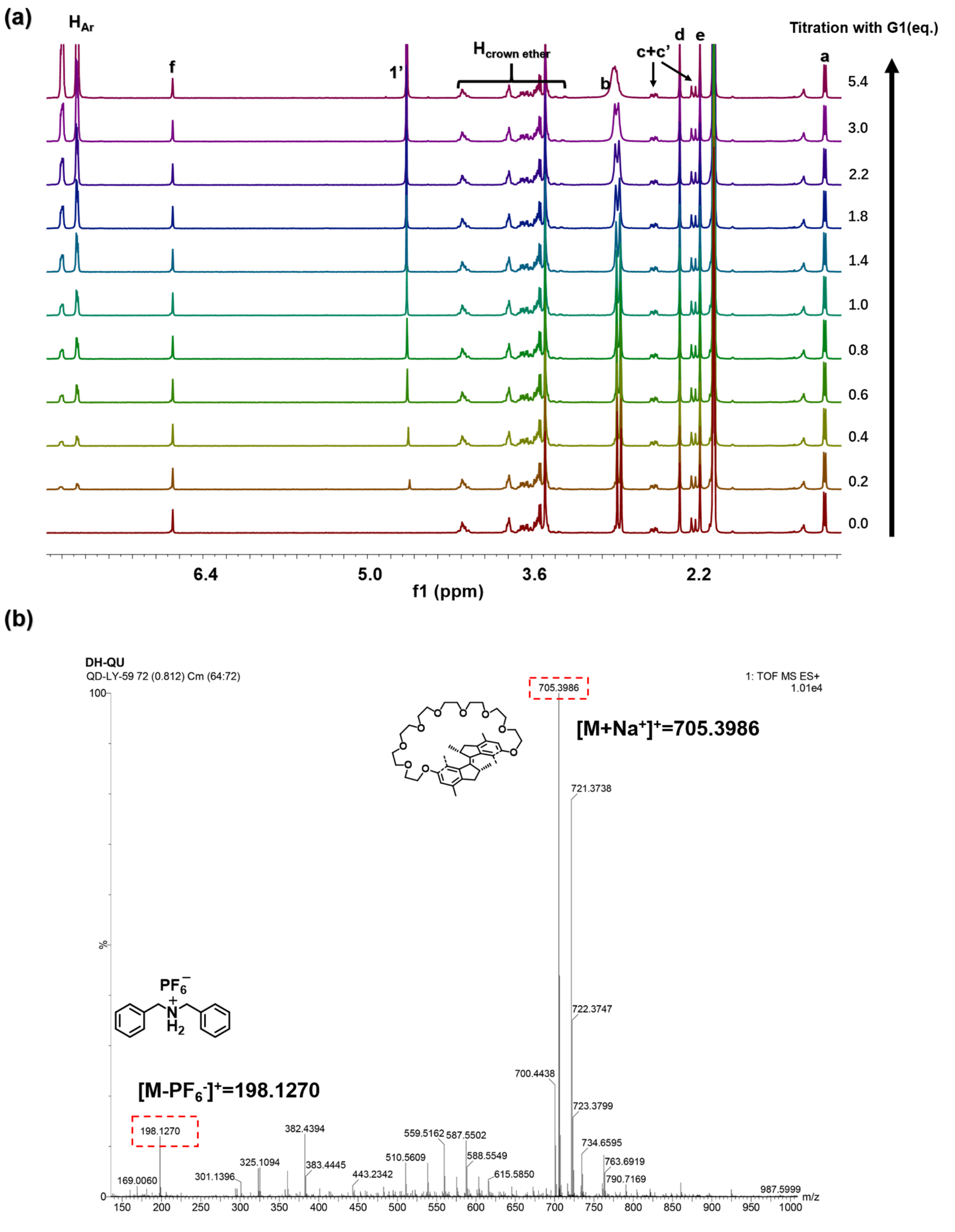

Figure S16. (a) Partial ^{1}H NMR spectra of stable-*trans*-**3** (5 mM, 400 MHz, 293 K, acetone-d_6) upon the stepwise addition of **G1** and (b) ESI spectra of [stable-*trans*-**3** mixing with **G1**].

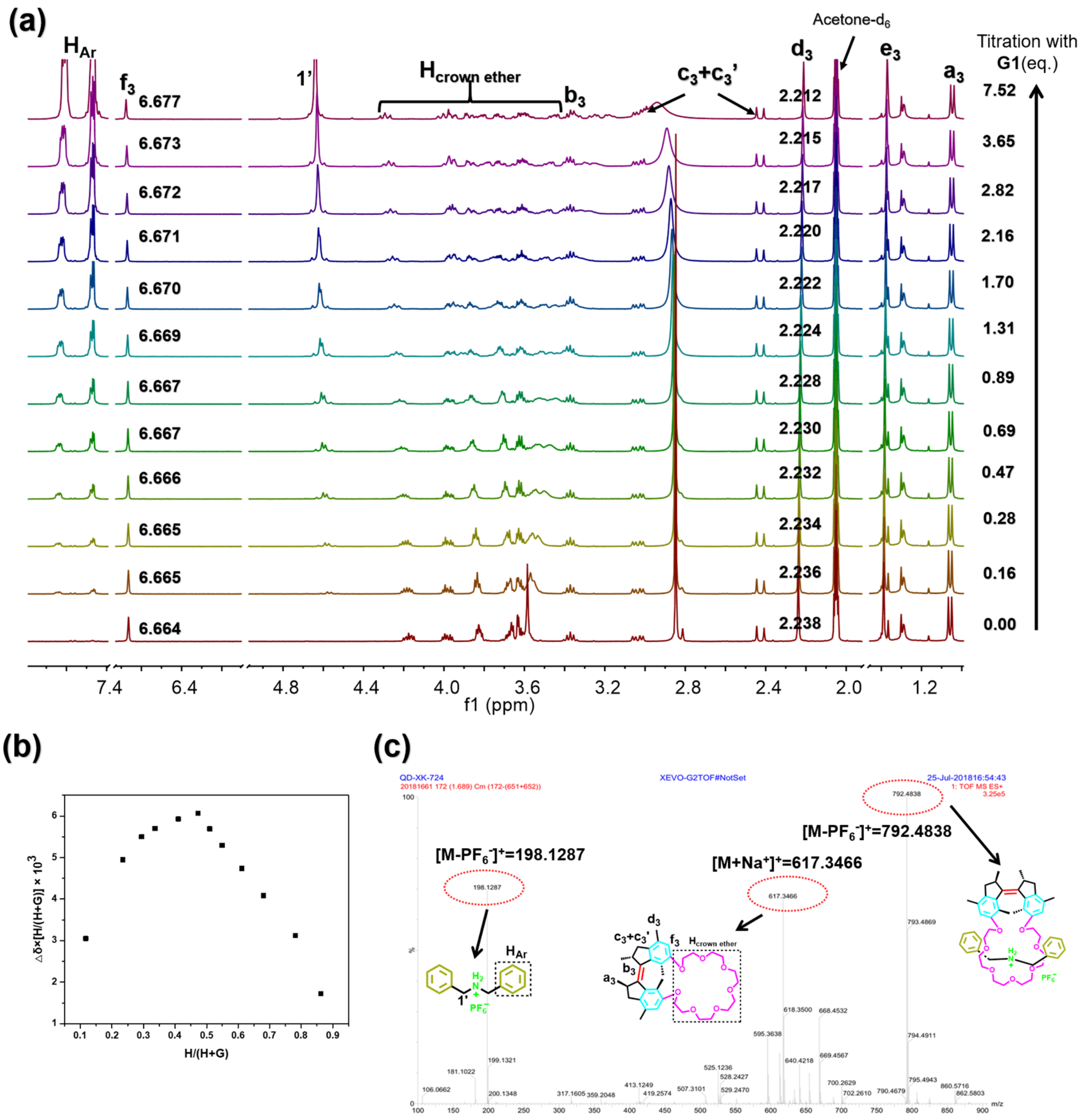

Figure S17. (a) Partial ^{1}H NMR spectra of stable-*cis*-**R$_3$** (5 mM, 400 MHz, 293 K, acetone-*d$_6$*) upon the stepwise addition of **G1**. (b) Job's plot based on the proton shift of H_{d3} in acetone-*d$_6$* [stable-*cis*-**R$_3$** ⊃ **G1**]. (c) ESI-mass spectrum of [stable-*cis*-**R$_3$** ⊃ **G1**] (792.4838).

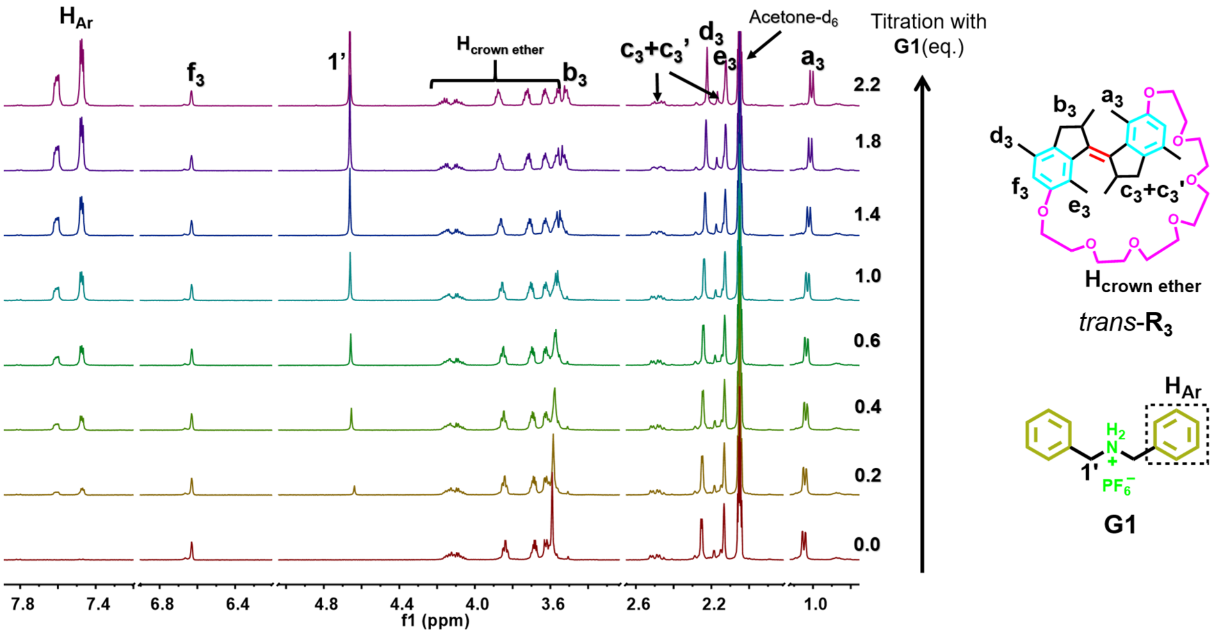

Figure S18. Partial ^{1}H NMR spectra of stable-*trans*-**R$_3$** (5 mM, 400 MHz, 293 K, acetone-*d$_6$*) upon the stepwise addition of **G1**.

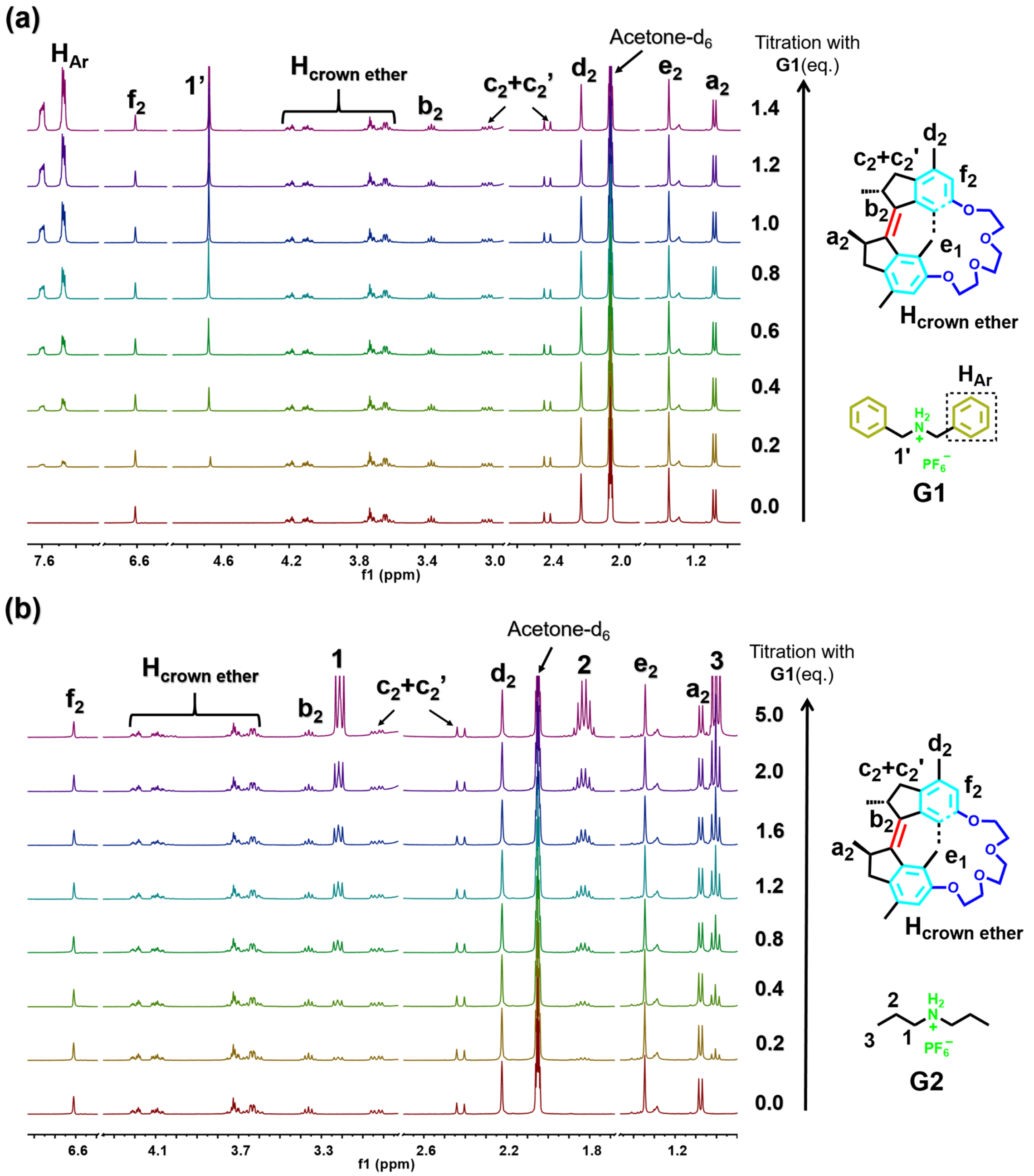

Figure S19. (a) Partial ^{1}H NMR spectra of stable-*cis*-$\mathbf{R_2}$ (5 mM, 400 MHz, 293 K, acetone-d_6) upon the stepwise addition of **G1**. (b) Partial ^{1}H NMR spectra of stable-*cis*-$\mathbf{R_2}$ (5 mM, 400 MHz, 293 K, acetone-d_6) upon the stepwise addition of **G2**.

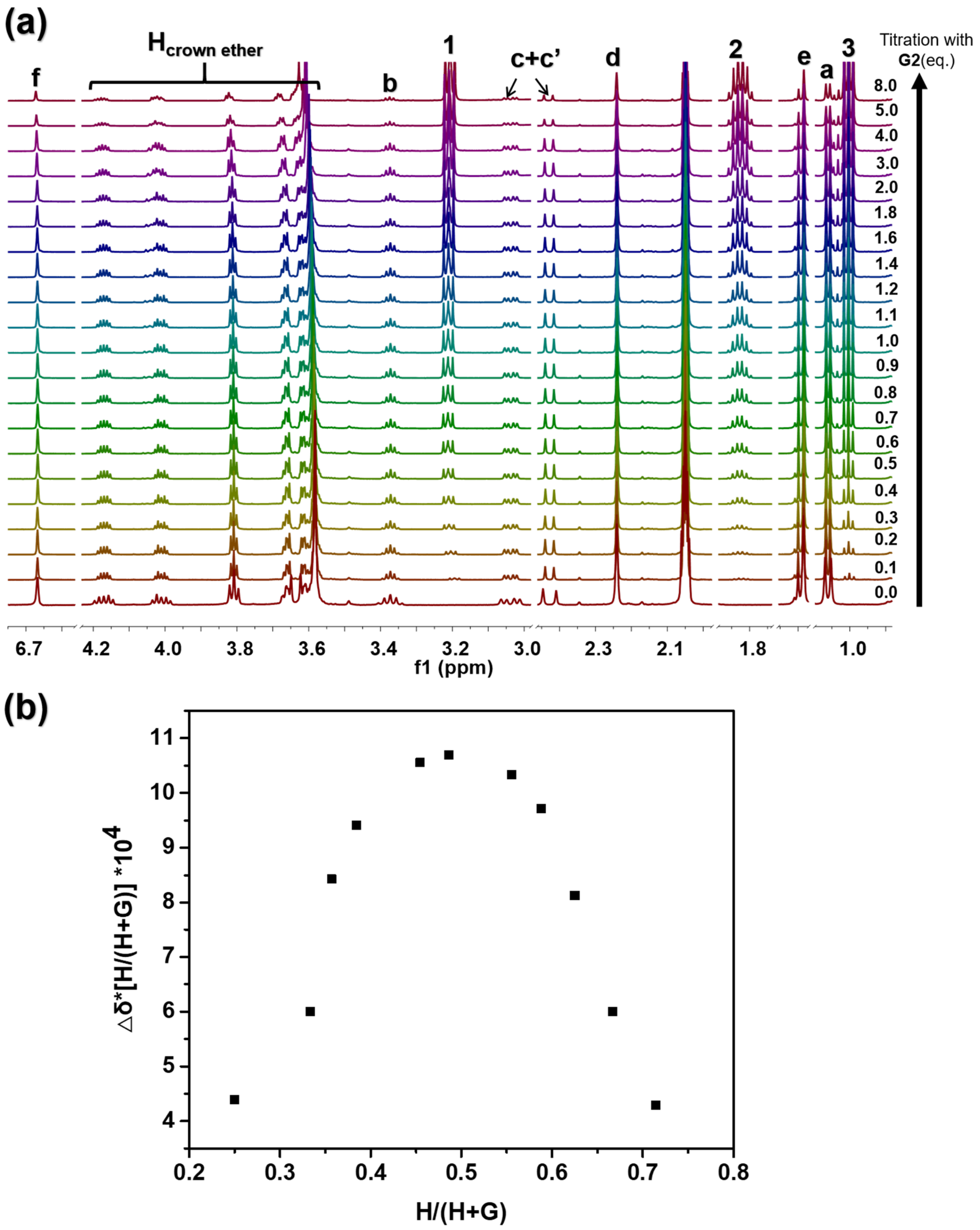

Figure S20. (a) Partial ^{1}H NMR spectra of stable-*cis*-**3** (5 mM, 400 MHz, 293 K, acetone-d_6) upon the stepwise addition of **G2**. (b) Job's plot based on the proton shift of H_f.

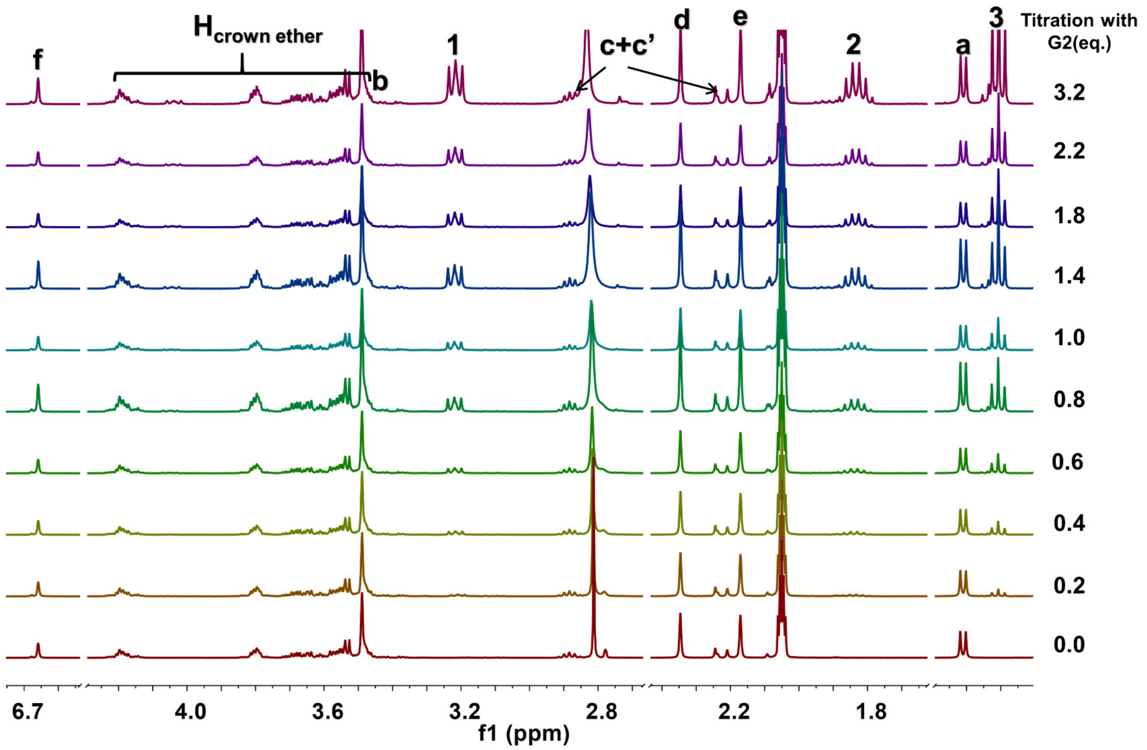

Figure S21. Partial ^{1}H NMR spectra of stable-*trans*-**3** (5 mM, 400 MHz, 293 K, acetone-d_6) upon the stepwise addition of **G2**.

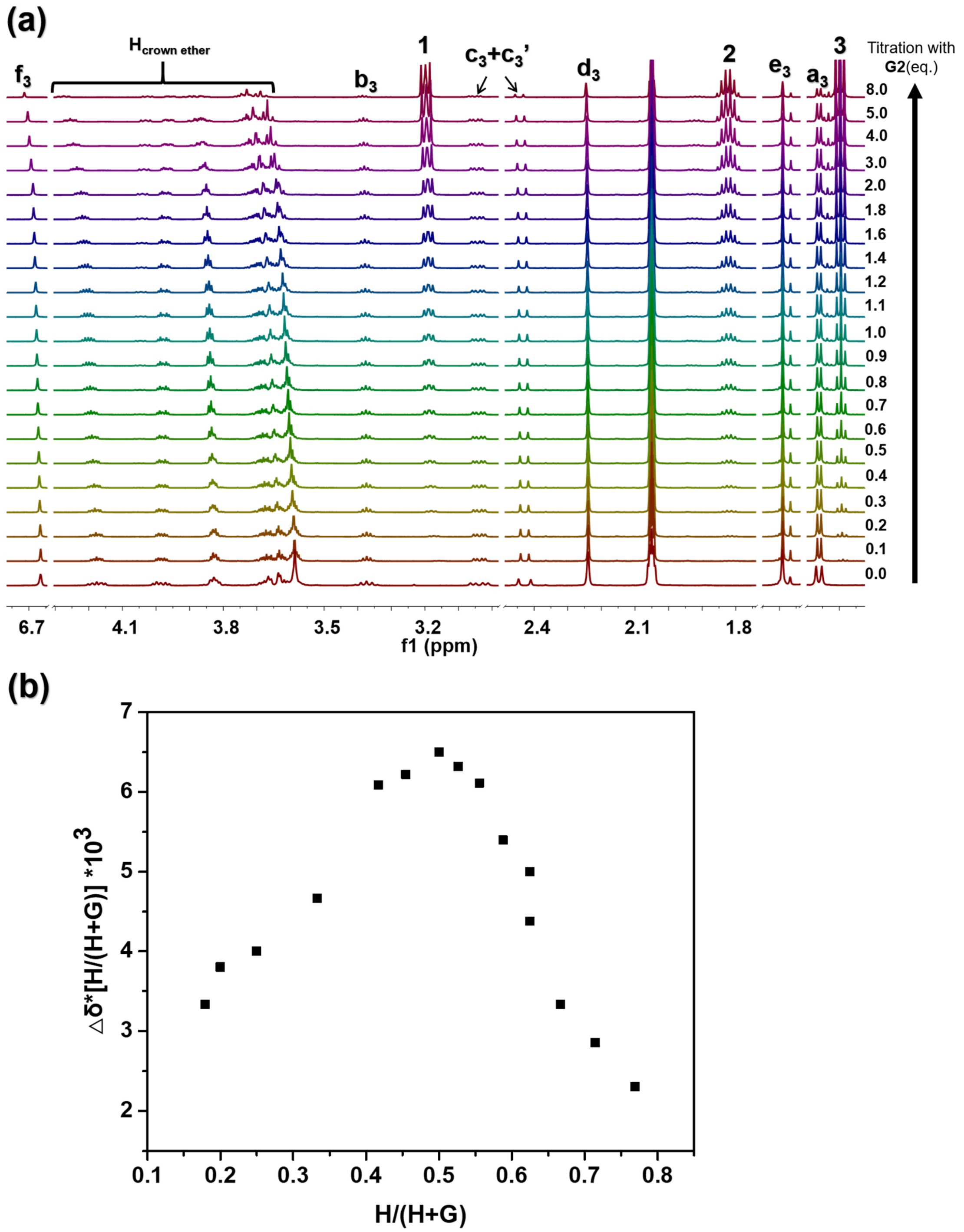

Figure S22. (a) Partial ^{1}H NMR spectra of stable-*cis*-**R$_3$** (5 mM, 400 MHz, 293 K, acetone-d_6) upon the stepwise addition of **G2**. (b) Job's plot based on the proton shift of H_{f3}.

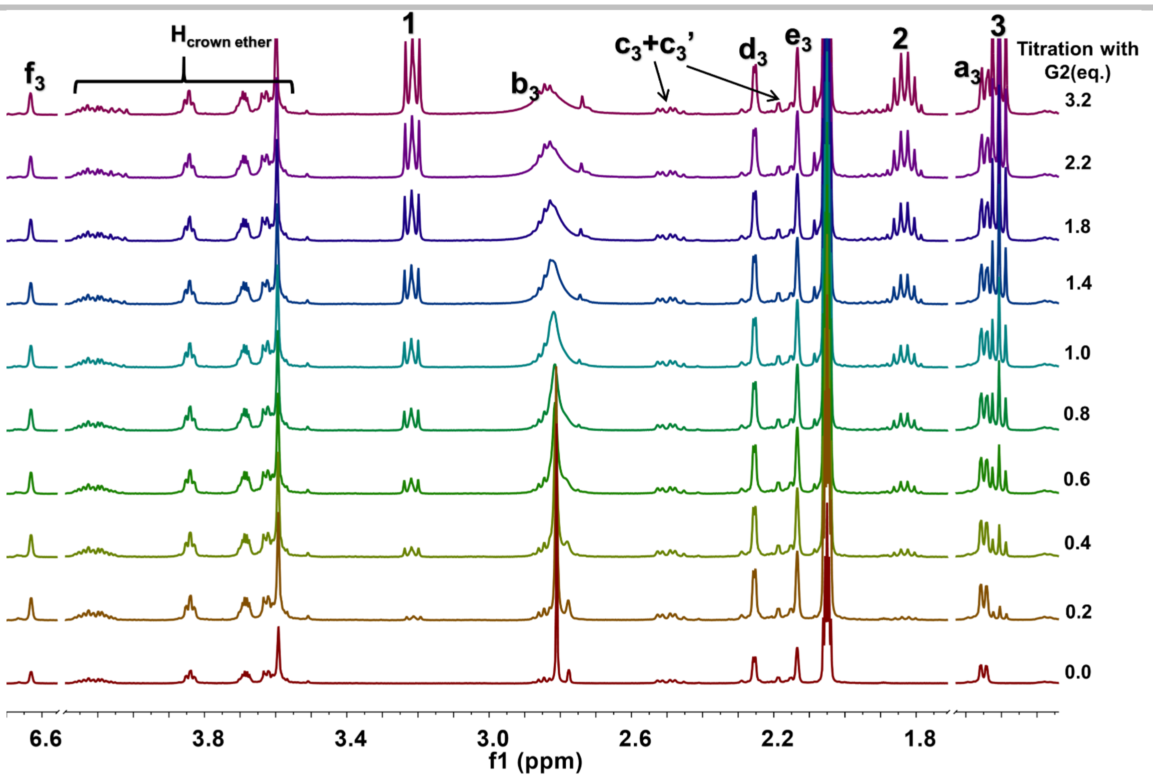

Figure S23. Partial ^{1}H NMR spectra of stable-*trans*-**R**$_3$ (5 mM, 400 MHz, 293 K, acetone-d_6) upon the stepwise addition of **G2**.

6. NMR and UV-vis Studies on Guest Release/Capture Procedures

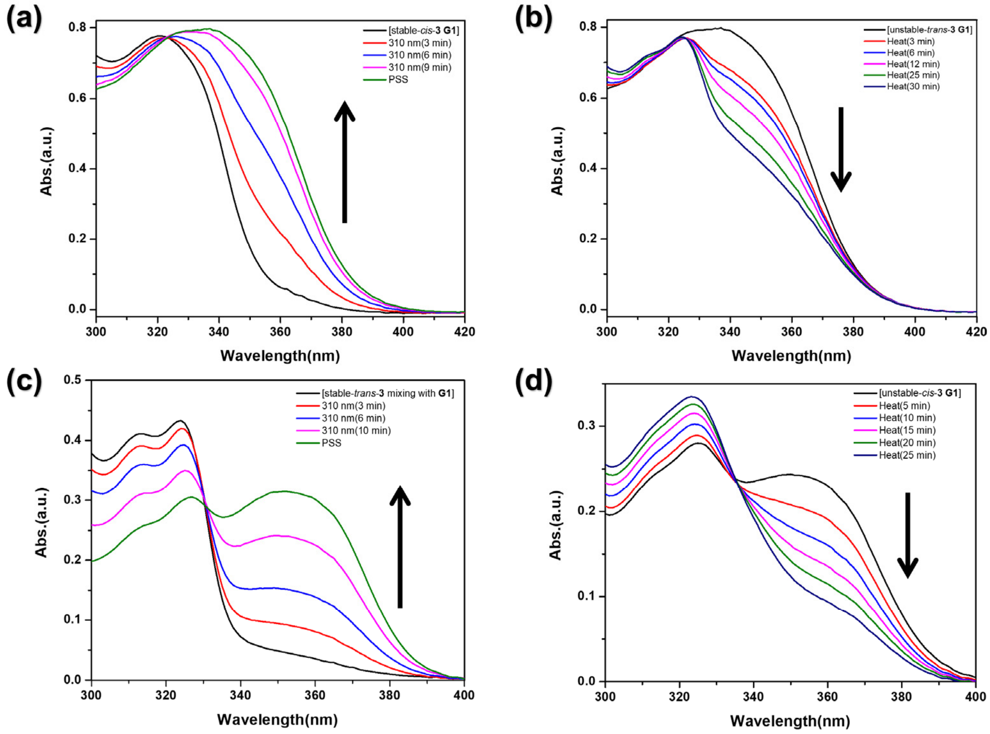

Figure S24. UV-vis absorption spectral changes in THF during the photoisomerization and THI processes, starting from: (a) [stable-*cis*-**3** ⊃ **G1**] to [unstable-*trans*-**3** mixing with **G1**] (80 μM, 310 nm, -60 °C). (b) [unstable-*trans*-**3** mixing with **G1**] to [stable-*trans*-**3** mixing with **G1**] (0 °C). (c) [stable-*trans*-**3** mixing with **G1**] to [unstable-*cis*-**3** ⊃ **G1**] (45 μM, 310 nm, -15 °C). (d) [unstable-*cis*-**3** ⊃ **G1**] to [stable-*cis*-**3** ⊃ **G1**] (60 °C).

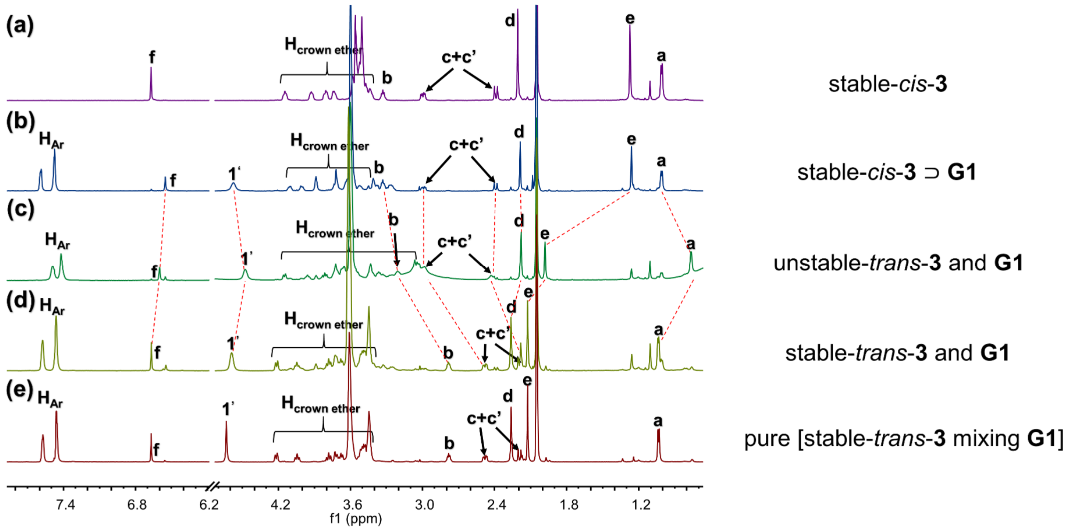

Figure S25. Partial ^{1}H NMR spectra (600 MHz, 203 K, acetone-d_6, 5 mM) of compounds (a) pure stable-*cis*-**3**. (b) [stable-*cis*-**3** ⊃ **G1**]. (c) Irradiated the solution of (b) at 310 nm at -60 ºC. (d) Heating the solution of (c) at 0 ºC for 12 h and (e) control group of stable-*trans*-**3** upon mixing with **G1**.

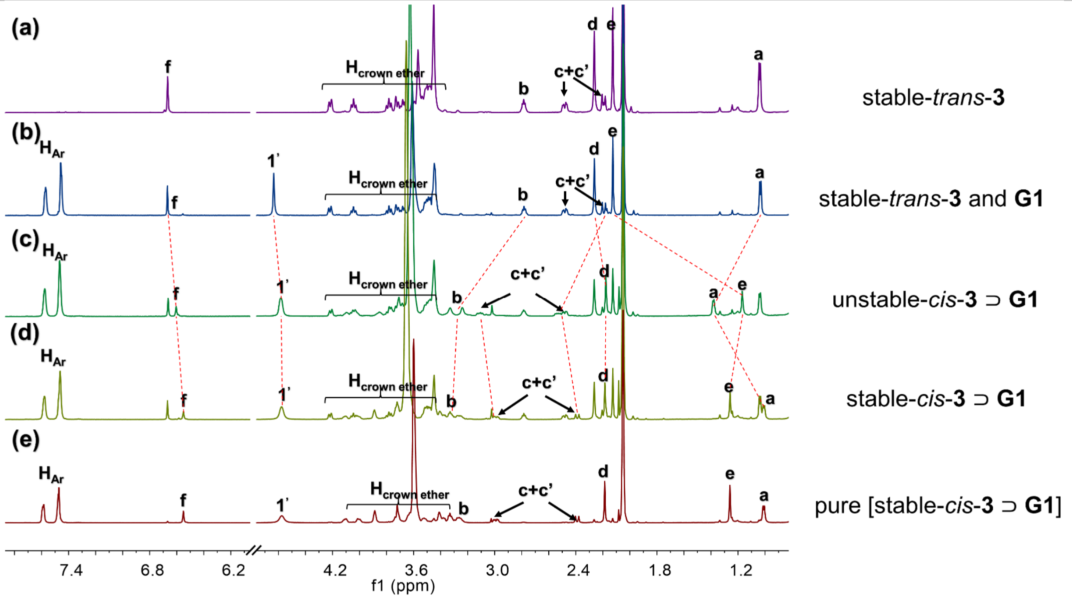

Figure S26. Partial ^{1}H NMR spectra (600 MHz, 203 K, acetone-d_6, 5 mM) of compounds (a) pure stable-*trans*-**3**. (b) stable-*trans*-**3** upon mixing with **G1** (1:1). (c) Irradiated solution of (b) at 310 nm at -15 °C. (d) Heated solution of (c) at 60 °C for 12 h and (e) control [stable-*cis*-**3** ⊃ **G1**].

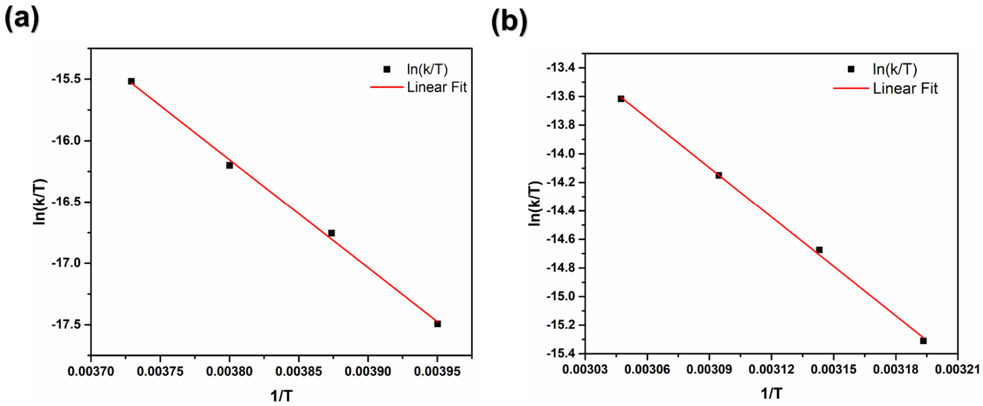

Figure S27. Kinetic studies of the thermal helix inversion steps of (a) unstable-*trans*-**3** mixing with **G1** to stable-*trans*-**3** mixing with **G1** and (b) unstable-*cis*-**3** ⊃ **G1** to stable-*cis*-**3** ⊃ **G1**. These isomerization steps were followed by UV absorption changes at 360 nm four different temperatures in THF(unstable-*trans* to stable-*trans*: -20 °C, -15 °C, -10 °C and -5 °C; unstable-*cis* to stable-*cis*: 40 °C, 45 °C, 50 °C and 55 °C).

7. Chirality Separation for Enantiomers *cis*-3 and Enantiomers *trans*-3

7.1 Chiral HPLC of racemic compound *cis*-3

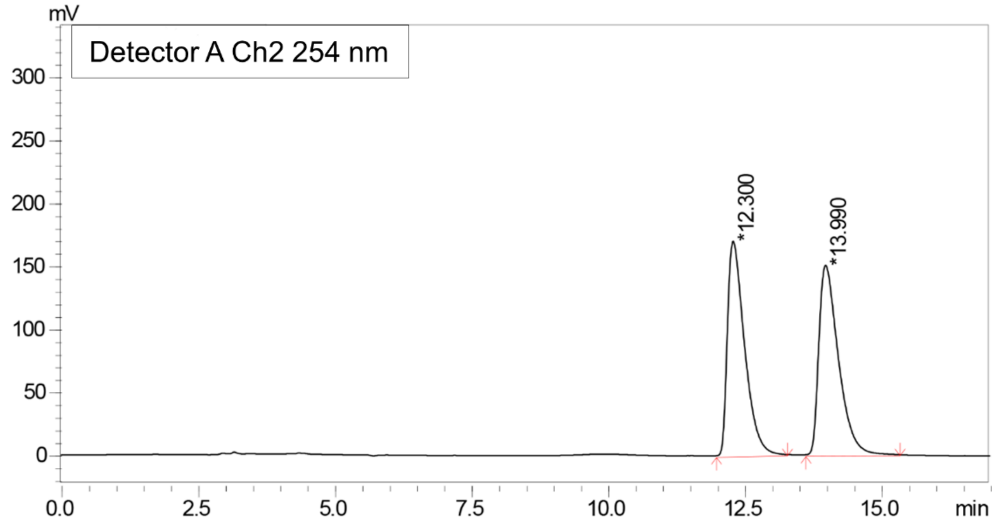

Figure S28. HPLC chromatogram (CHIRALPAK AD-H(ADH0CE-XG136)), 0.46 cm I.D. × 25 cm L) of racemic compound *cis*-**3**, eluted with Hexane/IPA=95/5 (V/V), at a flow rate of 1.0 mL min^{-1} detected at 254 nm.

Table S3. Peak table.

Peak	Ret. Time	Area	Area%	T. Plate#	Tailing F.	Resolution
1	12.300	3688423	49.719	7488	1.835	--
2	13.990	3730169	50.281	7540	1.777	2.787

7.2 Chiral HPLC of (*M*, *M*)-(*S*, *S*)-*cis*-3

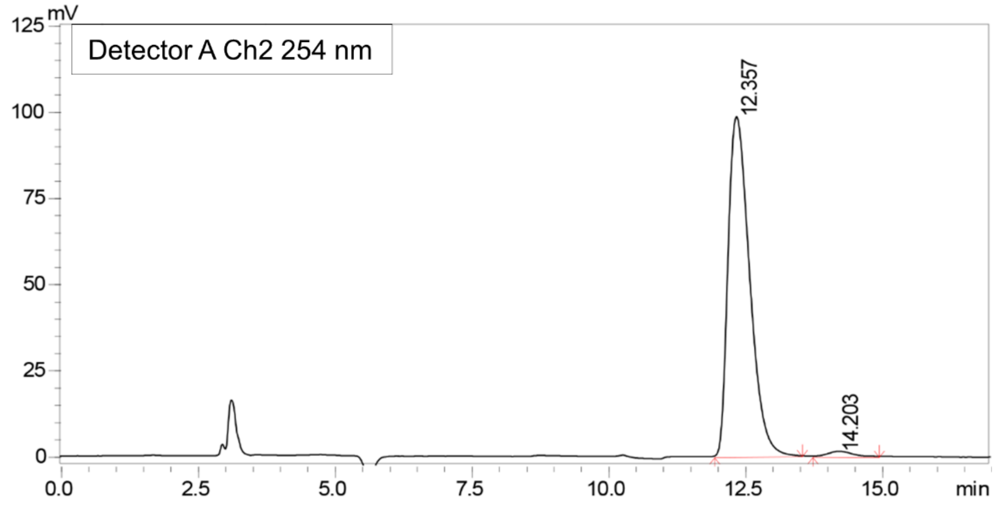

Figure S29. HPLC chromatogram (CHIRALPAK AD-H (ADH0CE-XG136)), 0.46 cm I.D. × 25 cm L) of (*M*, *M*)-(*S*, *S*)-*cis*-**3**, eluted with Hexane/IPA=95/5 (V/V), at a flow rate of 1.0 mL min^{-1} detected at 254 nm.

Table S4. Peak table.

Peak	Ret. Time	Area	Area%	T. Plate#	Tailing F.	Resolution
1	12.357	2692432	98.511	4778	1.504	--
2	14.203	40390	1.478	6085	1.240	2.559

7.3 Chiral HPLC of (*P*, *P*)-(*R*, *R*)-*cis*-3

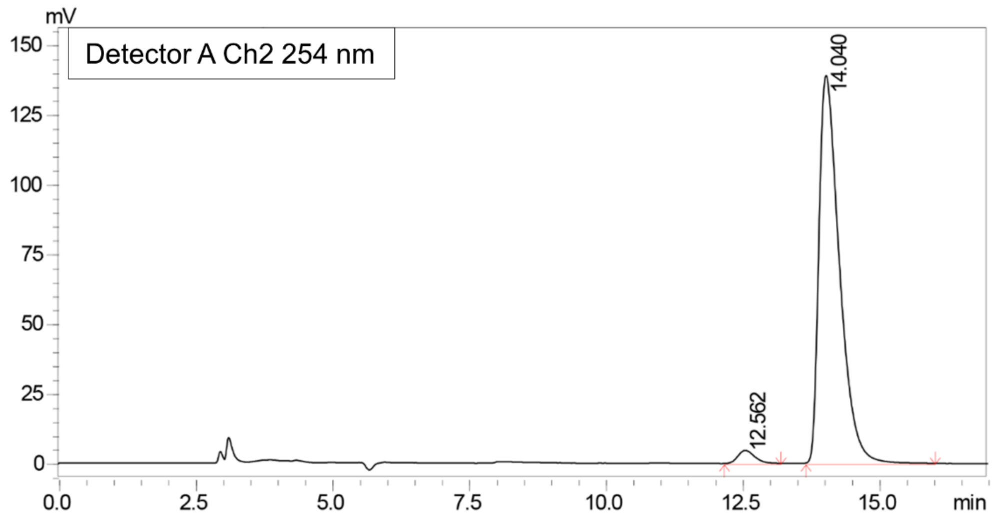

Figure S30. HPLC chromatogram (CHIRALPAK AD-H (ADH0CE-XG136)), 0.46 cm I.D. × 25 cm L) of (*P*, *P*)-(*R*, *R*)-*cis*-**3**, eluted with Hexane/IPA=95/5 (V/V), at a flow rate of 1.0 mL min^{-1} detected at 254 nm.

Table S5. Peak table.

Peak	Ret. Time	Area	Area%	T. Plate#	Tailing F.	Resolution
1	12.562	97968	2.641	8144	1.228	--
2	14.040	3612086	97.359	6915	1.745	2.399

7.4 CD Spectra of (*M*, *M*)-(*S*, *S*)-*cis*-3 and (*P*, *P*)-(*R*, *R*)-*cis*-3

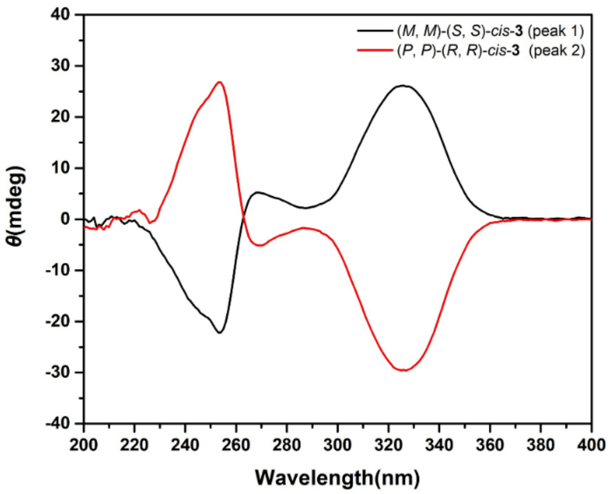

Figure S31. CD spectra (35 μM, CH_2Cl_2, 298K) of (*M*, *M*)-(*S*, *S*)-*cis*-**3** (peak 1) and (*P*, *P*)-(*R*, *R*)-*cis*-**3** (peak 2).

7.5 Chiral HPLC of racemic compound *trans*-3

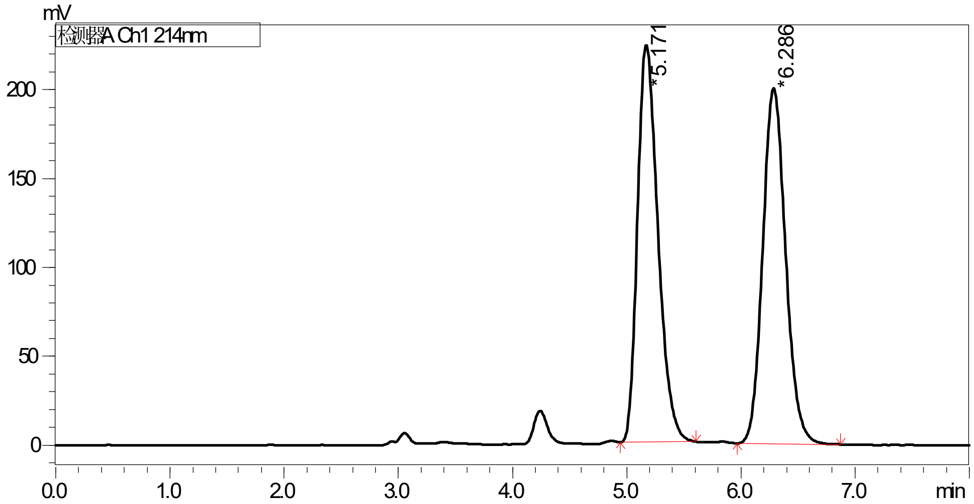

Figure S32. HPLC chromatogram (CHIRALPAK AD-H (ADH0CE-VD097)), 0.46 cm I.D. × 25 cm L) of racemic compound *trans*-**3**, eluted with Hexane/EtOH=80/20 (V/V), at a flow rate of 1.0 mL min^{-1} detected at 214 nm.

Table S6. Peak table.

Peak	Ret. Time	Area	Area%	T. Plate#	Tailing F.	Resolution
1	5.171	2782339	49.968	3820	1.313	--
2	6.286	2785917	50.032	4588	1.126	3.160

7.6 Chiral HPLC of (*M*, *M*)-(*S*, *S*)-*trans*-3

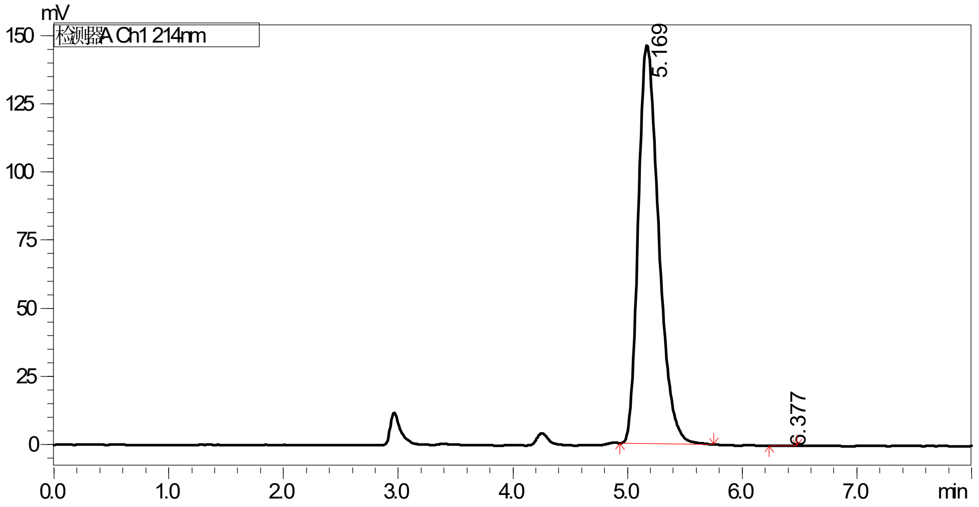

Figure S33. HPLC chromatogram (CHIRALPAK AD-H (ADH0CE-VD097)), 0.46 cm I.D. × 25 cm L) of (*M*, *M*)-(*S*, *S*)-*trans*-**3**, eluted with Hexane/EtOH=80/20 (V/V), at a flow rate of 1.0 mL min^{-1} detected at 214 nm.

Table S7. Peak table.

Peak	Ret. Time	Area	Area%	T. Plate#	Tailing F.	Resolution
1	5.169	1816653	99.970	3873	1.326	--
2	6.377	537	0.030	26681	0.949	4.946

7.7 Chiral HPLC of (*P*, *P*)-(*R*, *R*)-*trans*-3

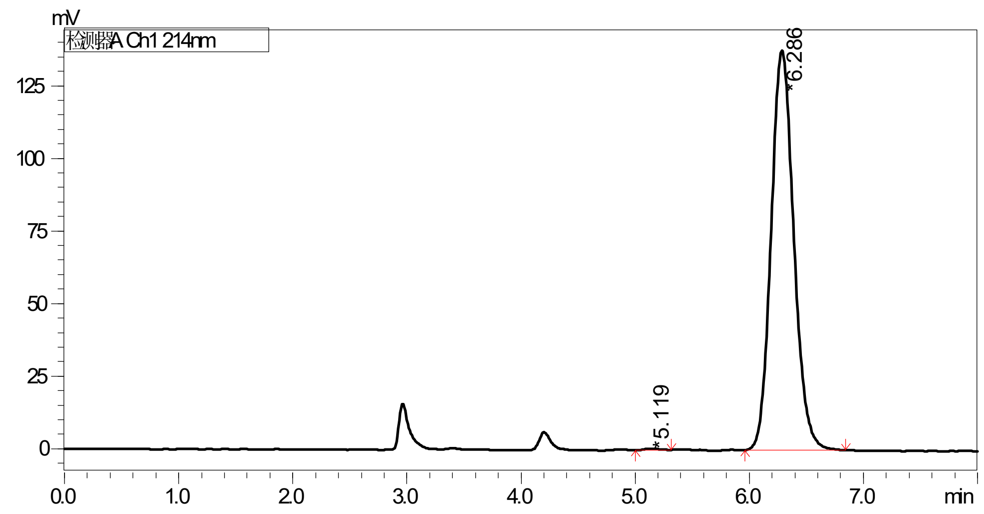

Figure S34. HPLC chromatogram (CHIRALPAK AD-H (ADH0CE-VD097)), 0.46 cm I.D. × 25 cm L) of (*P*, *P*)-(*R*, R)-*trans*-**3**, eluted with Hexane/EtOH = 80/20 (V/V), at a flow rate of 1.0 mL min^{-1} detected at 214 nm.

Table S8. Peak table.

Peak	Ret. Time	Area	Area%	T. Plate#	Tailing F.	Resolution
1	5.119	4476	0.235	5902	1.376	--
2	6.286	1898036	99.765	4697	1.122	3.684

7.8 CD Spectra of (*M*, *M*)-(*S*, *S*)-*trans*-3 and (*P*, *P*)-(*R*, *R*)-*trans*-3

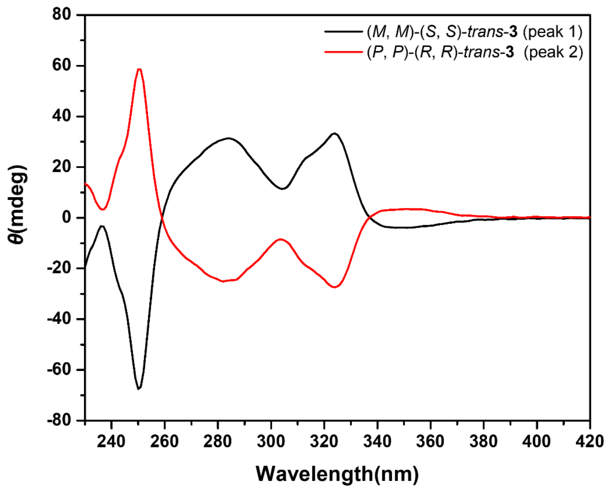

Figure S35. CD spectra (75 µM, CH_2Cl_2, 298K) of (*M*, *M*)-(*S*, *S*)-*trans*-**3** (peak 1) and (*P*, *P*)-(*R*, *R*)-*trans*-**3** (peak 2).

8. Chiral Recognition, Binding Studies

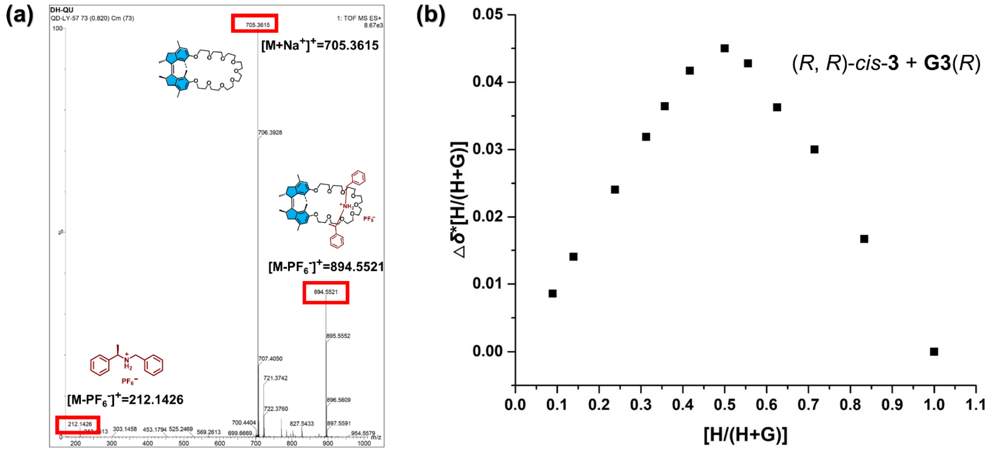

Figure S36. (a) ESI-mass spectrum of (*P*, *P*)-(*R*, *R*)-*cis*-**3** ⊃ **G3**(*R*) (894.5521). (b) Job's plot based on the proton shift of H_f in CD_2Cl_2.

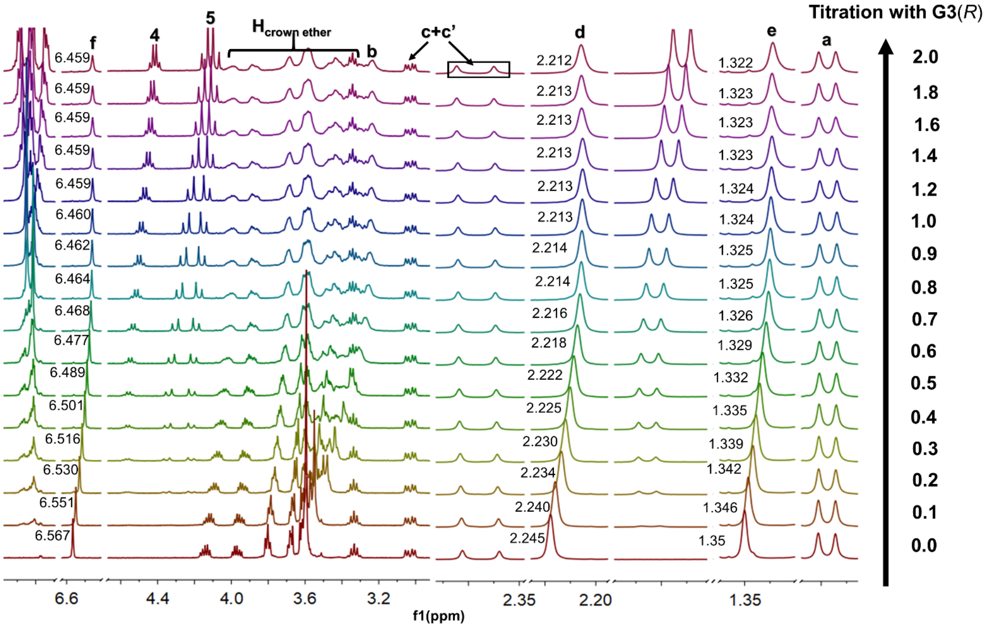

Figure S37. Partial ^{1}H NMR spectral of (*P*, *P*)-(*R*, *R*)-*cis*-**3** (5 mM, 400 MHz, 293 K, CD_2Cl_2) upon the stepwise addition of **G3**(*R*).

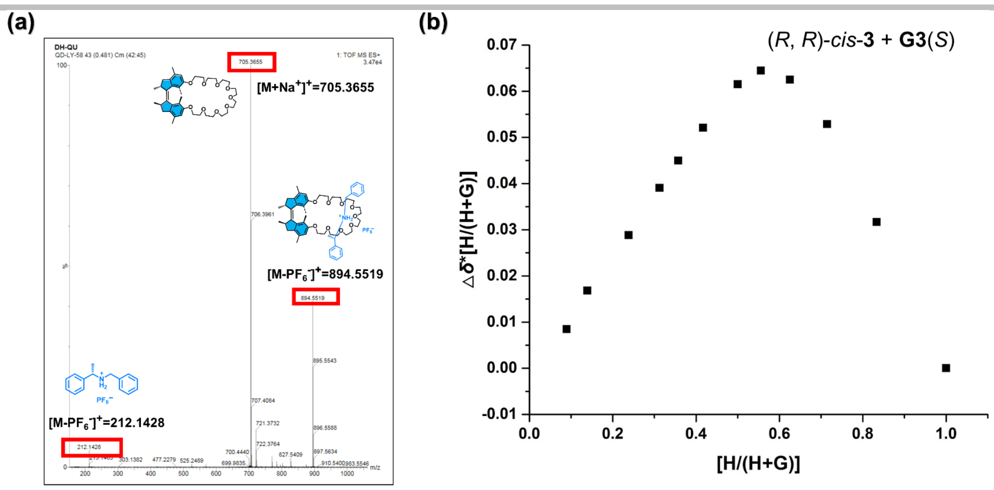

Figure S38. (a) ESI-mass spectrum of (*P*, *P*)-(*R*, *R*)-*cis*-**3** ⊃ **G3**(*S*) (894.5519). (b) Job's plot based on the proton shift of H_f in CD_2Cl_2.

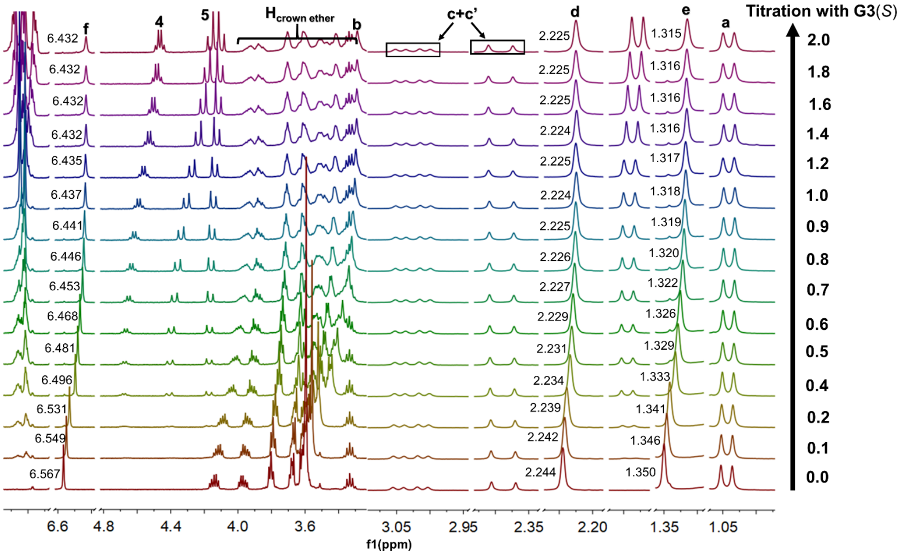

Figure S39. Partial ^{1}H NMR spectral of (*P*, *P*)-(*R*, *R*)-*cis*-**3** (5 mM, 400 MHz, 293 K, CD_2Cl_2) upon the stepwise addition of **G3**(*S*).

Table S9. Boltzmann averaged energies, dihedral angles (θ) and distances between the methyl rings of the fjord region.

	(*P*, *P*)-(*R*, *R*)-stable-*cis*-**3** ⊃ **G3**(*R*)	(*P*, *P*)-(*R*, *R*)-stable-*cis*-**3** ⊃ **G3**(*S*)	**G3**(*R*)
G (Ha)	-2873.6292	-2873.6317	-637.1560
θ (°)	7.07	6.53	
Me-Me (Å)	3.43	3.49	

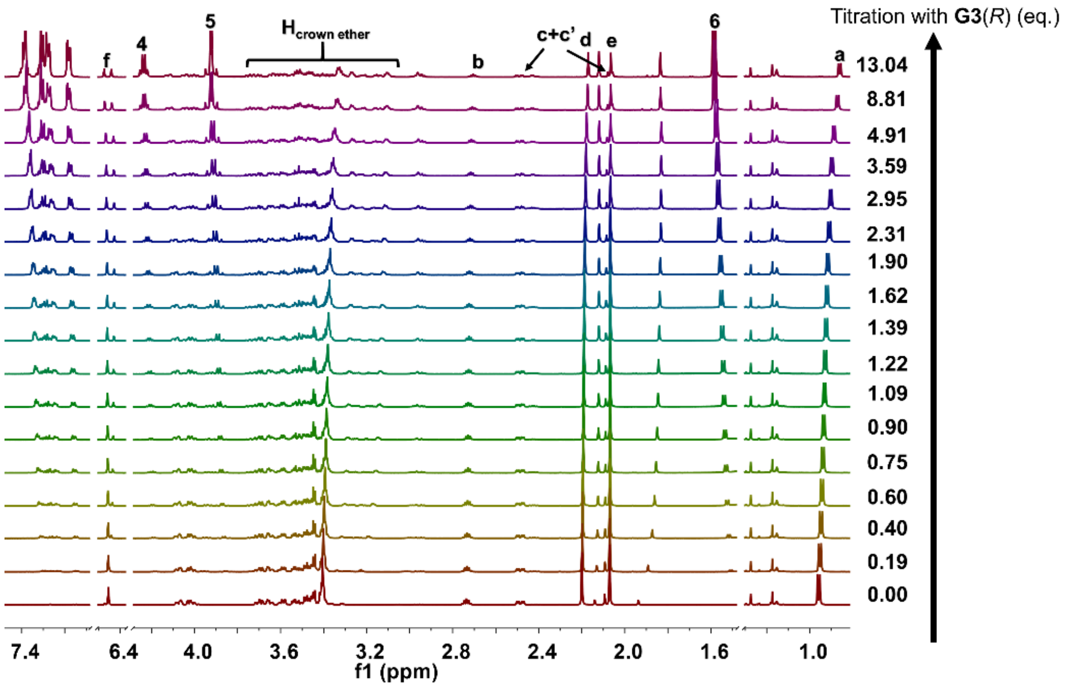

Figure S40. Partial ^{1}H NMR spectral of (*P*, *P*)-(*R*, *R*)-*trans*-**3** (3.65 mM, 400 MHz, 293 K, CD_2Cl_2) upon the stepwise addition of **G3**(*R*).

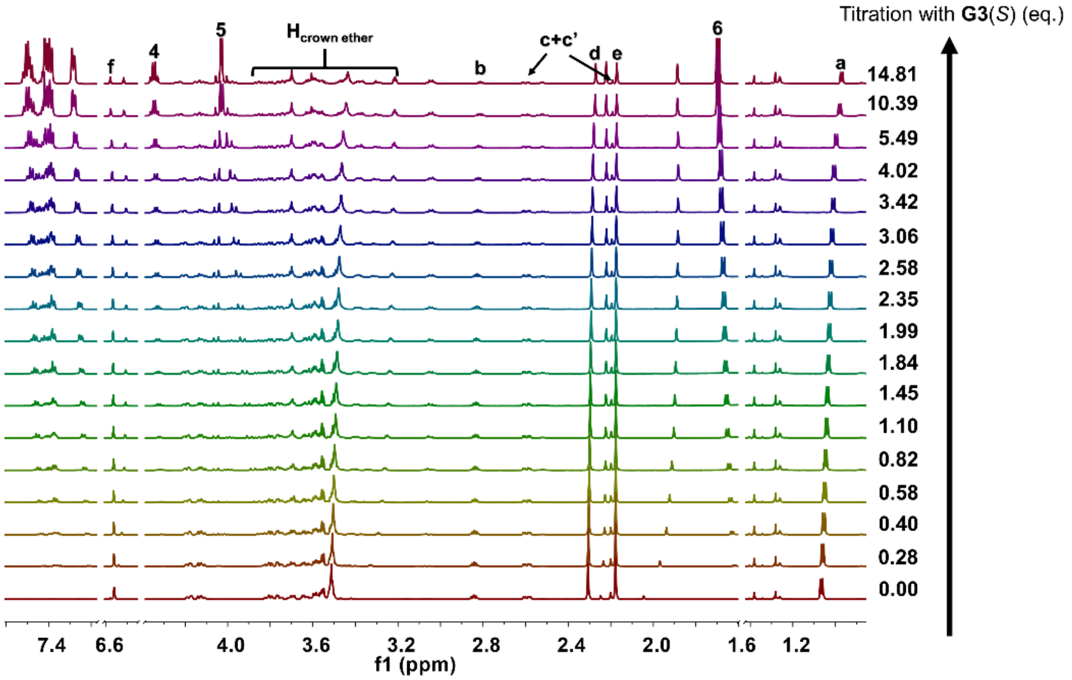

Figure S41. Partial ^{1}H NMR spectral of (*P*, *P*)-(*R*, *R*)-*trans*-**3** (3.65 mM, 400 MHz, 293 K, CD_2Cl_2) upon the stepwise addition of **G3**(*S*).

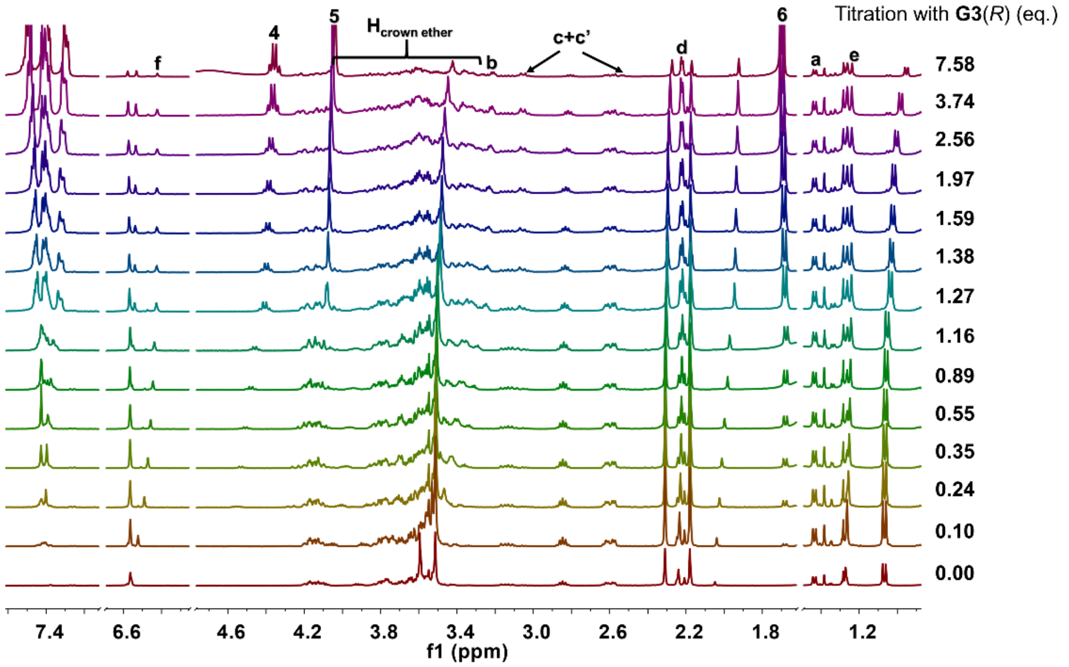

Figure S42. Partial ^{1}H NMR spectral of (*P*, *P*)-(*R*, *R*)-*trans*-**3**/(*M*, *M*)-(*S*, *S*)-*trans*-**3** (PSS_{310} mixture) (3.56 mM, 400 MHz, 293 K, CD_2Cl_2) upon the stepwise addition of **G3**(*R*).

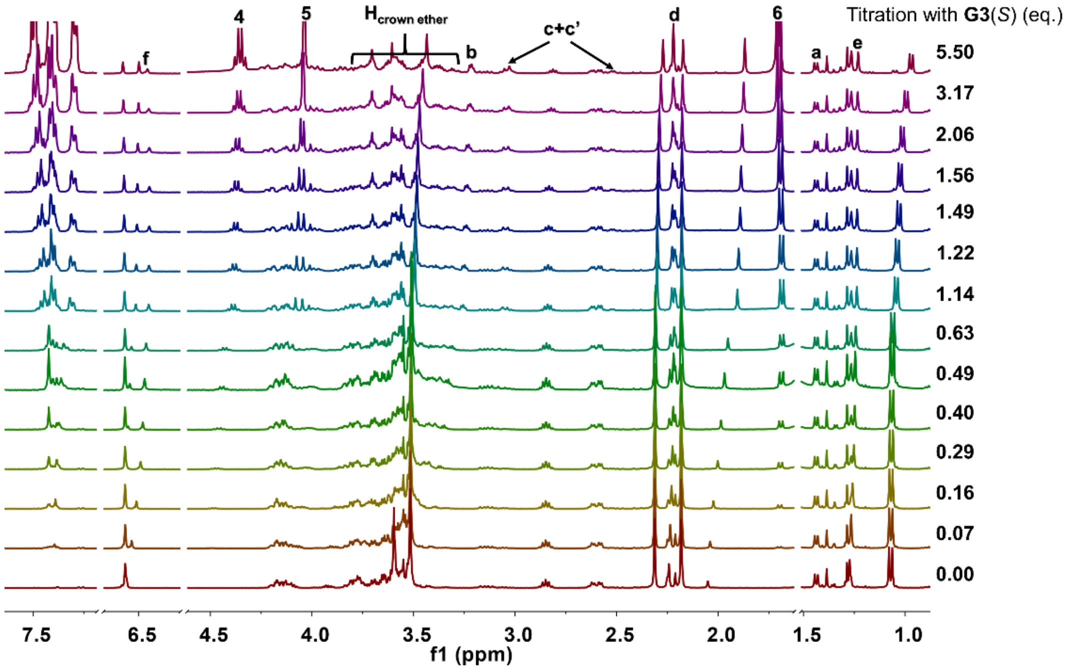

Figure S43. Partial ^{1}H NMR spectral of (*P*, *P*)-(*R*, *R*)-*trans*-**3**/(*M*, *M*)-(*S*, *S*)-*trans*-**3** (PSS_{310} mixture) (3.56 mM, 400 MHz, 293 K, CD_2Cl_2) upon the stepwise addition of **G3**(*S*).

9. Characterization

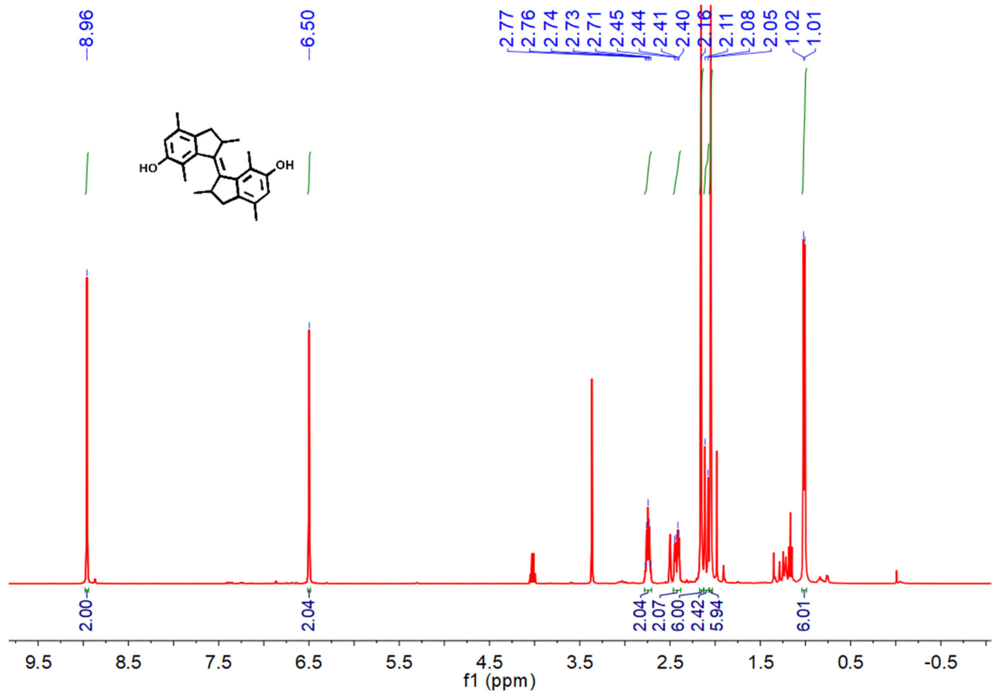

Figure S44. ^{1}H NMR spectrum of compound stable-*trans*-**2** (400 MHz, 298 K, $(CD_3)_2SO$).

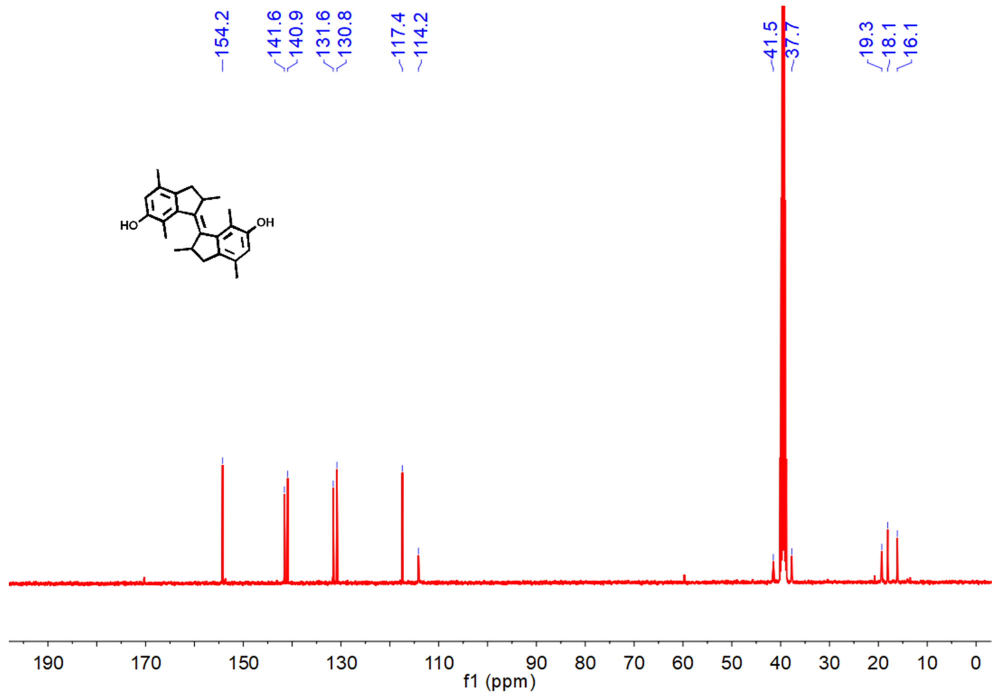

Figure S45. ^{13}C NMR spectrum of compound stable-*trans*-**2** (100 MHz, 298 K, $(CD_3)_2SO$))

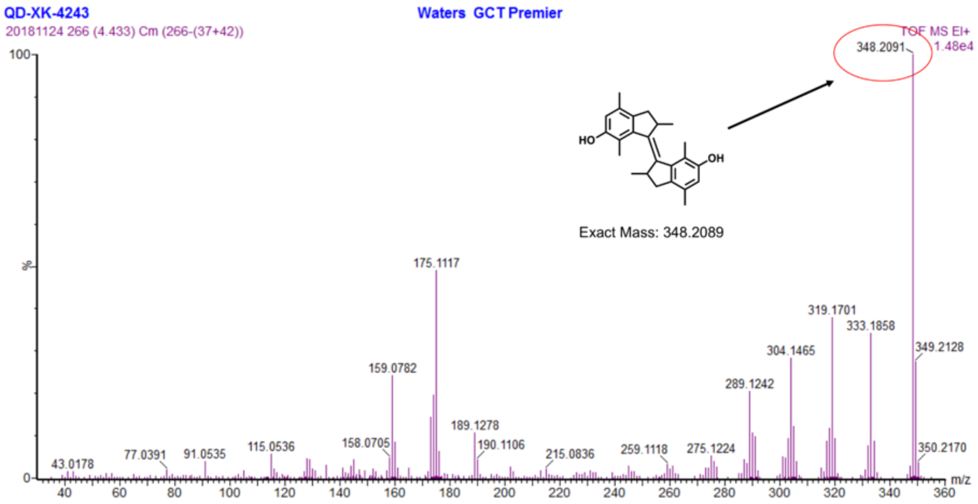

Figure S46. EI-mass spectrum of compound stable-*trans*-**2** ([M]$^{+\bullet}$: 348.2091).

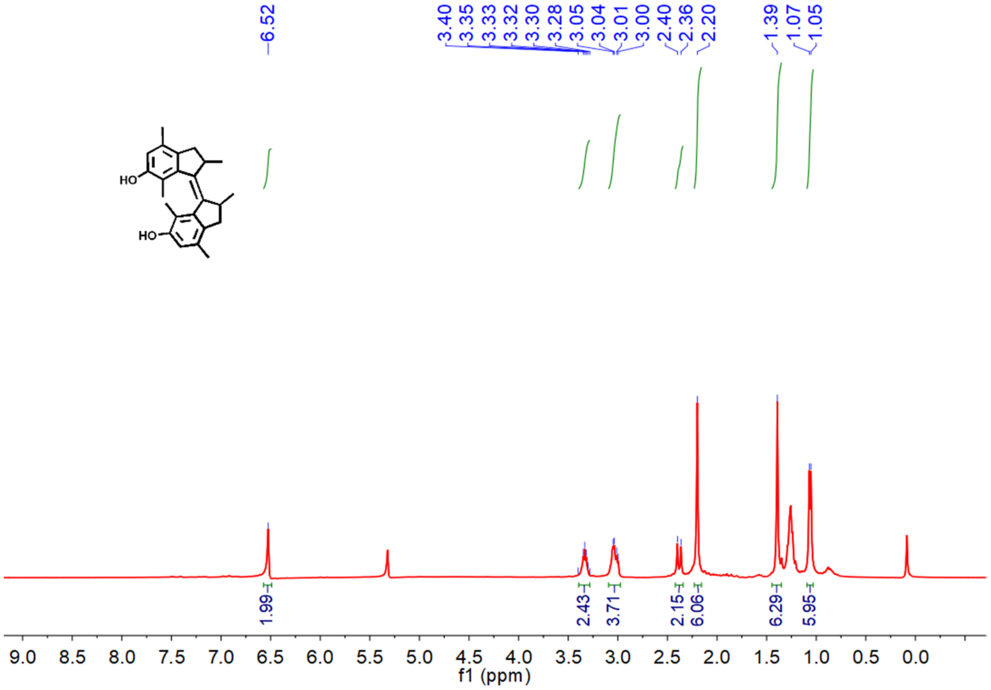

Figure S47. ^{1}H NMR spectrum of compound stable-*cis*-**2** (400 MHz, 298 K, CD_2Cl_2).

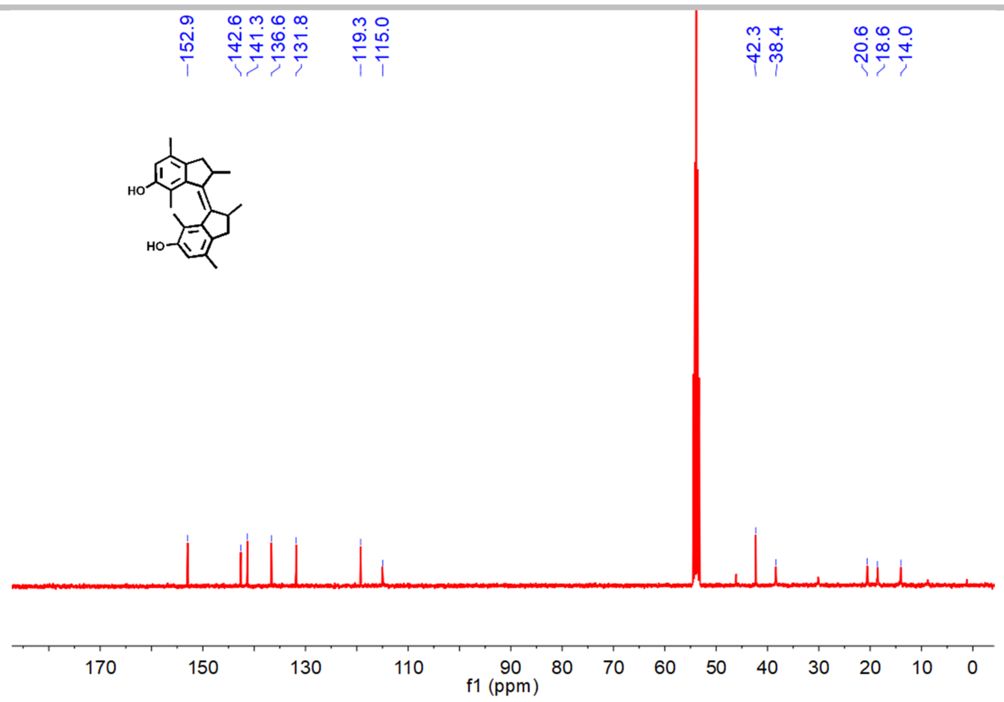

Figure S48. ^{13}C NMR spectrum of compound stable-*cis*-**2** (100 MHz, 298 K, CD_2Cl_2).

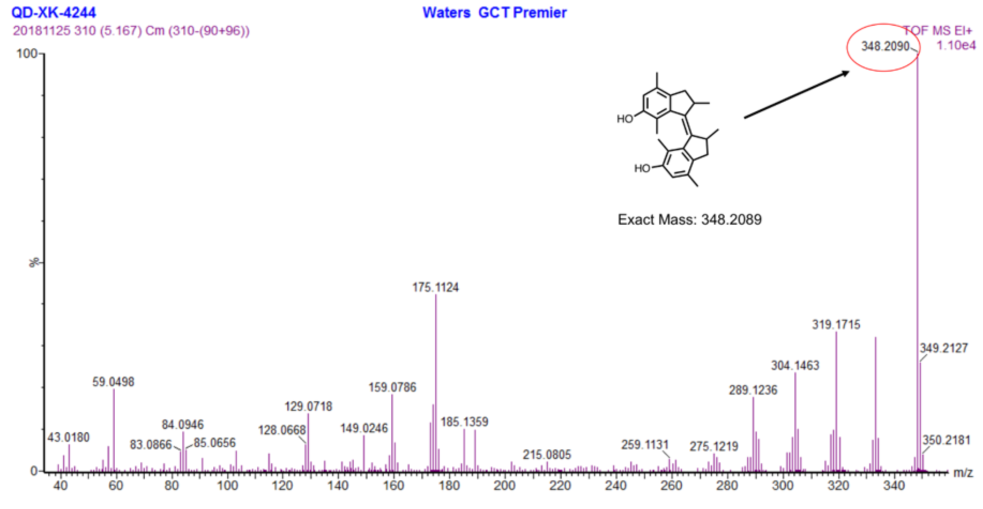

Figure S49. ESI-mass spectrum of compound stable-*cis*-**2** ($[M]^{+\bullet}$: 348.2090).

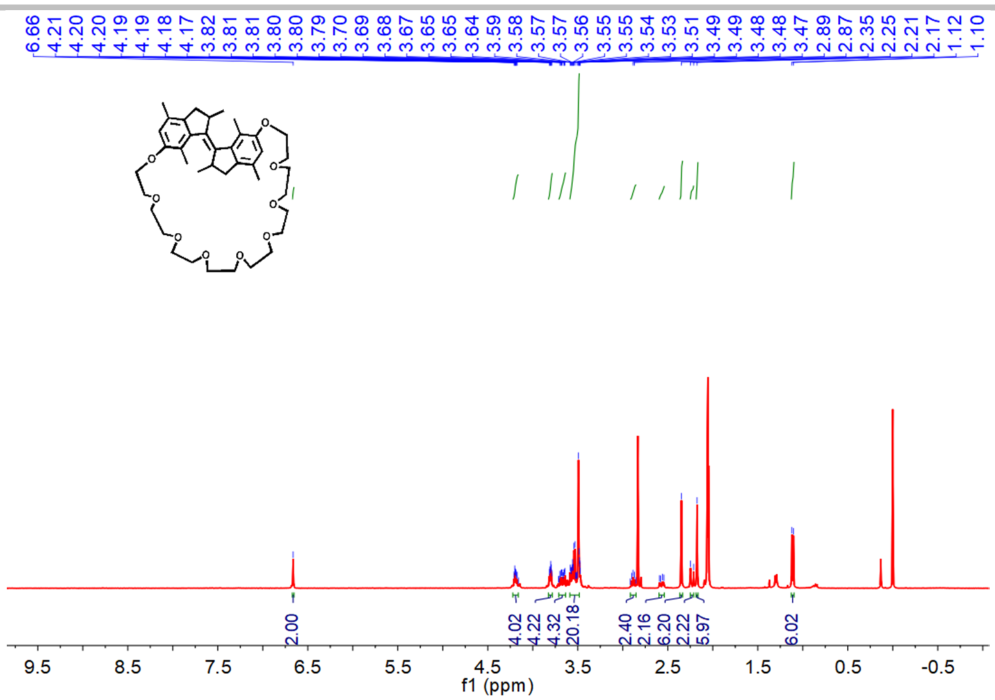

Figure S50. ^{1}H NMR spectrum of compound stable-*trans*-**3** (400 MHz, 298 K, $(CD_3)_2CO$).

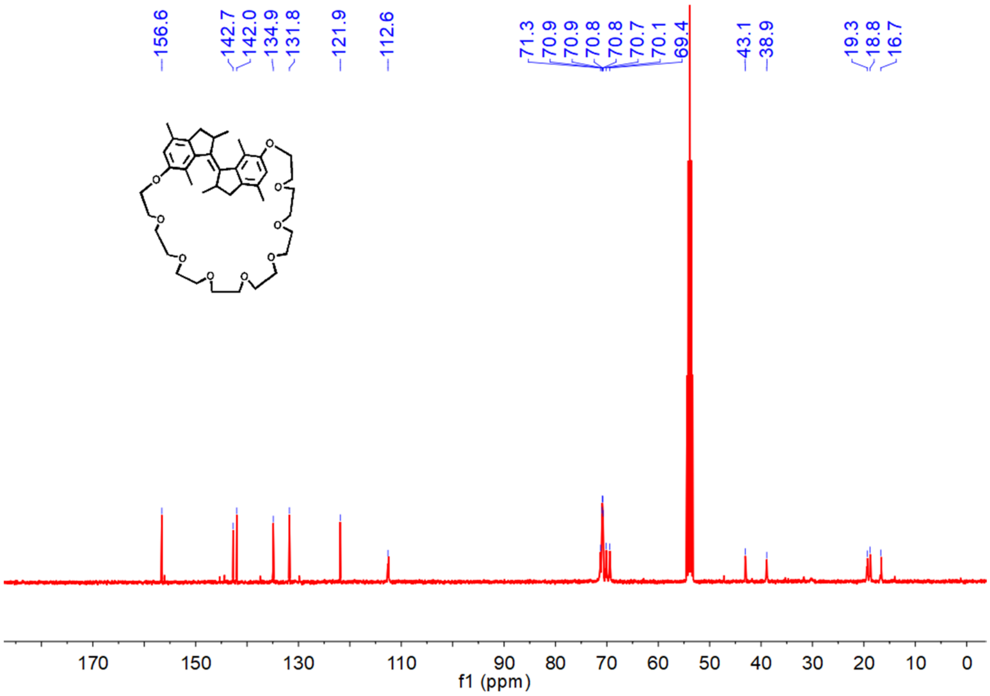

Figure S51. ^{13}C NMR spectrum of compound stable-*cis*-**3** (100 MHz, 298 K, CD_2Cl_2).

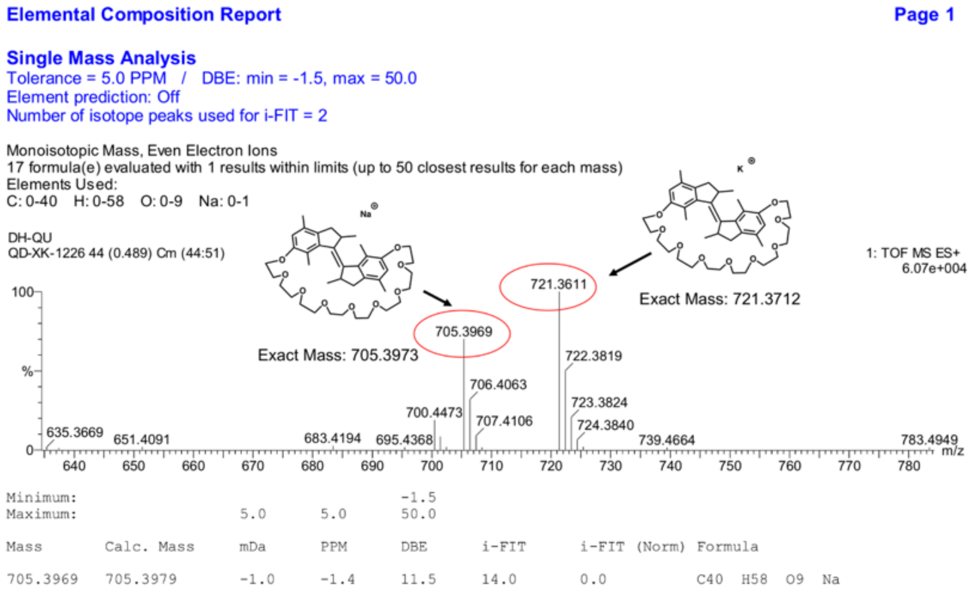

Figure S52. ESI-mass spectrum of compound stable-*trans*-**3** ([M+Na]$^{+}$: 705.3969).

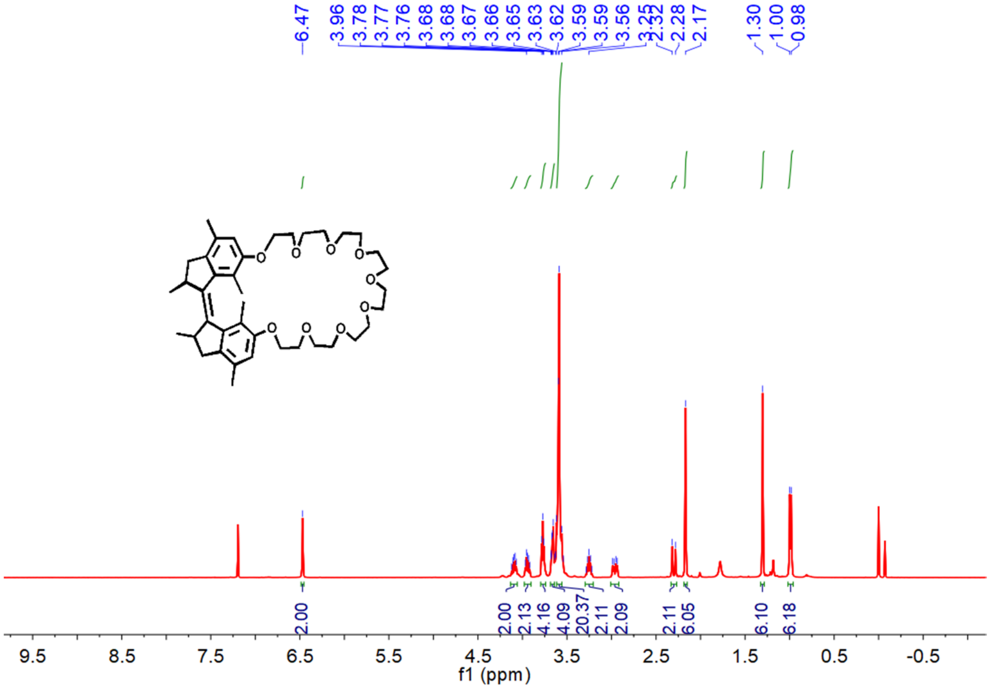

Figure S53. ^{1}H NMR spectrum of compound stable-*cis*-**3** (400 MHz, 298 K, $CDCl_3$).

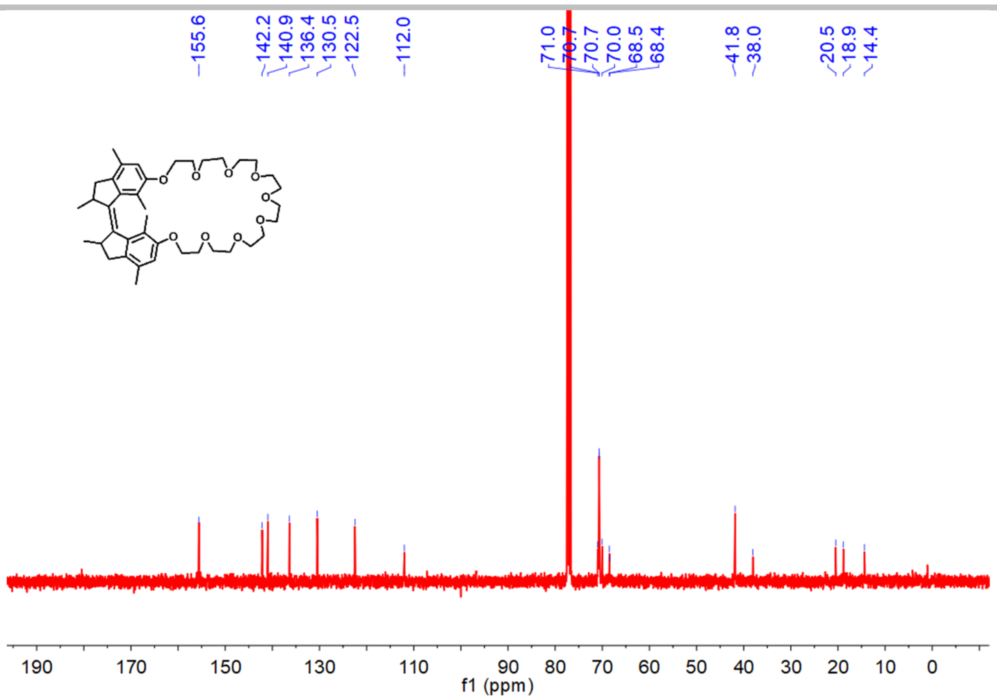

Figure S54. ^{13}C NMR spectrum of compound stable-*cis*-**3** (100 MHz, 298 K, $CDCl_3$).

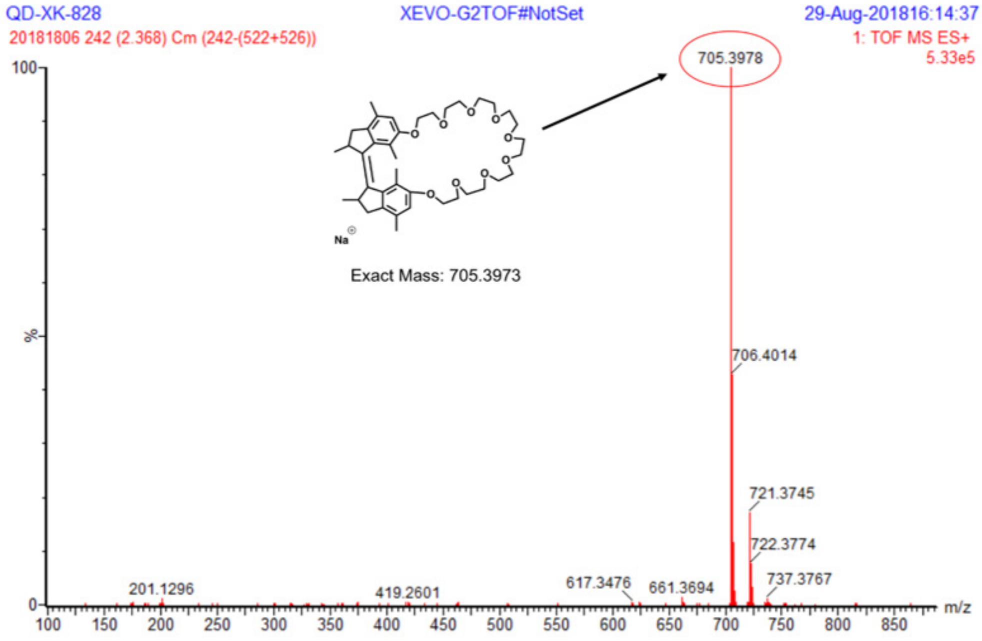

Figure S55. ESI-mass spectrum of compound stable-*cis*-**3** ([M+Na]$^+$: 705.3978).

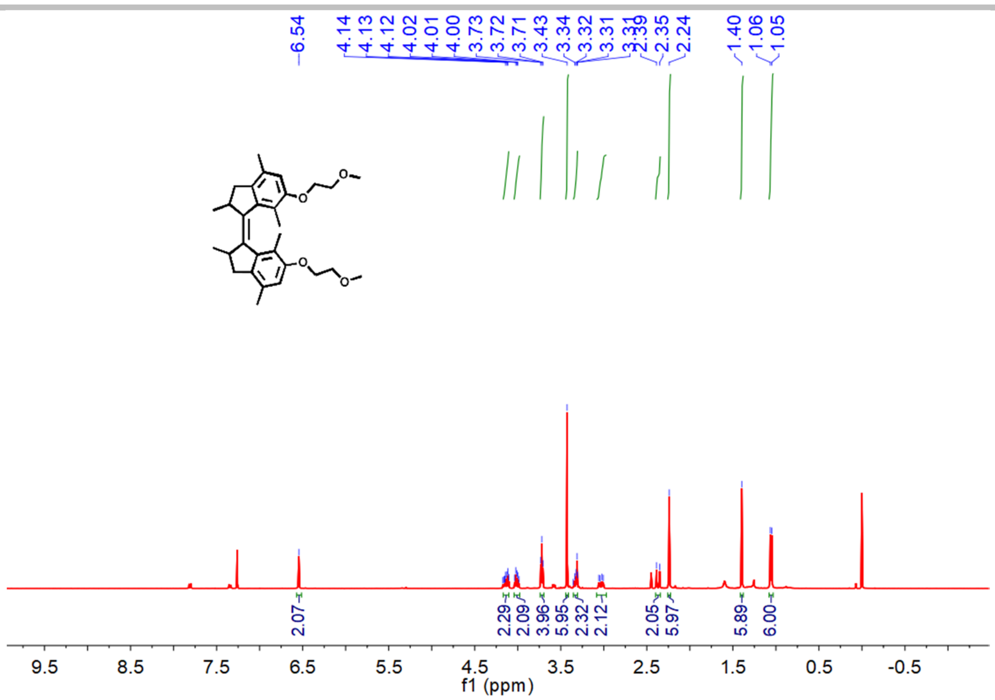

Figure S56. ^{1}H NMR spectrum of the reference compounds stable-*cis*-**R_1** (400 MHz, 298 K, $CDCl_3$).

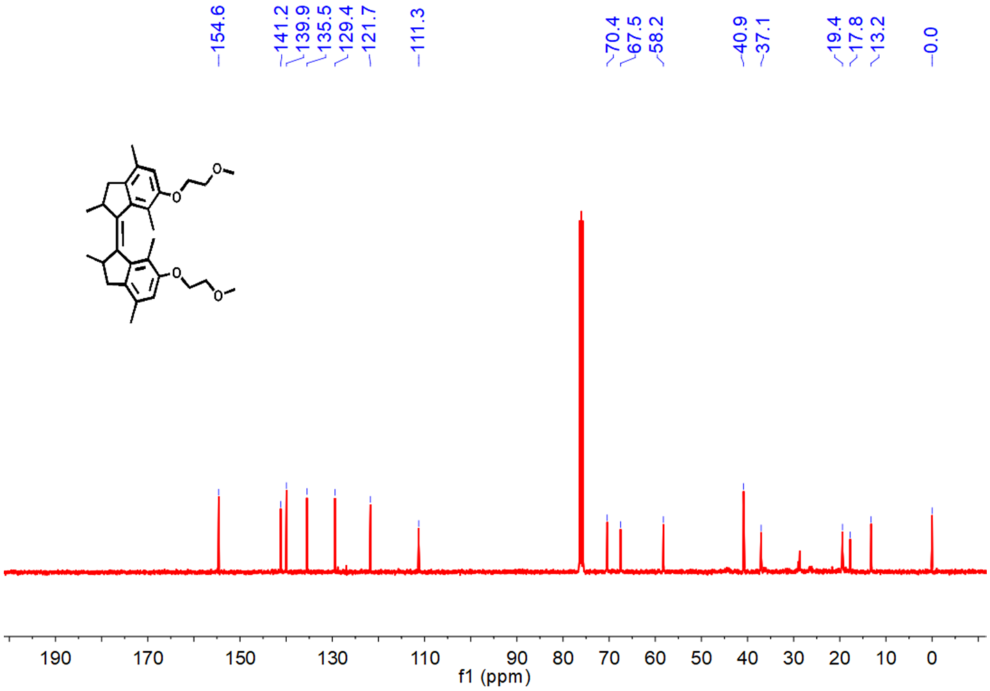

Figure S57. ^{13}C NMR spectrum of compound stable-*cis*-**R_1** (100 MHz, 298 K, $CDCl_3$).

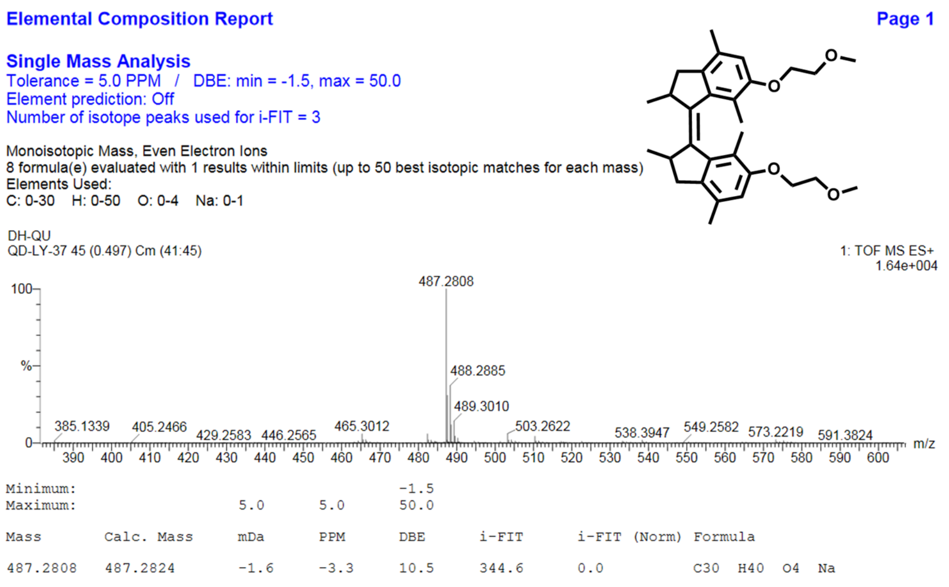

Figure S58. ESI-mass spectrum of the reference compounds stable-*cis*-$\mathbf{R_1}$ ($[M+Na]^+$: 487.2808).

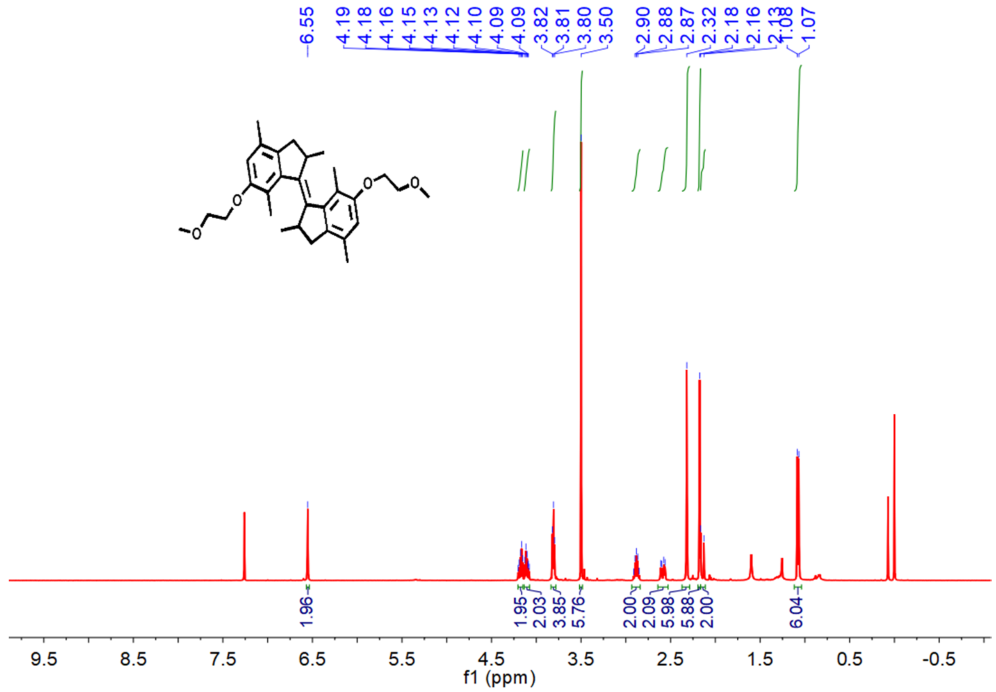

Figure S59. ^{1}H NMR spectrum of the reference compounds stable-*trans*-$\mathbf{R_1}$ (400 MHz, 298 K, $CDCl_3$).

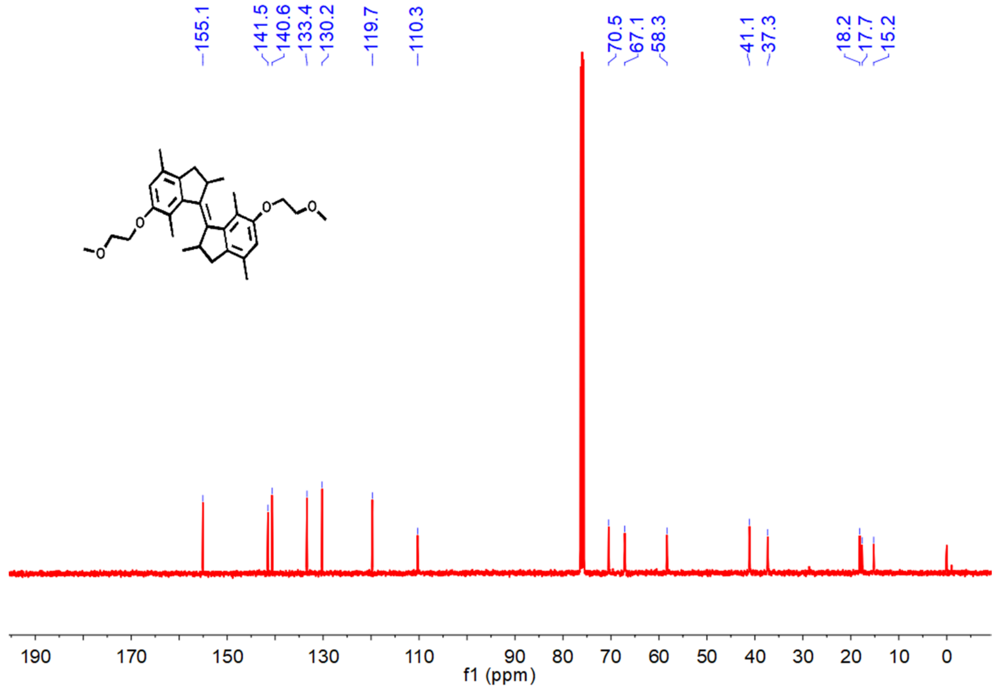

Figure S60. ^{13}C NMR spectrum of compound stable-*trans*-$\mathbf{R_1}$ (100 MHz, 298 K, $CDCl_3$).

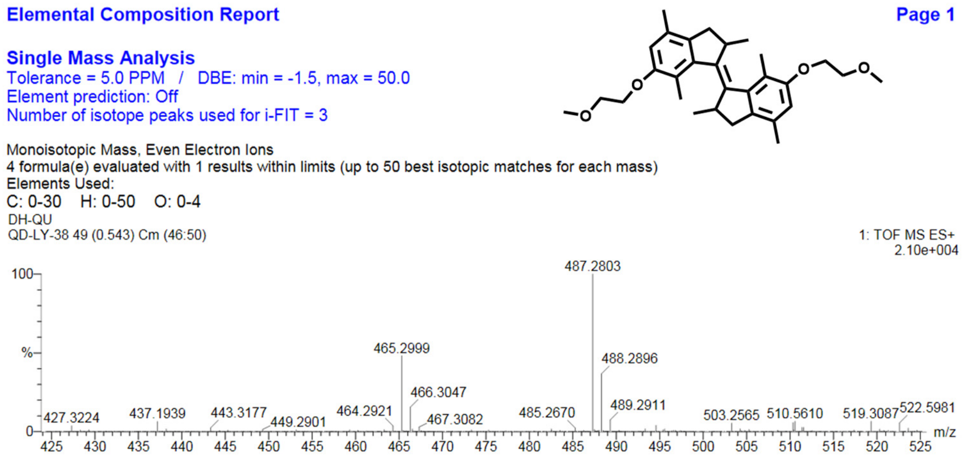
Elemental Composition Report

Page 1

Single Mass Analysis
Tolerance = 5.0 PPM / DBE: min = -1.5, max = 50.0
Element prediction: Off
Number of isotope peaks used for i-FIT = 3

Monoisotopic Mass, Even Electron Ions
4 formula(e) evaluated with 1 results within limits (up to 50 best isotopic matches for each mass)
Elements Used:
C: 0-30 H: 0-50 O: 0-4
DH-QU
QD-LY-38 49 (0.543) Cm (46:50)

1: TOF MS ES+
2.10e+004

Minimum:				-1.5			
Maximum:		5.0	5.0	50.0			
Mass	Calc. Mass	mDa	PPM	DBE	i-FIT	i-FIT (Norm)	Formula
465.2999	465.3005	-0.6	-1.3	10.5	281.5	0.0	C30 H41 O4

Figure S61. ESI-mass spectrum of the reference compounds stable-*trans*-$\mathbf{R_1}$ ($[M+H]^+$: 465.2999, $[M+Na]^+$: 487.2803).

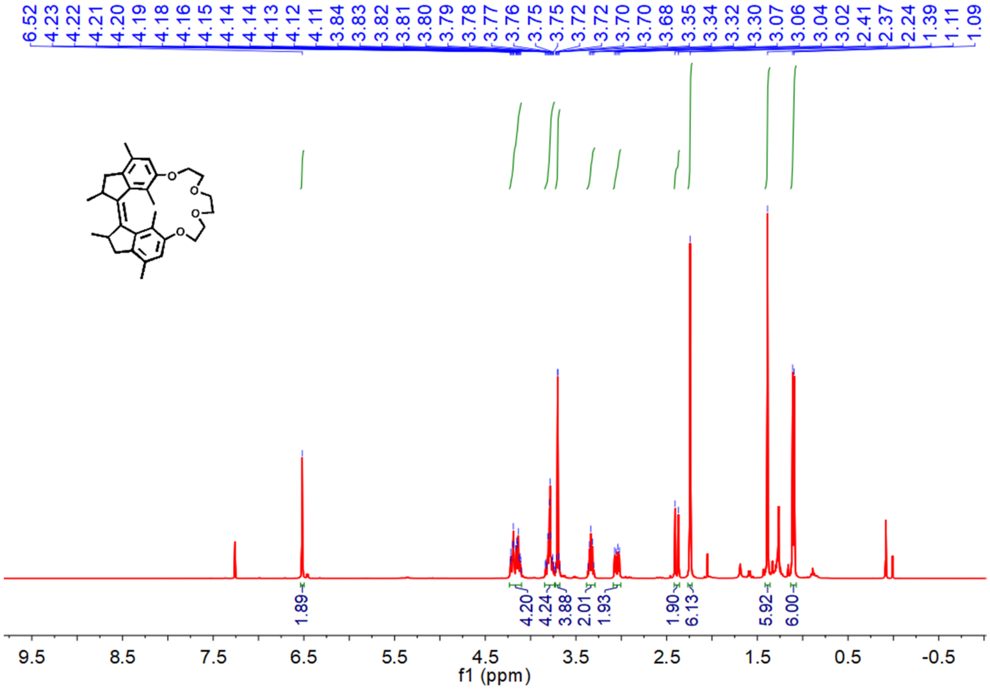

Figure S62. ^{1}H NMR spectrum of the reference compounds stable-*cis*-**R2** (400 MHz, 298 K, $CDCl_3$).

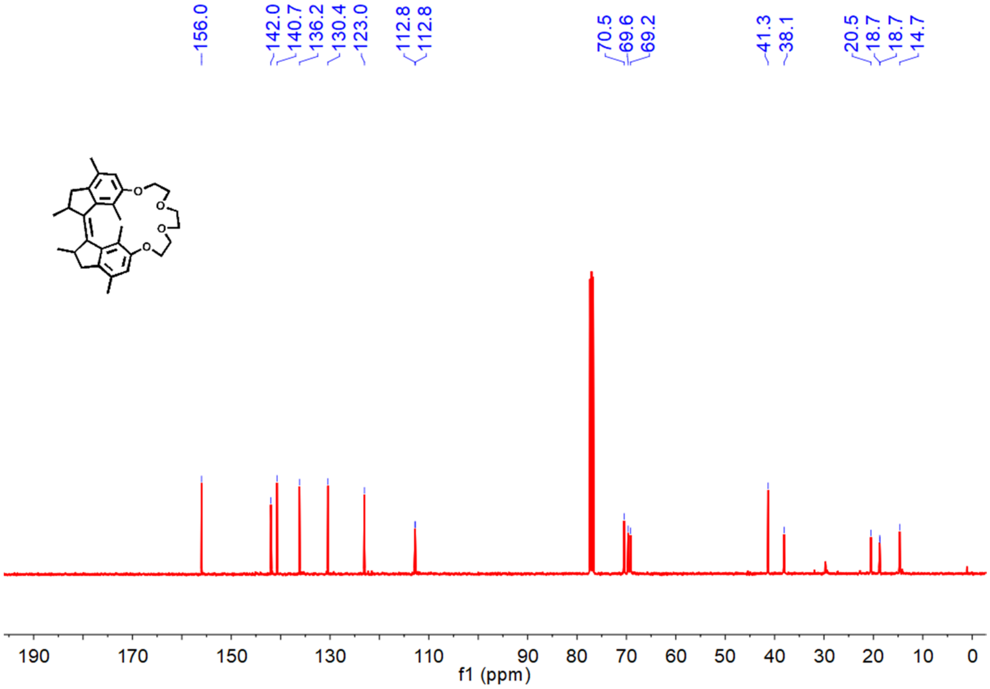

Figure S63. ^{13}C NMR spectrum of compound stable-*cis*-**R2** (100 MHz, 298 K, $CDCl_3$).

Elemental Composition Report **Page 1**

Single Mass Analysis
Tolerance = 5.0 mDa / DBE: min = -1.5, max = 50.0
Element prediction: Off
Number of isotope peaks used for i-FIT = 3

Monoisotopic Mass, Even Electron Ions
7 formula(e) evaluated with 1 results within limits (up to 50 best isotopic matches for each mass)
Elements Used:
C: 0-30 H: 0-38 O: 0-4 Na: 0-1

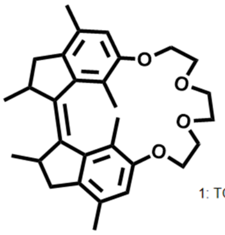

DH-QU
QD-LY-32 56 (0.628) Cm (56:64)

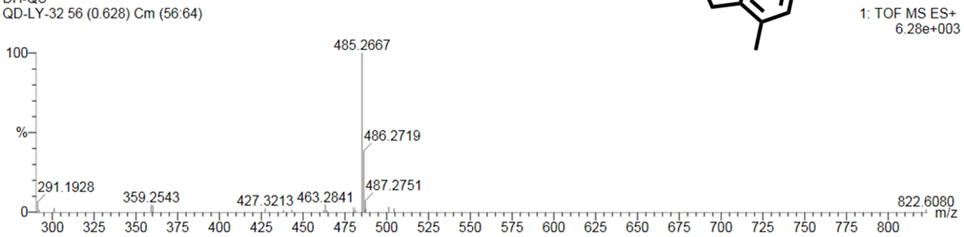

Minimum:				-1.5			
Maximum:		5.0	10.0	50.0			
Mass	Calc. Mass	mDa	PPM	DBE	i-FIT	i-FIT (Norm)	Formula
485.2667	485.2668	-0.1	-0.2	11.5	21.7	0.0	C30 H38 O4 Na

Figure S64. ESI-mass spectrum of the reference compounds stable-*cis*-**R**$_2$ ([M+Na]$^+$: 485.2667).

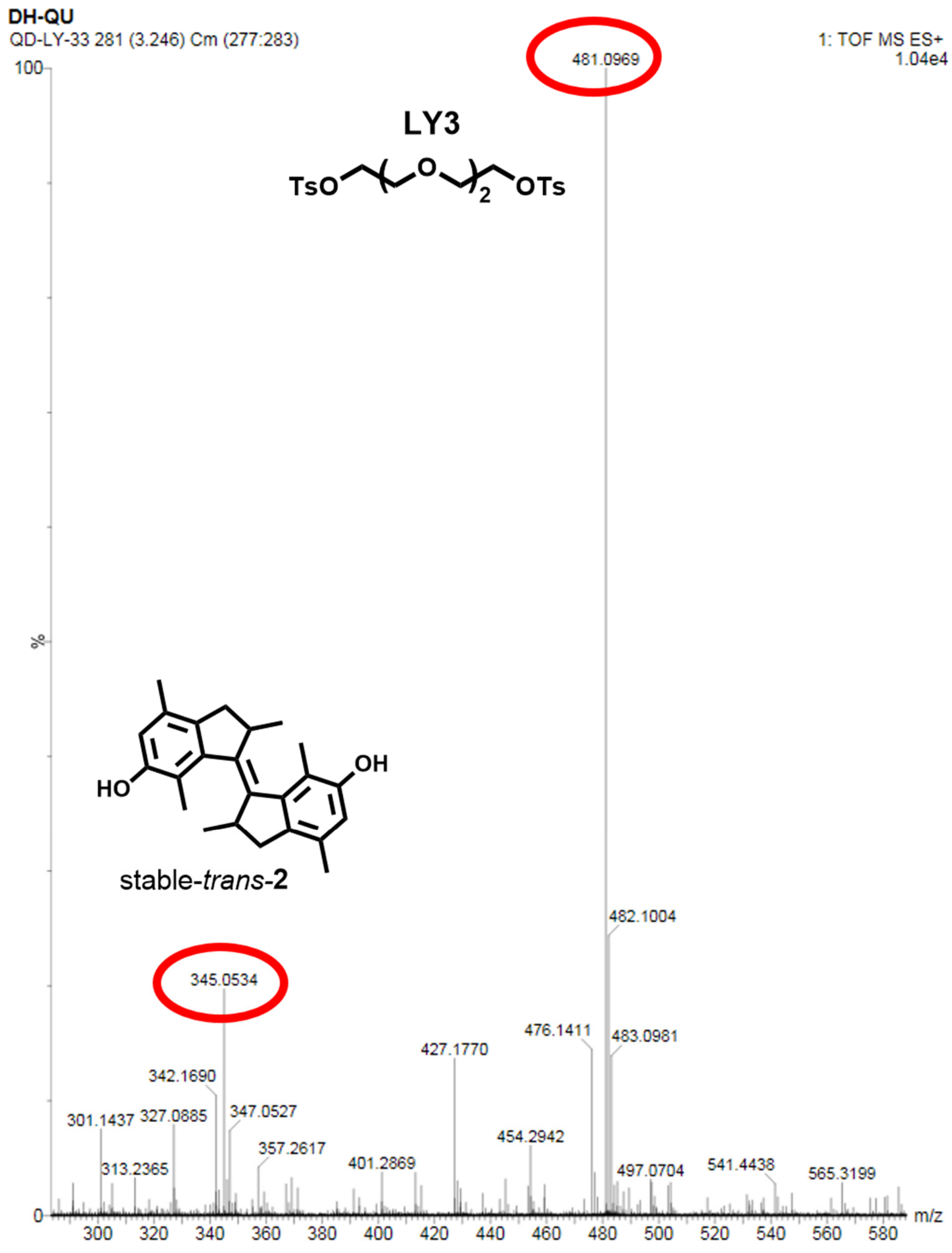

Figure S65. ESI-mass spectrum of the reaction solution between stable-*trans*-**2** and **LY3**.

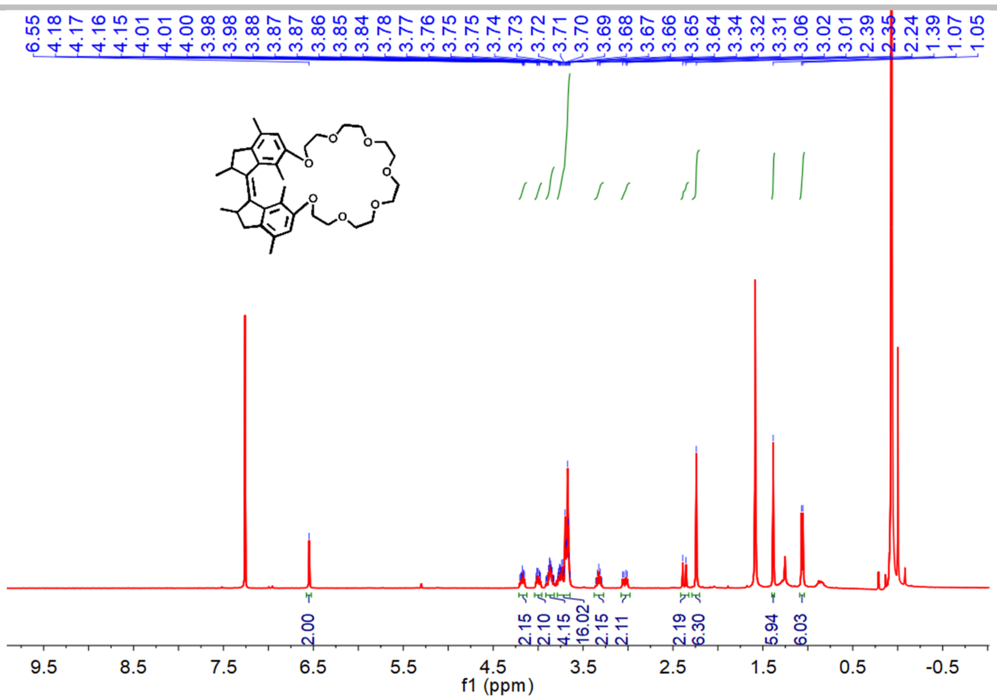

Figure S66. ^{1}H NMR spectrum of the reference compounds stable-*cis*-**R$_3$** (400 MHz, 298 K, $CDCl_3$).

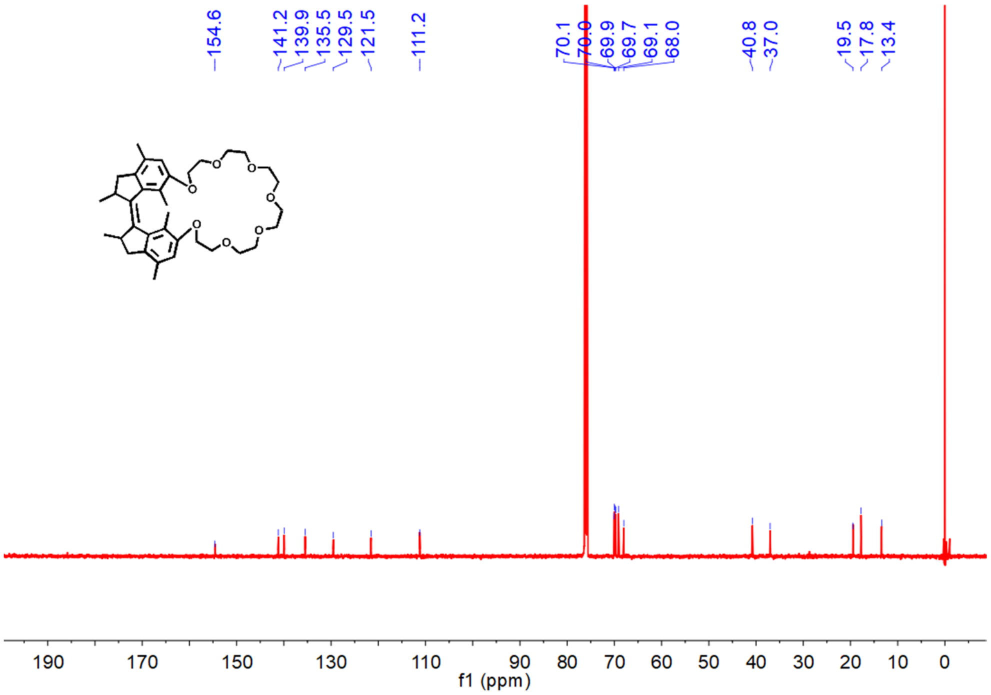

Figure S67. ^{13}C NMR spectrum of compound stable-*cis*-**R$_3$** (150 MHz, 298 K, $CDCl_3$).

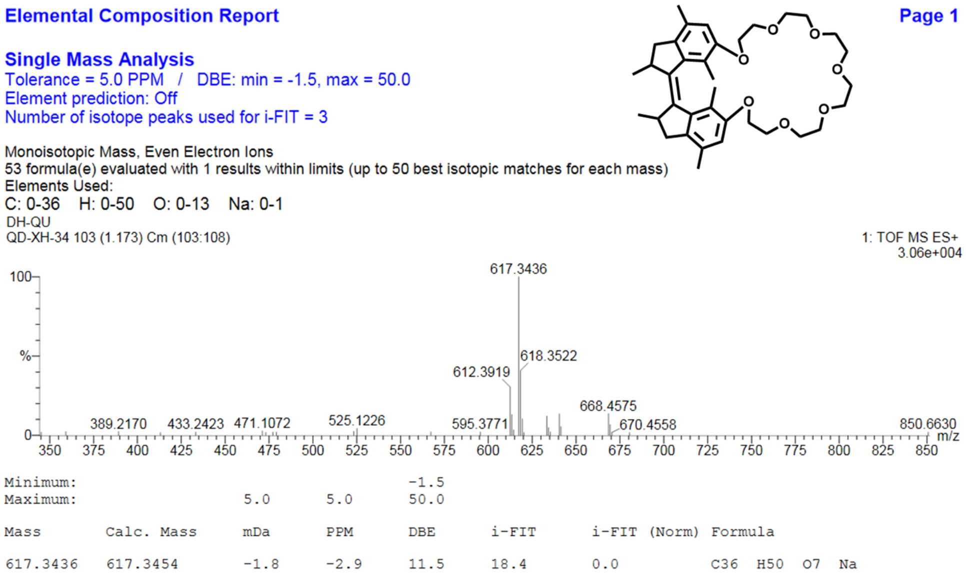

Figure S68. ESI-mass spectrum of the reference compounds stable-*cis*-$\mathbf{R_3}$ ([M+Na]$^+$: 617.3436).

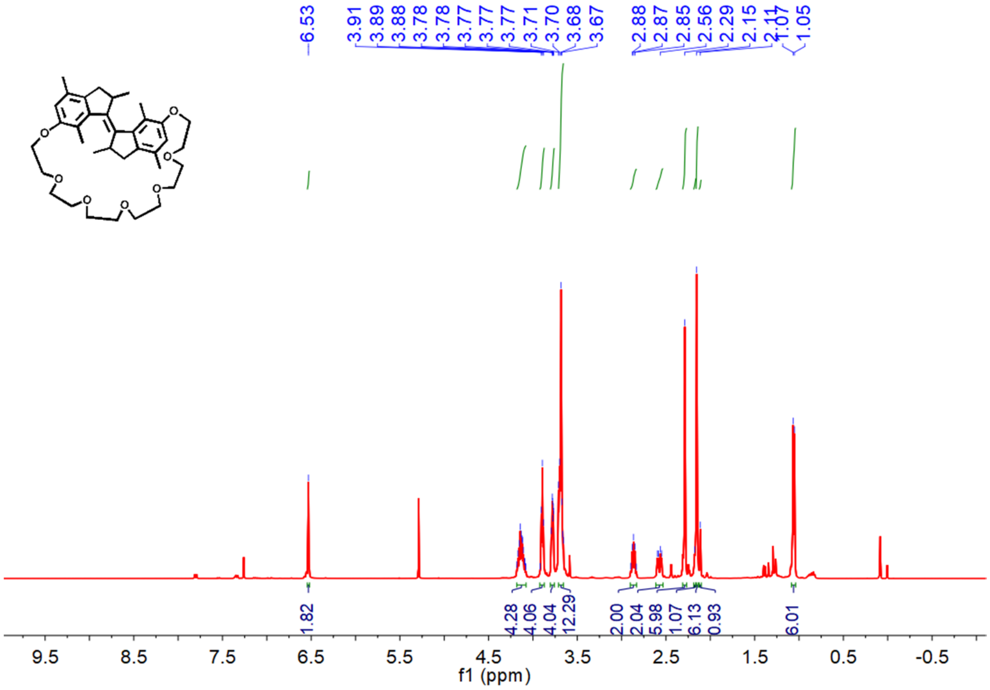

Figure S69. ^{1}H NMR spectrum of the reference compounds stable-*trans*-$\mathbf{R_3}$ (400 MHz, 298 K, $CDCl_3$).

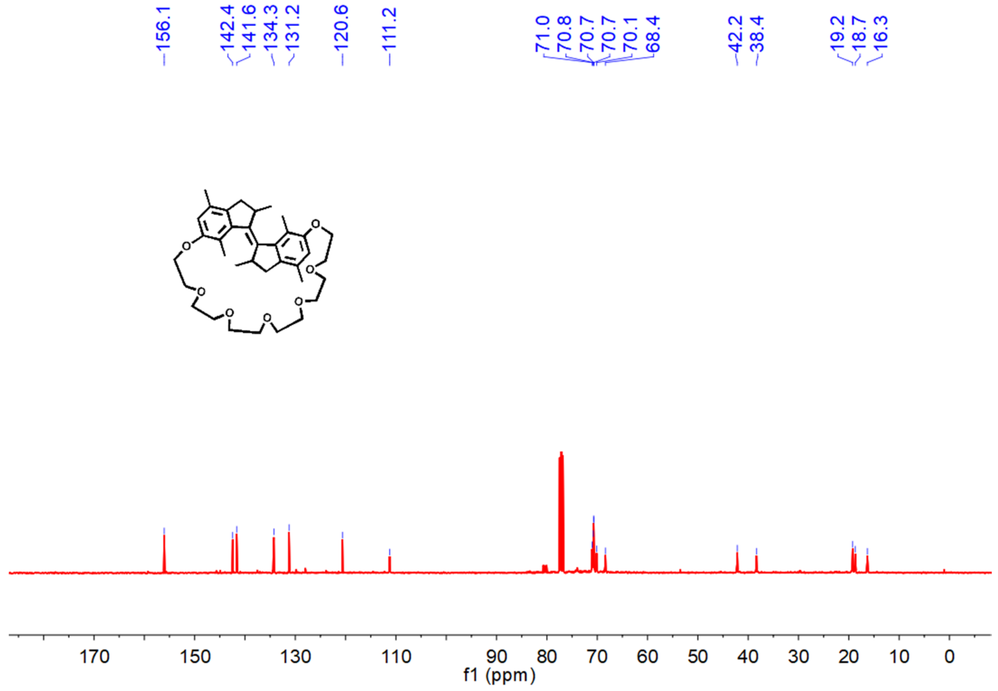

Figure S70. ^{13}C NMR spectrum of compound stable-*trans*-**R$_3$** (100 MHz, 298 K, $CDCl_3$).

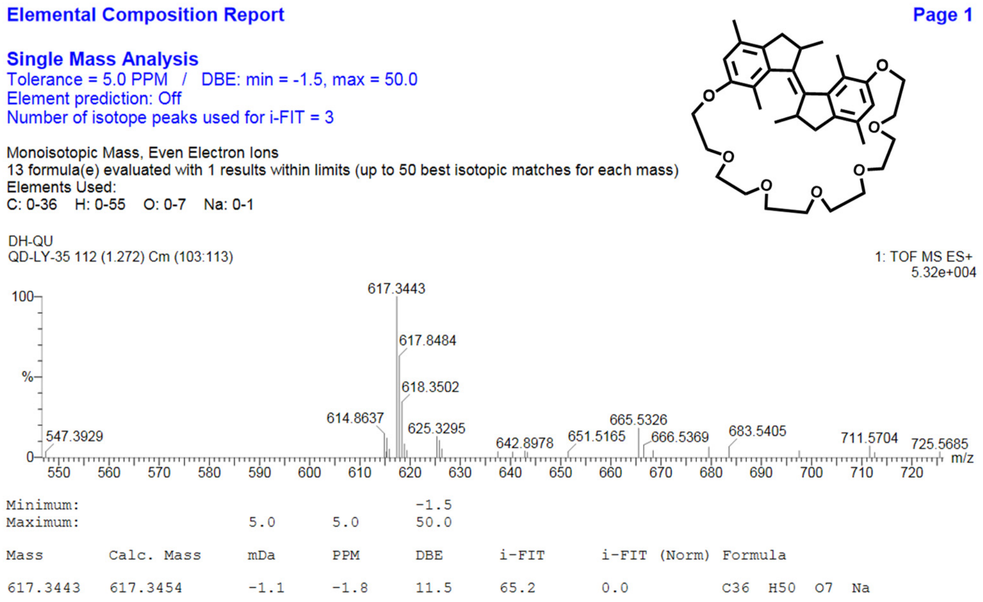

Figure S71. ESI-mass spectrum of the reference compounds stable-*trans*-**R$_3$** ($[M+Na]^+$: 617.3443).

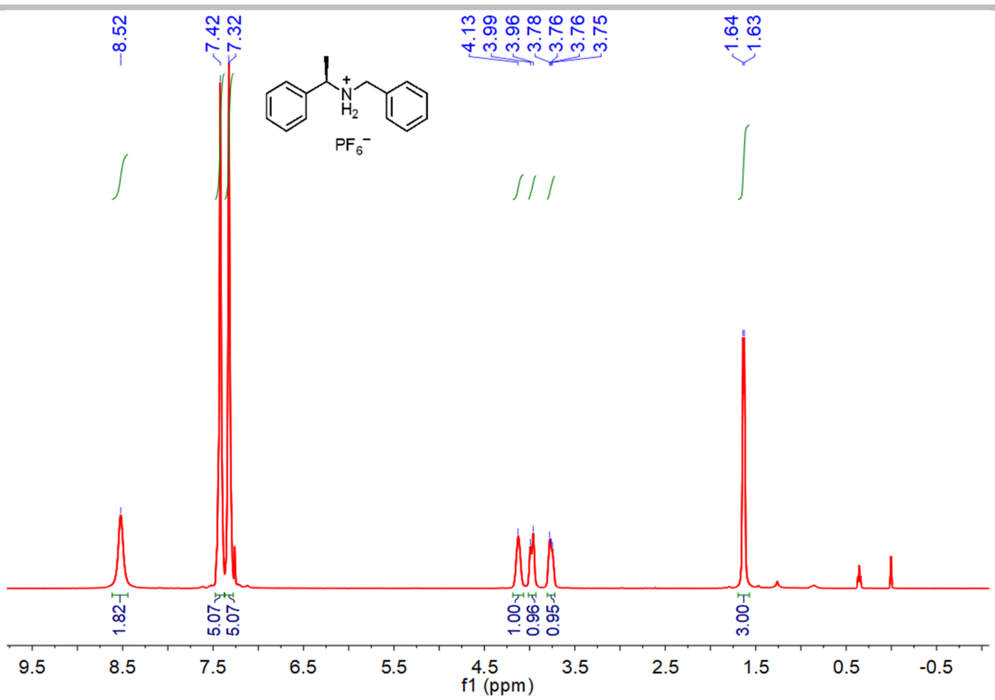

Figure S72. ^{1}H NMR spectrum of compound **G3**(*R*) (400 MHz, 298 K, $CDCl_3$).

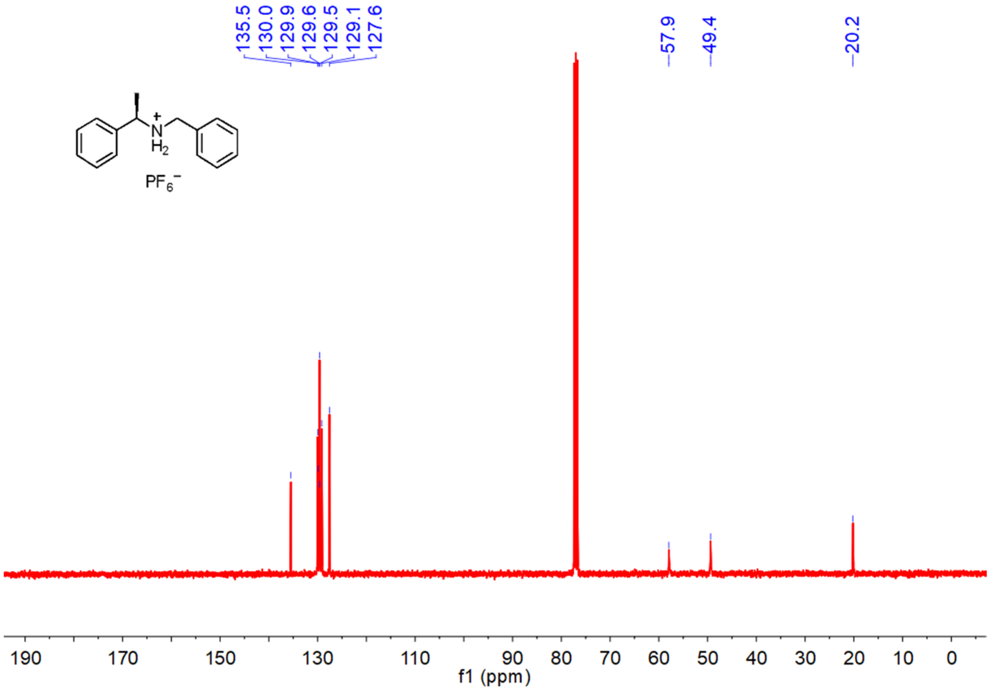

Figure S73. ^{13}C NMR spectrum of compound **G3**(*R*) (100 MHz, 298 K, $CDCl_3$).

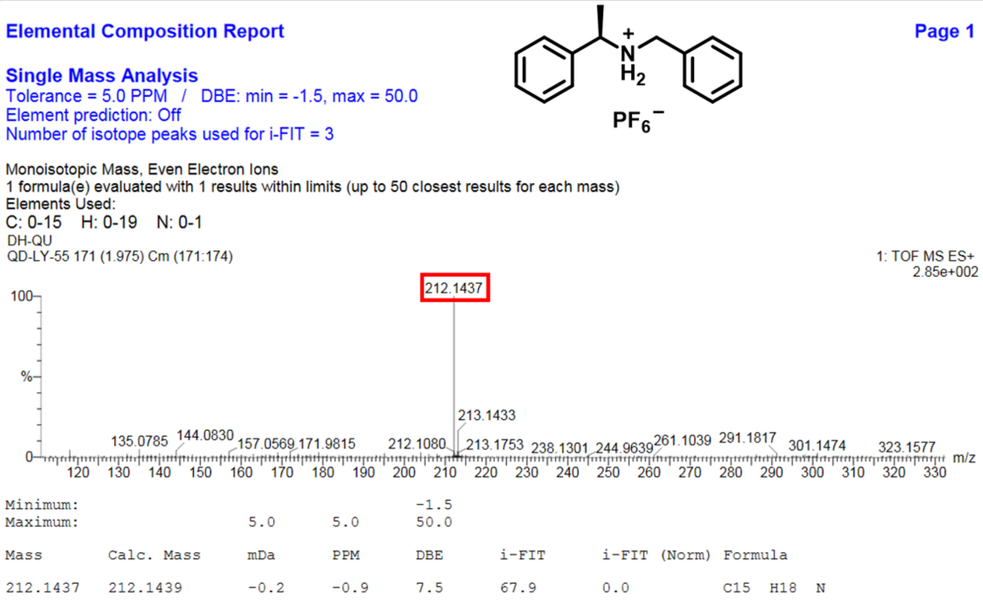

Figure S74. ESI-mass spectrum of compounds **G3**(*R*) ([M-PF_6^-]$^+$: 212.1437).

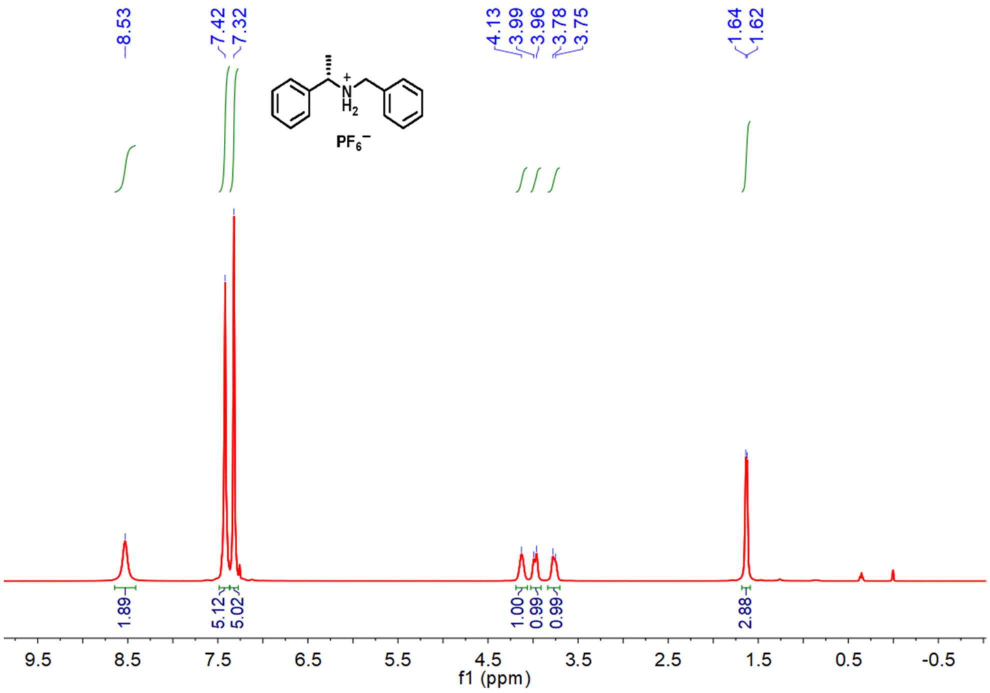

Figure S75. ^{1}H NMR spectrum of compound **G3**(*S*) (400 MHz, 298 K, $CDCl_3$).

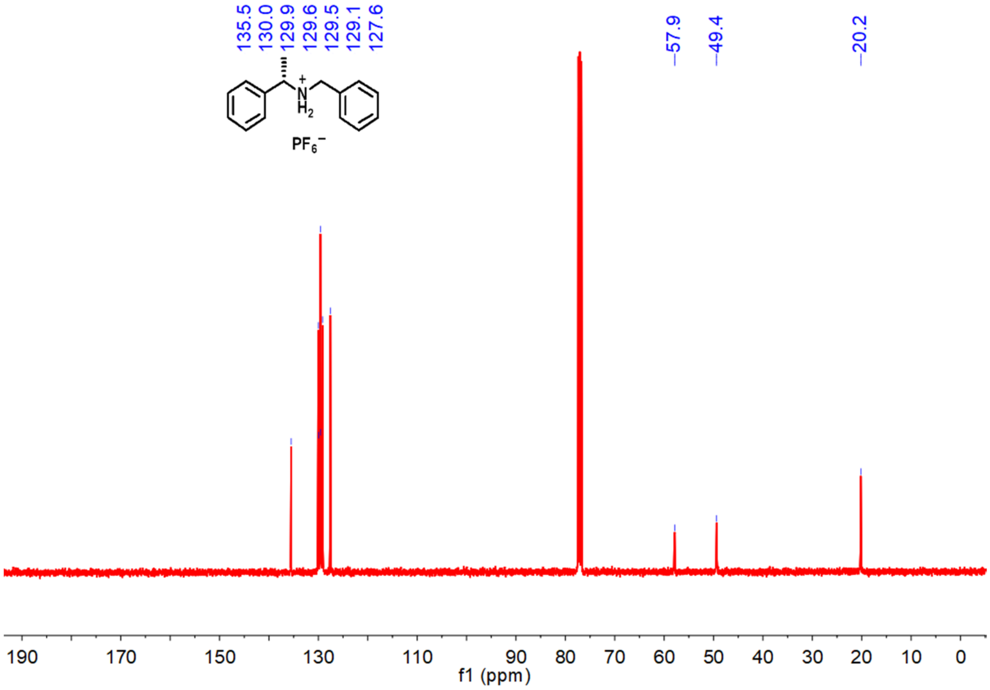

Figure S76. ^{13}C NMR spectrum of compound **G3**(*S*) (100 MHz, 298 K, $CDCl_3$).

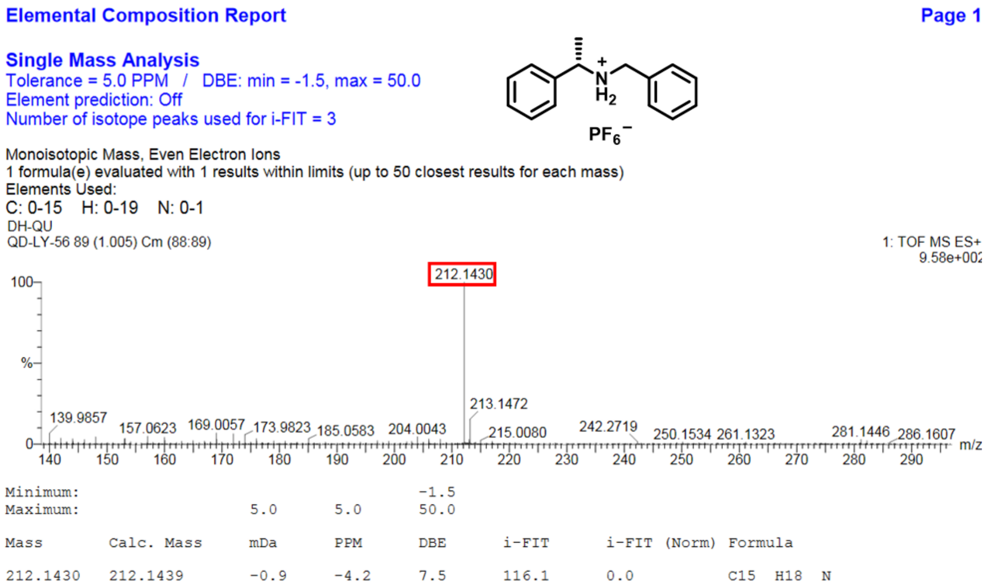

Figure S77. ESI-mass spectrum of compounds **G3**(*S*) ($[M-PF_6^-]^+$: 212.1430).

10. References

[1] T. M. Neubauer, T. van Leeuwen, D. Zhao, A. S. Lubbe, J. C. M. Kistemaker, B. L. Feringa, *Org. Lett.* **2014**, *16*, 4220-4223.

[2] L. Yu, Q. Yang, J. Xiang, H. Sun, L. Wang, Q. Li, A. Guan, Y. Tang, *Analyst* **2015**, *140*, 1637-1646.

[3] L. Ji, Z. Yang, Y. Zhao, M. Sun, L. Cao, X.-J. Yang, Y.-Y. Wang, B. Wu, *Chem. Commun.* **2016**, *52*, 7310-7313.

[4] Y. Liu, Q. Zhang, W.-H. Jin, T.-Y. Xu, D.-H. Qu, H. Tian, *Chem. Commun.* **2018**, *54*, 10642-10645.

[5] P. M. Reichstein, S. Gödrich, G. Papastavrou, M. Thelakkat, *Macromolecules* **2016**, *49*, 5484-5493.

[6] C. Zhang, S. Li, J. Zhang, K. Zhu, N. Li, F. Huang, *Org. Lett.* **2007**, *9*, 5553-5556.

[7] B. Spingler, S. Schnidrig, T. Todorova, F. Wild, *CrystEngComm* **2012**, *14*, 751-757.

[8] O. V. Dolomanov, L. J. Bourhis, R. J. Gildea, J. A. K. Howard, H. Puschmann, *J. Appl. Cryst.* **2009**, *42*, 339-341.

[9] P. Pracht, F. Bohle, S. Grimme, *Phys. Chem. Chem. Phys.* **2020**, *22*, 7169-7192.

[10] Gaussian 16, Revision B.01, M. J. Frisch, G. W. Trucks, H. B. Schlegel, G. E. Scuseria, M. A. Robb, J. R. Cheeseman, G. Scalmani, V. Barone, G. A. Petersson, H. Nakatsuji, X. Li, M. Caricato, A. V. Marenich, J. Bloino, B. G. Janesko, R. Gomperts, B. Mennucci, H. P. Hratchian, J. V. Ortiz, A. F. Izmaylov, J. L. Sonnenberg, D. Williams-Young, F. Ding, F. Lipparini, F. Egidi, J. Goings, B. Peng, A. Petrone, T. Henderson, D. Ranasinghe, V. G. Zakrzewski, J. Gao, N. Rega, G. Zheng, W. Liang, M. Hada, M. Ehara, K. Toyota, R. Fukuda, J. Hasegawa, M. Ishida, T. Nakajima, Y. Honda, O. Kitao, H. Nakai, T. Vreven, K. Throssell, J. A. Montgomery, Jr., J. E. Peralta, F. Ogliaro, M. J. Bearpark, J. J. Heyd, E. N. Brothers, K. N. Kudin, V. N. Staroverov, T. A. Keith, R. Kobayashi, J. Normand, K. Raghavachari, A. P. Rendell, J. C. Burant, S. S. Iyengar, J. Tomasi, M. Cossi, J. M. Millam, M. Klene, C. Adamo, R. Cammi, J. W. Ochterski, R. L. Martin, K. Morokuma, O. Farkas, J. B. Foresman, and D. J. Fox, Gaussian, Inc., Wallingford CT, 2016.
